# Cold Atmospheric Plasma and Plasma-Activated Medium Trigger RONS-Based Tumor Cell Apoptosis

**DOI:** 10.1038/s41598-019-50291-0

**Published:** 2019-10-02

**Authors:** Georg Bauer, Dominika Sersenová, David B. Graves, Zdenko Machala

**Affiliations:** 1 Institute of Virology, Medical Center, University of Freiburg, Freiburg, Germany; 2 Faculty of Medicine, University of Freiburg, Freiburg, Germany; 3 Division of Environmental Physics, Faculty of Mathematics, Physics and Informatics, Comenius University, Bratislava, Slovakia; 4 Department of Chemical and Biomolecular Engineering, University of California at Berkeley, Berkeley, California 94720 USA

**Keywords:** Biochemistry, Cancer, Cell biology, Chemical biology, Oncology, Physics

## Abstract

The selective *in vitro* anti-tumor mechanisms of cold atmospheric plasma (CAP) and plasma-activated media (PAM) follow a sequential multi-step process. The first step involves the formation of primary singlet oxygen (^1^O_2_) through the complex interaction between NO_2_^−^ and H_2_O_2._^1^O_2_ then inactivates some membrane-associated catalase molecules on at least a few tumor cells. With some molecules of their protective catalase inactivated, these tumor cells allow locally surviving cell-derived, extracellular H_2_O_2_ and ONOO^─^ to form secondary ^1^O_2_. These species continue to inactivate catalase on the originally triggered cells and on adjacent cells. At the site of inactivated catalase, cell-generated H_2_O_2_ enters the cell via aquaporins, depletes glutathione and thus abrogates the cell’s protection towards lipid peroxidation. Optimal inactivation of catalase then allows efficient apoptosis induction through the HOCl signaling pathway that is finalized by lipid peroxidation. An identical CAP exposure did not result in apoptosis for nonmalignant cells. A key conclusion from these experiments is that tumor cell-generated RONS play the major role in inactivating protective catalase, depleting glutathione and establishing apoptosis-inducing RONS signaling. CAP or PAM exposure only trigger this response by initially inactivating a small percentage of protective membrane associated catalase molecules on tumor cells.

## Introduction

Malignantly transformed cells (early stages of oncogenesis) are subject to elimination through selective apoptosis induction based on intercellular signaling, involving reactive oxygen and nitrogen species (RONS). This process, based on the HOCl or the ^⋅^NO/ONOO^−^ signaling pathway, has been previously described in detail^1–4^ (reviewed in refs^5–8^). Briefly, this apoptosis-inducing RONS signaling exploits the fact that transformed cells express unusually large concentrations of membrane-associated NADPH oxidase (NOX1), resulting in high concentrations of extracellular superoxide anions (O_2_^⋅−^). Some of this O_2_^⋅−^ can dismutate to H_2_O_2_ spontaneously or through the action of membrane-associated superoxide dismutase (SOD). DUOX-coded peroxidase derived either from the transformed cells themselves or from neighbouring nonmalignant cells, converts this H_2_O_2_ into HOCl and in this way initiates the HOCl signaling pathway^1,3,8^. The interaction between HOCl and NOX1-derived superoxide anions (O_2_^⋅−^) then yields ^⋅^OH radicals that cause lipid peroxidation. Alternatively, the ^⋅^NO/ONOO^−^ signaling pathway may be established through the reaction between superoxide anions (O_2_^⋅−^) and ^⋅^NO, resulting in the formation of ONOO^−^ H^+^ derived from membrane-associated proton pumps then leads to the formation of ONOOH, which spontaneously decomposes into ^⋅^NO_2_ and lipid peroxidating ^⋅^OH radicals^2,7^. If intracellular antioxidants - principally glutathione - are not abundant enough to completely repair this ^⋅^OH-mediated damage, a caspase-9/caspase-3 associated apoptosis sequence is initiated. We refer to this sequence of events leading to transformed cell apoptosis as intercelluar “HOCl signaling” or “NO/peroxynitrite signaling”, respectively. These two pathways seem to be mutually exclusive^7,9^.

Tumor cells escape this apoptotic signaling primarily through expression of membrane-associated catalase^9–13^. This catalase efficiently eliminates H_2_O_2_ near the cell membrane, and in this way prevents HOCl synthesis and apoptosis-inducing HOCl signaling. Membrane-associated catalase efficiently interferes also with ^⋅^NO/ONOO^−^ signaling through oxidation of ^⋅^NO and decomposition of ONOO^−^^7,9^. Interference of membrane-associated catalase with both signaling pathways therefore results in tumor survival. Inactivating tumor membrane-associated catalase is therefore a potentially attractive way to re-activate intercellular RONS-dependent apoptosis-inducing signaling^5,6,9,14^. It has been established that generation of singlet delta O_2_ (^1^O_2_) outside the cell membrane is capable of selectively inactivating membrane associated catalase, thereby re-activating intercellular RONS-driven apoptosis-inducing signaling^15^. It had been previously hypothesized that the observed selective anti-tumor effects of CAP might be related to this process involving ^1^O_2_^16–18^. The present article reports measurements and analyses testing this hypothesis.

The gaseous and liquid phase of cold atmospheric plasma (CAP) contains electrons, photons, as well as radical and nonradical reactive oxygen and nitrogen species (RONS) such as superoxide anions (O_2_^⋅−^), hydroperoxyl radicals (HO_2_^⋅^), atomic oxygen (O), hydrogen peroxide (H_2_O_2_), hydroxyl radicals (^⋅^OH), singlet oxygen (^1^O_2_), ozone (O_3_), nitric oxide (^⋅^NO), nitrogen dioxide (^⋅^NO_2_), peroxynitrite (ONOO^−^), nitrite (NO_2_^−^), nitrate (NO_3_^−^), dichloride radicals (Cl_2_^⋅−^) and hypochloride anions (OCl^−^) (summarized in refs^17,19–21^; please find detailed references under Suppl. Materials). CAP-derived RONS, created in the gas phase and transferred to liquid medium, represent a unique scenario of RONS chemical biology, based on variable life-times, free diffusion path lengths and multiple potentials of interactions.

The treatment of liquid media with CAP results in the generation of plasma-activated medium (PAM) that maintains the major biological effects of CAP, though it only contains long-lived species from CAP, such as nitrite (NO_2_^−^), nitrate (NO_3_^−^) and H_2_O_2_^22–25^. Girard *et al*.^23^ and Kurake *et al*.^24^ already recognized that a synergistic effect between H_2_O_2_ and NO_2_^−^ was essential for its biological effect. Both groups, as well as Jablonowski and von Woedtke^26^ suggested a potential role of peroxynitrite (ONOO^−^) that is generated through the interaction between NO_2_^−^ and H_2_O_2_^27,28^.

Cold atmospheric plasma (CAP) and plasma-activated medium (PAM) cause impressive antibacterial and antiviral effects, as well as beneficial effects for wound healing and the treatment of actinic keratosis^29–35^ (for review see refs^20,21^). CAP and PAM also establish promising antitumor effects *in vitro* and *in vivo*, in a very broad variety of tumor systems (reviewed in refs^20,21,32–41^). Clinical application of CAP for tumor therapy gave the first encouraging results in the absence of severe side effects^42^.

In most studies that directly compared tumor cells with nonmalignant cells, CAP and PAM were found to act selectively towards malignant target cells *in vitro* and *in vivo*. Only a few reports claimed nonselective apoptosis-inducing effects of CAP or PAM (reviewed in refs^17,21^; please find detailed references in Supplementary Material). It has been suggested that this discrepancy might be resolved by standardization of CAP and PAM doses and composition^21^.

The response of tumor cells *in vitro* and tumors *in vivo* from many different tumor systems indicates that CAP and PAM must be targeting a general principle of tumor cells. However, the mechanisms underlying the selective antitumor effects of CAP and PAM are still a matter of scientific debate.

Keidar’s group suggested that the increased concentration of aquaporins on tumor cells^43^ was the key determinant of selective antitumor action of CAP and PAM, as it should allow for an increased influx of CAP- or PAM-derived H_2_O_2_ into tumor cells, compared to nonmalignant cells^44,45^. This would then result in tumor cell apoptosis through direct intracellular effects mediated by H_2_O_2_, potentially by intracellular Fenton reaction.

Van der Paal *et al*.^46^ suggested that the decreased cholesterol content of tumor cells compared to nonmalignant cells was the determining factor for selective CAP and PAM action directed towards tumor cells, as cholesterol has a hampering effect on the ingress of ROS into cells.

Both models are based on the concept that ROS/RNS in CAP and PAM are ***directly*** responsible for the induction of cell death in the target cells. In both models, H_2_O_2_ is the major effector from CAP and the only effector from PAM. Both models did not consider, however, that tumor progression leads to a phenotype that is characterized by increased resistance to exogenous H_2_O_2_^47–51^. This tumor progression-associated resistance towards exogenous H_2_O_2_ is based on the expression of membrane-associated catalase^9–12^, Membrane-associated catalase protects tumor cells towards exogenous H_2_O_2_, but also oxidizes ^⋅^NO and readily decomposes peroxynitrite (ONOO^−^)^9,12^. Therefore, challenging cells with exogenous H_2_O_2_ or ONOO^−^ generally causes a much stronger apoptosis-inducing effect on nonmalignant cells and cells from early stages of tumorigenesis (transformed cells) than on tumor cells^12^. From this perspective, it seems that the mechanism of a purely H_2_O_2_-based apoptosis induction in tumor cells could not achieve the observed selectivity between tumor and nonmalignant cells. Therefore, nonmalignant cells that do not express this protective membrane-associated catalase system are much more vulnerable to exogenous H_2_O_2_ than tumor cells^9,12^, despite their lower number of aquaporins^43^.

The protective function of membrane-associated catalase of tumor cells^9,12^ (reviewed in refs^5,6,17,18^) is frequently neglected in the literature, as tumor cells in generally express less catalase than nonmalignant cells^12^. The finding of an overall low concentration of catalase in tumor cells is, however, not at all in contradiction to the strong expression of catalase on the membrane of tumor cells. Compared to the low concentration of catalase in the total volume of the tumor cells, the high local concentration of catalase on the spatially restricted site of the membrane is not relevant. Therefore it is not recognized when the catalase content of disaggregated cells is determined. However, its functional relevance towards extracellular ROS/RNS is a dominant factor for protection towards exogenous RONS effects, whereas the low intracellular catalase concentration enhances intracellular RONS effects.

Bauer and Graves^16^ suggested an alternative model to explain the selective action of CAP and PAM on tumor cells^16–18^. This model was derived from the analysis of apoptosis induction (as summarized above) in nonmalignant cells, transformed cells and tumor cells by defined RONS^9,12,15,52^. It took into account that the outer membrane of tumor cells, in contrast to nonmalignant cells, is characterized by the expression of NOX1, catalase and SOD^5,6,9,12,15,53,54^. It was shown that ^1^O_2_ derived from an illuminated photosensitizer caused local inactivation of a few (membrane-associated) catalase molecules^15^. Catalase inactivation then seemed to allow H_2_O_2_ and ONOO^−^ that are continuously generated by the tumor cells, to survive long enough to generate substantial amounts of secondary ^1^O_2_ through the reaction between H_2_O_2_ and ONOO^−^^55^. This was leading to further catalase inactivation and reactivation of intercellular apoptosis-inducing ROS signaling. Bauer and Graves^16^ and Bauer^17,18^ suggested that low concentrations of ^1^O_2_ from CAP, or derived through interaction of long-lived species in PAM, would interact with the surface of tumor cells, that carries NOX1, catalase and SOD, in the same way as shown before for extracellular ^1^O_2_ generated by a photosensitizer. Thus, CAP-and PAM-derived molecular species act as a trigger that utilizes the ability of tumor cells to induce a massive response, whereas it has no impact on the survival of nonmalignant cells. Nonmalignant cells lack the expression of NOX1, catalase and SOD on their surface. As long as the concentration of H_2_O_2_ is below an apoptosis-inducing level for nonmalignant cells, selective action of CAP and PAM towards tumor cells is feasible.

In a series of reconstitution experiments, Bauer confirmed that the long-lived species H_2_O_2_ and NO_2_^−^ that are found in CAP and PAM, are sufficient to generate ^1^O_2_ at concentrations that allow for initial local inactivation of a few catalase molecules^56^. Their reaction chain starts with ONOO^−^ formation through the reaction between NO_2_^−^ and H_2_O_2_^27,28^. ONOO^−^ and residual H_2_O_2_ then interact and generate primary ^1^O_2_^55^. This interaction is not direct, but seems to require several steps that utilize the decomposition of peroxynitrous acid (ONOOH) into nitrogen dioxide (^⋅^NO_2_) and ^⋅^OH radicals^57,58^, followed by the generation of hydroperoxyl radicals (HO_2_^⋅^) through the interaction between ^⋅^OH radicals and H_2_O_2_^59^. Finally, peroxynitric acid (O_2_NOOH) is generated through the interaction between ^⋅^NO_2_ and HO_2_^⋅^^60^. After deprotonation of O_2_NOOH, the resultant peroxynitrate (O_2_NOO^−^) decomposes and generates ^1^O_2_^60,61^ that causes local inactivation of catalase^15,62,63^. As a result, free tumor cell-derived H_2_O_2_ and ONOO^−^ allow for massive generation of secondary ^1^O_2_ in an autoamplificatory mode. The process is followed by catalase inactivation and subsequent activation of intercellular HOCl signaling. However, HOCl signaling can only lead to apoptosis induction when a sufficient influx of H_2_O_2_ into the cells had caused glutathione depletion, as glutathione/glutathione peroxidase-4 counteract the effects of ^∙^OH-mediated lipid peroxidation. Interestingly, central elements of the anti-tumor mechanism based on H_2_O_2_ influx via tumor cell aquaporins, as proposed by Yan *et al*.^44,45^, would be overlapping with the proposed scenario. With membrane-associated catalase inactivated by ^1^O_2_, tumor cells would be expected to allow aquaporin-mediated influx of H_2_O_2_ into the cells. The resultant depletion of intracellular glutathione seems to be a prerequisite for efficient apoptosis induction after lipid peroxidation by HOCl signaling. Therefore, as described by Yan *et al*.^44,45^, inhibition of aquaporins should strongly inhibit PAM-mediated apoptosis induction. Our experimental findings are consistent with their findings. However, intruding H_2_O_2_ by itself does not seem to be sufficient to trigger apoptosis induction, even if the intracellular glutathione level has been lowered. Rather, even in the situation of glutathione depletion, apoptosis induction in tumor cells required site-specific ^⋅^OH generation at the membrane through HOCl/O_2_^∙−^ interaction^56^. These findings also highlight the strength of site-directed ^⋅^OH effects compared to lower signal effectivity of random ^⋅^OH generation through Fenton chemistry.

In this paper, the mechanisms proposed by reconstitution experiments are verified by using a CAP source operating in ambient air in either streamer corona or transient spark regimes. The malignant cells are treated either “directly” or indirectly via PAM. We were aware that “direct” treatment of cells with CAP also implies that CAP-derived molecular species are first confronted with the overlaying medium, which may react with highly reactive species from CAP and thus select for longer-lived species

## Materials and Methods

### Materials

Table 1 presents a summary of enzyme inhibitors, reactive species scavengers, reactive species donors, mimetics, and antibodies used in the present study to elucidate apoptotic and protective mechanisms.

**Table 1:** Summary of enzyme inhibitors, reactive species scavengers, reactive species donors, mimetics, and antibodies used in the present study to elucidate apoptotic and protective mechanisms.

Purpose	Compound name	Compound abbreviation and standard working concentration
Singlet oxygen scavenger	Histidine	HIS 2 mM
Peroxynitrite decomposition catalyst	5-, 10-, 15-, 20-Tetrakis(4-sulfonatophenyl)porphyrinato iron(III) chloride	FeTPPS 25 µM
NOX1 inhibitor	4-(2-Aminoethyl) benzenesulfonyl fluoride	AEBSF 100 µM
HOCl scavenger	Taurine	TAU 50 mM
Aquaporin inhibitor	AgNO_3_	Ag^+^ 5 µM
Catalase inhibitor	3-aminotriazole	3-AT 25 mM
Catalase donation (bovine liver catalase)	Catalase	CAT 10 - 1000 U/ml
glutathione synthesis inhibitor	Buthionine sulfoximine	BSO 10 - 50 µM
^⋅^OH scavenger	Manitol	MANN 20 mM
^⋅^OH scavenger	Dimethylthiourea	DMTU 20 mM
NO donor	Dieethylamine NONOate	DEA NONOate 0.5 mM
HOCl donor	Sodium oxychloride	NaOCl as indicated
Generation of H_2_O_2_	Glucose oxidase	GOX as indicated
Nitric Oxide Synthase inhibitor	N-omega-nitro-L-arginine methylester hydrochloride	L-NAME 2.4 mM
Proton pump inhibitor	Omeprazole	
Peroxidase inhibitor	4-Aminobenzoyl hydrazide	ABH 150 µM
Caspase-3 inhibitor		Z-DEVD-FMK 50 µM
Caspase-8 inhibitor		Z-IETD-FMK 25 µM
Caspase-9 inhibitor		Z-LEHD-FMK 25 µM
SOD mimetics	Mn(III) 5,10,15,20-tetrakis(N-methylpyridinium-2-yl)porphyrin and Mn (III) meso-tetrakis(N-ethylpyridinium-2-yl)porphyrin	MnTM-2PyP and MnTE-2-PyP 20 µM
Mn-SOD donation (*E. coli*)	Manganese superoxide dismutase	Mn-SOD 100 U/ml
ONOO^−^ decomposition catalyst and O2^−^ scavenger	Fe(III)tetrakis(1-methyl-4-pyridyl)porphyrin pentachlorideporphyrin pentachloride	FeTMPyP 25 µM
Catalase mimetic	chloro([2,2′-[1,2-ethanediylbis[(nitrilo-κN)methylidyne]]bis[6-methoxyphenolato-κO]]]-manganese	EUK-134 20 µM
Antibody for human superoxide dismutase (SOD)		cb 0989 (binding and neutralizing) cb 0987 (binding without neutralization)

The NOX1 inhibitor 4-(2-Aminoethyl)benzenesulfonyl fluoride (AEBSF), the aquaporin inhibitor AgNO_3_, the catalase inhibitor 3-aminotriazole (3-AT), the inhibitor of glutathione synthesis buthionine sulfoximine (BSO), catalase from bovine liver, the ^⋅^OH radical scavenger dimethylthiourea, NaOCl (for the generation of HOCl), the fast decaying ^⋅^NO donor dieethylamine NONOate (DEA NONOate), glucose oxidase (GOX), the singlet oxygen (^1^O_2_) scavenger histidine, the ^⋅^OH radical scavenger mannitol, the NOS inhibitor N-omega-nitro-L-arginine methylester hydrochloride (L-NAME), the proton pump inhibitor omeprazole, the HOCl scavenger taurine, Mn-SOD from *E. coli*, were obtained from Sigma-Aldrich (Schnelldorf, Germany).

The peroxidase inhibitor 4-Aminobenzoyl hydrazide (ABH) was obtained from Acros Organics (Geel, Belgium). Inhibitors for caspase-3 (Z-DEVD-FMK), caspase-8 (Z-IETD-FMK) and caspase-9 (Z-LEHD-FMK) were obtained from R&D Systems (Wiesbaden-Nordenstadt, Germany).

The ONOO^−^ decomposition catalyst 5-, 10-, 15-, 20-Tetrakis(4-sulfonatophenyl)porphyrinato iron(III) chloride (FeTPPS), the SOD mimetics Mn(III) 5,10,15,20-tetrakis(N-methylpyridinium-2-yl)porphyrin (MnTM-2PyP) and Mn (III) meso-tetrakis(N-ethylpyridinium-2-yl)porphyrin (MnTE-2-PyP), as well as Fe(III)tetrakis(1-methyl-4-pyridyl)porphyrin pentachlorideporphyrin pentachloride (FeTMPyP, a ONOO^−^ decomposition catalyst and superoxide anion scavenger) were obtained from Calbiochem (Merck Biosciences GmbH, Schwalbach/Ts, Germany).

The catalase mimetic EUK-134 [chloro([2,2‘-[1,2-ethanediylbis[(nitrilo-κN)methylidyne]]bis[6-methoxyphenolato-κO]]]-manganese was a product of Cayman (Ann Arbor, Michigan, U.S.A.) and was obtained from Biomol (Hamburg, Germany).

Single domain antibodies directed towards human SOD (cb 0989 (binding and neutralizing) and cb 0987 (binding without neutralization) have been recently described^53^.

All small interfering RNAs (siRNAs) used in this study were obtained from Qiagen (Hilden, Germany) and are described in detail under Methods.

Detailed information on inhibitors has been previously published^2,9–11,64,65^. The site of action of inhibitors and scavengers has been presented in detail in the supplementary material of refs^64,65^.

### Cells and media for cell culture

The human gastric adenocarcinoma cell line MKN-45 (ACC 409) (established from the poorly differentiated adenocarcinoma of the stomach (medullary type) of a 62 year-old woman), was purchased from DSMZ, Braunschweig, Germany. MKN-45 were cultured in RPMI 1640 medium, containing 10% fetal bovine serum (FBS).

The human Ewing sarcoma cell line SKN-MC and human neuroblastoma cell line SHEP were obtained from Dr. J. Roessler, Dep. of Pediatrics and Adolescent Medicine, University Medical Center Freiburg. SKN-MC cells and SHEP grow in monolayer and were kept in Eagles MEM, containing 5% FBS and supplements as described above.

The human HPV-16-positive cervix adenocarcinoma cell line SIHA was obtained from the American type culture collection. The cells were kept in Eagles MEM, containing 5% FBS and supplements.

The human non-malignant diploid fibroblasts Alpha-1 were isolated in the Diagnostic Unit of our Institute and have been described in Riethmüller *et al*.^15^.

Fetal bovine serum (Biochrom, Berlin, Germany) was heated for 30 minutes at 56 °C prior to use. Medium was supplemented with penicillin (40 U/ml), streptomycin (50 µg/ml), neomycin (10 µg/ml), moronal (10 U/ml) and glutamine (280 µg/ml). Care was taken to avoid cell densities below 300 000/ml and above 10^6^/ml.

## Methods

### The plasma sources

**Figure 1:**
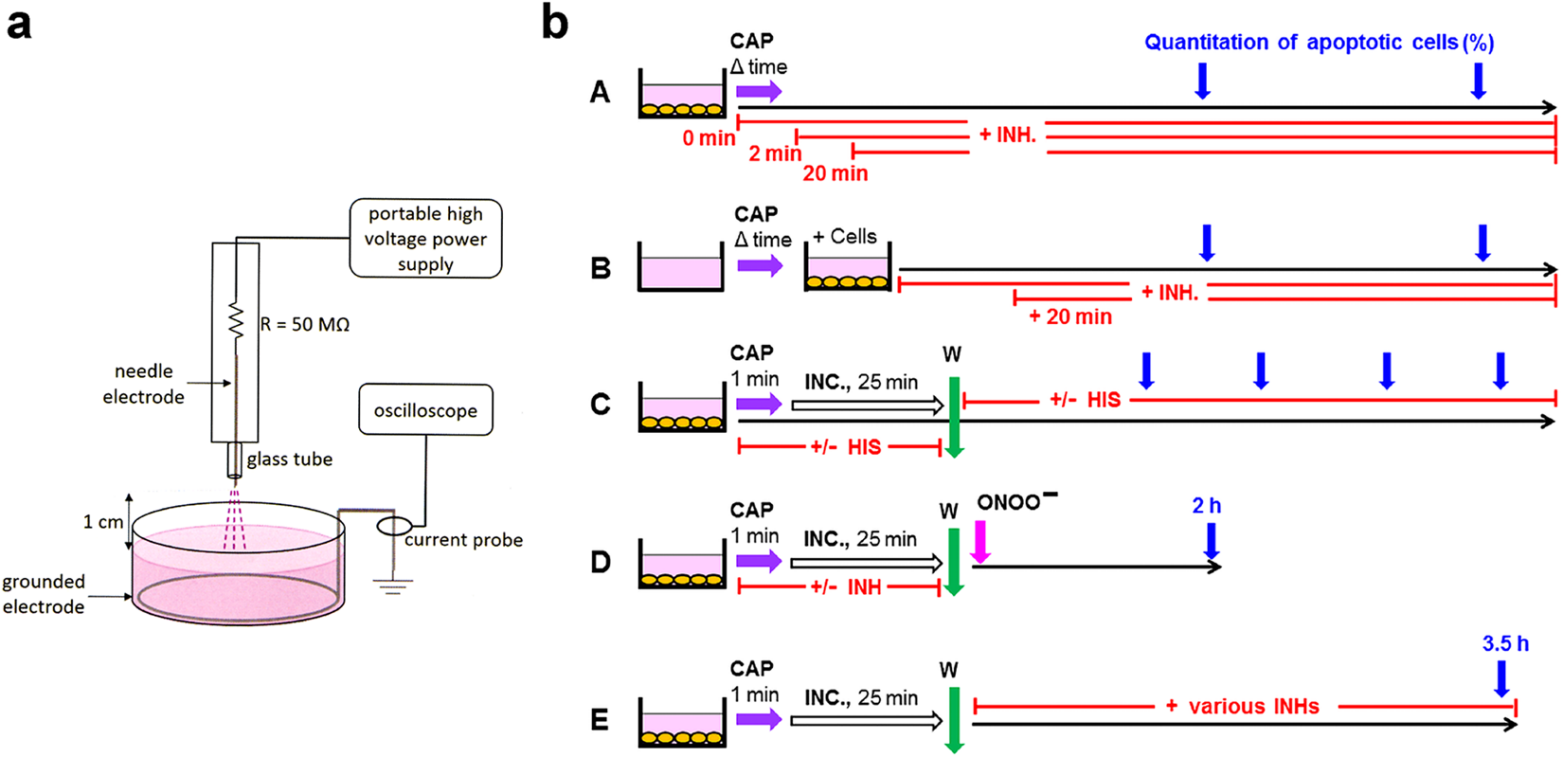
The plasma source and the experimental procedures used in this study. (**a**) Schematic drawing of the portable plasma source used in this study. The portable plasma source enabled operation in the corona and transient spark regime, as described under Methods^69–71^. (**b**) Experimental procedures. A. Tumor cells were treated with CAP for varying times, combined with the addition of specific inhibitors/scavengers (INH) at defined time points. Apoptosis induction was quantified at different times. B. The same princples as shown under A were applied, except that medium was treated with CAP in the absence of cells, and was then transferred to the cells. C. Tumor cells were treated with CAP for 1 min, followed by 25 min incubation in the same medium. After a washing step, incubation was continued in fresh medium. The ^1^O_2_ scavenger histidine was present either before or after the washing step. Apoptosis induction was determined kinetically. D. Tumor cells were treated with CAP (or PAM) in the absence or presence of defined inhibitors/scavenger and incubated for 25 minutes in the same medium. After a washing step, the degree of inactivation of membrane-associated catalase was determined through a ONOO^−^ challenge and determination of ONOO^−^ mediated apoptosis induction^12^. E. Tumor cells were treated with CAP for 1 min and incubated in the same medium for 25 min. After a washing step, further cultivation of the cells was performed in the absence or presence of defined inhibitors/scavengers (INHs) that allowed to define the molecular species involved in apoptosis-inducing signaling after catalase inactivation.

Portable air plasma ‘corona pen’ plasma source used here employs a neon-sign transformer with a rectifier and a high voltage multiplier and was developed in the framework of the frugal plasma biotech applications^66^ (Fig. 1a). A high voltage needle electrode was inserted in a quartz tube. A DC-positive streamer corona discharge was generated on the needle electrode in ambient air, in a geometry similar to the discharge previously presented in^67,68^. The grounded electrode was a tin wire submerged in the cell culture medium at the bottom of the container. The distance of the needle tip to the medium surface was kept at 1 cm. The plasma discharge was directly hitting the liquid surface of the medium, as shown in Fig. 1(a). The discharge voltage was kept at 10.7 kV and the maximum streamer pulse current was typically 17 mA with a pulse frequency of 10 kHz. The streamer corona discharge generates RONS, such as O_3_, NOx, and ^⋅^OH radicals at very low deposited power (<0.1 W).

The portable plasma source also enabled operation in the transient spark regime, described in greater details in^69–71^. In the same way, the grounded wire was submerged in the medium, the high voltage needle electrode was 1 cm apart from its surface and the discharge was directly hitting the liquid surface. Higher voltage was applied (13.3 kV) to enable the plasma discharge to operate at the streamer-to-spark transition regime, with high (15 A amplitude) but very short (<100 ns) spark current pulses with a repetitive frequency of 1.1 kHz. The transient spark discharge also generates RONS, especially NOx, OH radicals and H_2_O_2_ at very relatively low deposited power (~1–2 W).

### Treatment of cells with cold atmospheric plasma (CAP)

All treatments were performed in 24 well tissue culture clusters, 1 ml of medium and a grounded electrode. MKN-45 cells were used at a density of 125 000 cells/ml. The cells remain in suspension and only few cells attach firmly. SIHA; Alpha-1, SHEP and SKN-MC cells *we*re treated as monolayers (50 000 cells/assay) as soon as the cells had firmly attached.

Standard treatment with CAP was by the streamer CORONA regime, with a distance of the plasma source from the top of the medium of 1 cm. Typical electrical parameters were voltage 10.7 kV, pulse amplitude 17 mA, and pulse frequency 10 kHz. The standard time of treatment was 1 min, unless otherwise indicated.

After treatment with CAP, the cells were either further incubated at 37 °C for the indicated times or subjected to washing steps and resuspension in fresh medium, depending on the protocol of the experiments. These manipulations, which were essential for the analysis, are specified in the legends of the respective figures. The final goal was to determine the percentage of apoptotic cells induced by the treatment.

In some cases (Supplementary Figs 7–10), CAP treatment was in the transient SPARK regime, with typical electrical parameters voltage 13.3 kV, pulse amplitude 15 A, and pulse frequency 1.1 kHz.

Corona discharges are known to induce “ionic wind”, i.e. a gas flow is induced by the motion of ions in the electric field. In our case the ionic wind blows in the direction from the needle towards the liquid surface and reaches velocities of a few m/s. This consequently induces some medium convection and mixing, dependent on the electrical discharge parameters, as well as the liquid container geometry and the liquid volume. In the transient spark regime, the ionic wind is coupled with the hydrodynamic pressure waves due to the short, strong current pulses, both of which can induce some convections in the liquid. These phenomena and their significance are subject of our future investigations. In all experiments presented in this paper, the electrical discharge parameters, as well as geometries and liquid containers and volumes were kept the same to eliminate their potential influence on the biochemical and biological responses.

### Generation and application of plasma-activated medium (PAM)

Complete medium without cells was treated with CAP for 1 min, unless otherwise specified. After 10 min, PAM was added to the cells that had been prepared at higher cell density, to reach a final concentration of PAM between 80–50%, as indicated. In some experiments, PAM was first serially diluted and then equal volumes of the dilution steps and cells of double standard density were mixed.

### Apoptosis induction mediated by exogenous ONOO^−^

Treatment with exogenous ONOO^−^ allows to quantitatively monitor the activity of membrane-associated catalase as this enzyme decomposes exogenous ONOO^−^, whereas intracellular catalase cannot reach exogenous ONOO^−^ before the compound attacks the cell membrane^12^. After the indicated pretreatments at a density of 125 000 cells/ml, the cells were washed several times through centrifugation and resuspension in fresh medium and then were seeded at a density of 12 500 cells/100 µl. The cells received 100 µM AEBSF to prevent autocrine apoptosis induction and negative interference of cell-derived H_2_O_2_ with ONOO^−^. ONOO^−^ was diluted in ice-cold PBS immediately after controlled thawing and was rapidly applied to the cells. This approach allows to focus on apoptosis induction by exogenous ONOO^−^, which is an indication for the inactivation of membrane-associated catalase. Apoptosis criteria were used as defined below.

### Determination of the percentage of apoptotic cells

After the indicated time of incubation at 37 °C and 5% CO_2_, the percentage of apoptotic cells was determined by inverted phase contrast microscopy based on the classical criteria for apoptosis, i.e., nuclear condensation/fragmentation or membrane blebbing^9,65,72,73^. The characteristic morphological features of intact and apoptotic cells, as determined by inverted phase contrast microscopy have been published^9,14,65,74,75^. At least 200 neighbouring cells from randomly selected areas were scored for the percentage of apoptotic cells at each point of measurement. Control assays ensured that the morphological features ‘nuclear condensation/fragmentation’ as determined by inverse phase contrast microscopy were correlated to intense staining with bisbenzimide and to DNA strand breaks, detectable by the TUNEL reaction^2,14,74,75^. A recent systematic comparison of methods for the quantitation of apoptotic cells has shown that there is a perfect coherence between the pattern of cells with condensed/fragmented nuclei (stained with bisbenzimide) and TUNEL-positive cells in assays with substantial apoptosis induction, whereas there was no significant nuclear condensation/fragmentation in control assays^14,65^. Further controls ensured that ROS-mediated apoptosis induction was mediated by the mitochondrial pathway of apoptosis, involving caspase-9 and caspase-3^4,14^.

### Knockdown by treatment with specific small interfering ribonucleic acids (siRNAs)

Techniques and siRNA described in this and the next subchapter are identical to those described in refs^3,4,9,12,13,76^ and highly reproducible.

SiRNAs were obtained from Qiagen (Hilden, Germany).

#### The following siRNAs were used

Control siRNA which does not affect any known target in human and murine cells (siCo):

sense: r(UUCUCCGAACGUGUCACGU)dTdT,

antisense: CGUGACACGUUCGGAGAA)dTdT;

### SiRNA directed towards human NADPH oxidase-1 (NOX1)

custom-made siRNA directed towards NADPH oxidase-1 variant a (**siNOX1-a)**: target sequence: CCG ACA AAT ACT ACT ACA CAA

sense: r(GAC AAA UAC UAC UAC ACA A)dTdT,

antisense: r(UUG UGU AGU AGU AUU UGU C)dGdG;

SiRNAs were dissolved in suspension buffer supplied by Qiagen at a concentration of 20 µM. Suspensions were heated at 90 °C for 1 minute, followed by incubation at 37 °C for 60 minutes. Aliquots were stored at −20 °C.

Before transfection, 88 µl of medium without serum and without antibiotics were mixed with 12 µl Hyperfect solution (Qiagen) and the required volume of specific siRNA or control siRNA to reach the desired concentration of siRNA during transfection (the standard concentration of siRNA was 24 nM for MKN-45 cells). The mixture was treated by a Vortex mixer for a few seconds and then allowed to sit for 10 minutes. It was then gently and slowly added to 300,000 MKN-45 cells in 1 ml RPMI-1640 medium containing 10% FBS and antibiotics (12-well plates). The cells were incubated at 37 °C in 5% CO_2_ for 24 hours. Transfected cells were centrifuged and resuspended in fresh medium at the required density before use.

#### Determination of the efficiency of siRNA-mediated knockdown

The siRNA transfection system as described above had been optimized to allow a reproducible transfection efficiency of more than 95% of the cells and to avoid toxic effects (Bauer, unpublished data).

The efficiency of knockdown by siNOX1 was based on functional SOD-dependent quantitative assay^3,76^ and was more than 90%.

### Statistical analysis

In all experiments, assays were performed in duplicate. Quantitative data are presented as means ± standard deviations. The statistical analysis comprised the comparison of groups such as assay without apoptosis induction/assay with apoptosis inducer or assay without inhibitor/assay with inhibitor. Therefore, the differences between two groups were analyzed by Student’s t-test (two-tailed), with N = 500 in all tests, and double checked with the Yates continuity corrected chi-square test. The confidence interval used was 95%. P < 0.01 was defined as “significant”; P < 0.001 as “highly significant”. The modules for the calculation of the tests were taken from https://www.quantitativeskills.com/sisa/statistics/t-test.htm (t test) and from http://www.quantpsy.org/chisq/chisq.htm (Chi-square test).

### Strategy and design of our analysis

**Figure 2:**
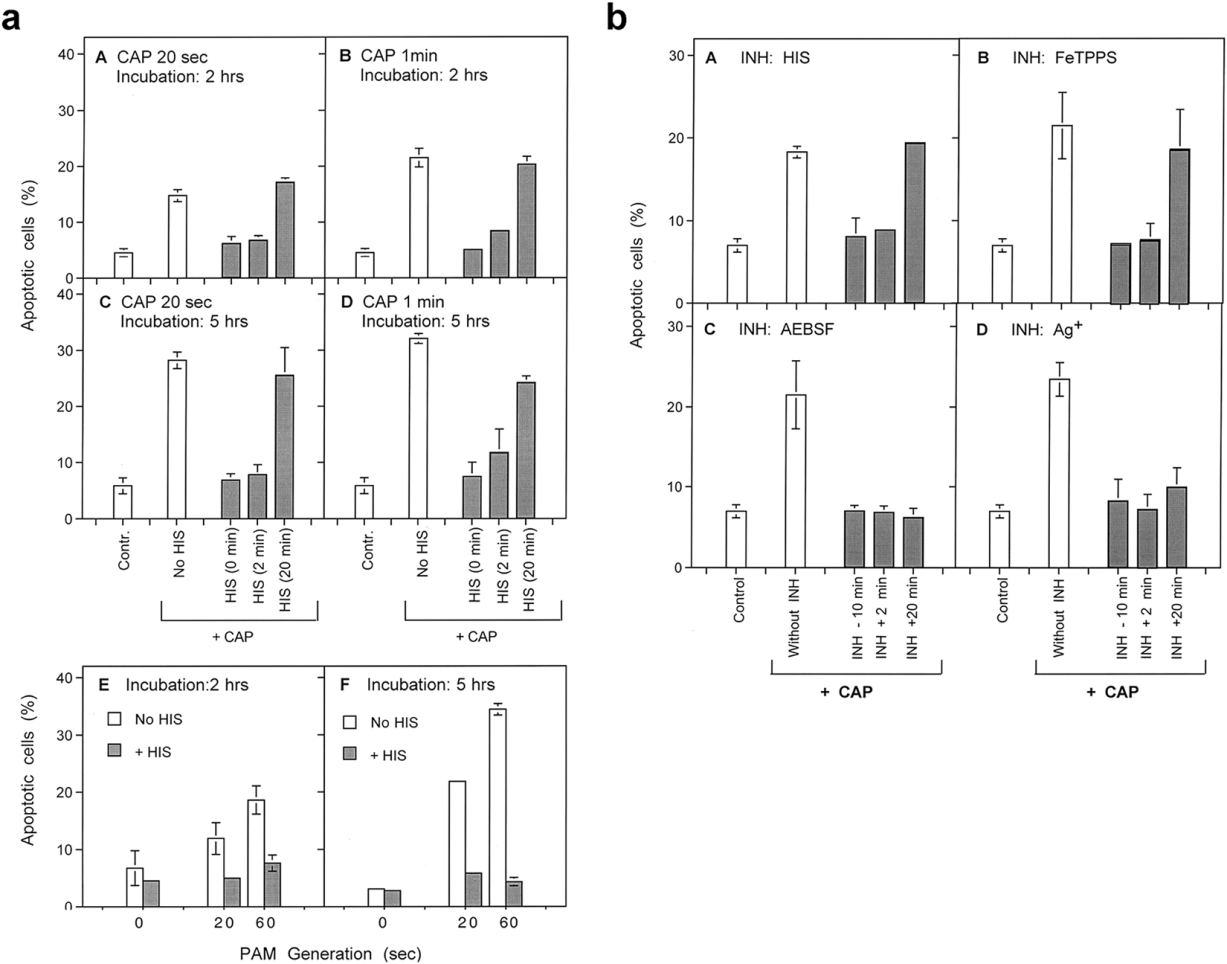
CAP and PAM-mediated apoptosis induction in tumor cells. (**a**) CAP- and PAM-mediated apoptosis induction in tumor cells is dependent on singlet oxygen (^1^O_2_). A-D: Treatment with CAP. MKN-45 tumor cells in medium were treated with CAP for either 20 sec or 1 min, either in the absence of the ^1^O_2_ scavenger histidine (HIS) (2 mM) or with HIS addition at the indicated times. Control assays were not treated with CAP. The percentages of apoptotic cells were determined after 2 and 5 h. E,F: The effect of PAM. Medium was treated with CAP for 20 or 60 sec, or not treated. It was then added to an equal volume of MKN-45 cells at double standard density, in the absence or presence of HIS. The final volume was 100 µl. The percentages of apoptotic cells were determined after 2 and 5 h. These results show that ^1^O_2_ generated by long-lived species that are generated through treatment of medium with CAP are sufficient to trigger apoptosis induction in tumor cells. The ^1^O_2_-dependent process seems to be completed within less than half an hour. Statistical analysis: Apoptosis induction by CAP or PAM under all conditions is highly significant (p < 0.001). The inhibition by histidine added a 0 or 2 min is highly significant (p < 0.001) in all assays. The differences between the effect of histidine added at 20 min or 0/2 min is highly significant (p < 0.001). (**b**) CAP-mediated apoptosis induction in tumor cells: Dependency on singlet oxygen (^1^O_2_), peroxynitrite (ONOO^−^), superoxide anions (O_2_^⋅−^) and aquaporins. MKN-45 tumor cells were treated with CAP in the absence or presence of the indicated scavengers/inhibitors (A: the ^1^O_2_ scavenger histidine (HIS) (2 mM); B: the ONOO^−^ decomposition catalyst FeTPPS (25 µM); C: the NOX1 inhibitor AEBSF (100 µM); D: the aquaporine inhibitor Ag^+^ (5 µM)). Scavengers/inhibitors had been added either 10 min before CAP treatment, or 2 min or 20 min after CAP treatment. The percentages of apoptotic cells were determined after 4.5 h. These results show that CAP-mediated apoptosis induction in tumor cells involves a very fast ^1^O_2_ - and ONOO^−^-dependent process, as well as longer-lasting processes in which superoxide anions and aquaporins are involved. Statistical analysis: Apoptosis induction by CAP is highly significant (p < 0.001). Inhibition of CAP-mediated apoptosis induction by histidine and FeTPPS added at −10 or 2 min, as well as inhibition by AEBSF or Ag^+^, added at all time points, is highly significant (p < 0.001).

Our study was primarily based on the quantitation of apoptosis induction in human tumor cells by CAP and PAM *in vitro*. The application of defined inhibitors and scavengers at different time points was used to pinpoint the molecular species that determined the different steps in this scenario. The basic experimental procedures used in this analysis are schematically described in Fig. 1(b). Treatment of the cells with CAP for varying time, combined with addition of inhibitors (regime A, Fig. 1(b)) gave first information on the role of central players like ^1^O_2_, O_2_^⋅−^, ONOO^−^ and aquaporins (Results will be shown in Fig. 2).

**Figure 3:**
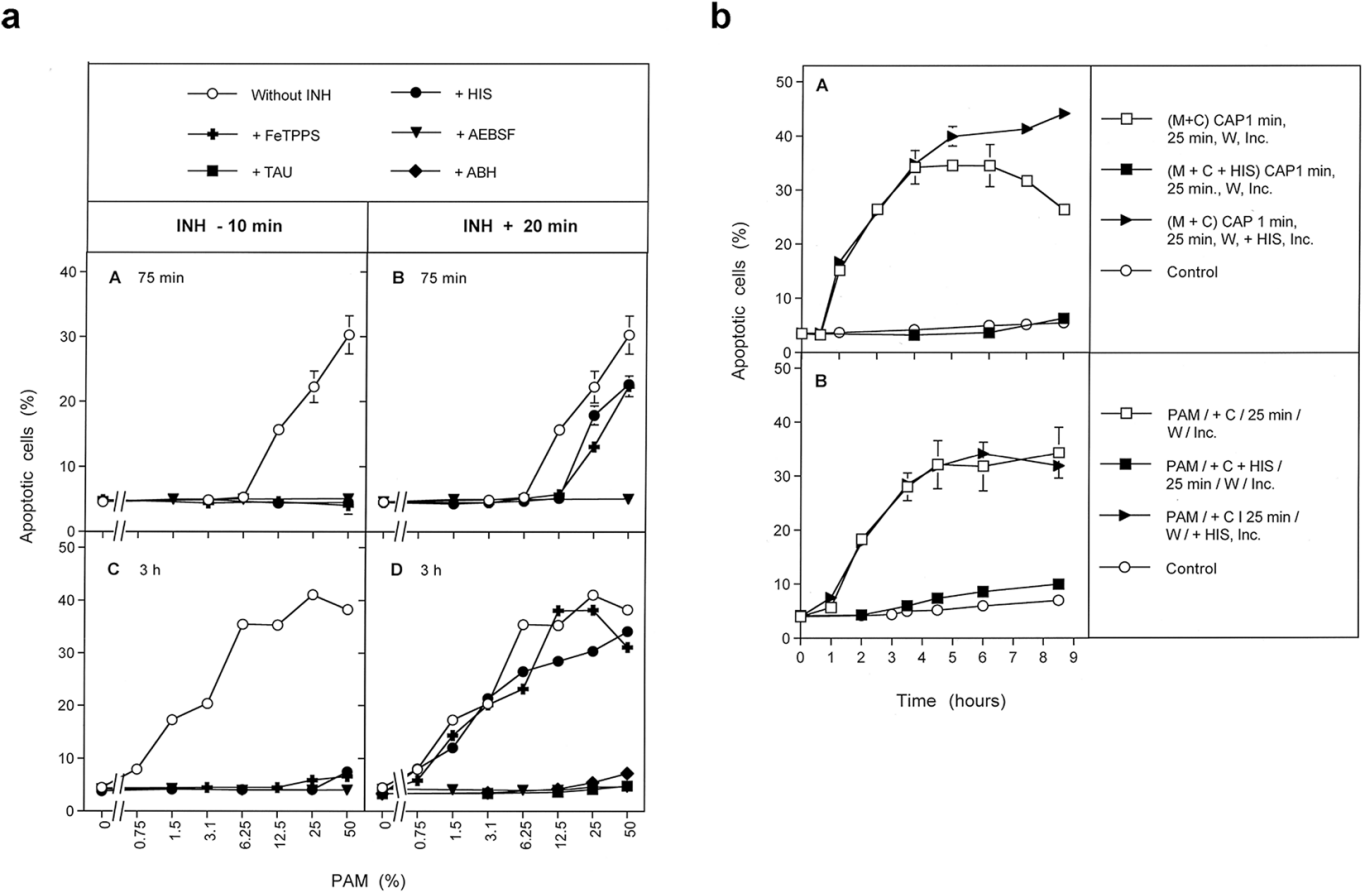
PAM-mediated apoptosis induction in tumor cells. (**a**) Inhibition profile of PAM-mediated apoptosis induction in tumor cells. Medium was treated with CAP for 1 min in the absence of cells for the generation of PAM. PAM was serially diluted and added at equal volumes to MKN-45 cells to reach a final volume of 100 µl and cell density of 12 500 cells/100 µl. The indicated inhibitors/scavengers (INH) (the ^1^O_2_ scavenger histidine (HIS) (2 mM), the ONOO^−^ decomposition catalyst FeTPPS (25 µM); the NOX1 inhibitor AEBSF (100 µM); the HOCl scavenger taurine (TAU) (50 mM) and the the peroxidase inhibitor 4-aminobenzoyl hydrazide (ABH) (150 µM)) had been either added 10 min before addition of PAM to the cells or 20 min after addition of PAM. The percentages of apoptotic cells were determined after 75 min (A,B) and 3 h (C,D), as indicated. PAM causes concentration-dependent apoptosis induction in tumor cells. This seems to involve a very short initial process (mediated by ^1^O_2_ and ONOO^−^) and subsequent HOCl signaling, as seen by the involvement of peroxidase and HOCl. Superoxide anions (O_2_^⋅−^) may have a dominant role in both processes. Statistical analysis: Apoptosis induction by 12.5 percent PAM and higher concentrations, determined at 75 min, and by 1.5% and higher, measured at 3 hrs is highly significant (p < 0.001.) Inhibition of PAM-mediated apoptosis induction by all inhibitors, added at −10 min is highly significant (p < 0.001).The differences of the inhibitors added at the two time points are highly signficant (p < 0.001). (**b**) Kinetics of CAP- and PAM-mediated apoptosis induction in tumor cells. A. MKN-45 cells in medium (M + C) were treated with CAP for 1 min and were further incubated at 37 °C for 25 min (“25 min”). The cells were washed (W) (two cycles) and resuspended in fresh medium. Parallel assays contained histidine (HIS) (2 mM) during CAP treatment and the subsequent 25 min incubation, or received HIS after the washing step. Control assays were not treated with CAP. The percentages of apoptotic cells were determined kinetically during the incubation (Inc.) that followed the washing step. Time point zero is defined by resuspension of the cells in fresh medium after the washing step. B. PAM was generated through treatment of medium without cells with CAP for 1 minute. PAM was added to MKN-45 cells (90 µl PAM plus 10 µl cells of 10 fold standard density) and further incubated at 37 °C for 25 min. The cells were washed (2 cycles) and resuspended in fresh medium. Parallel assays contained histidine either during the 25 min incubation step, or received histidine after the washing step. Control assays were not treated with CAP. The percentages of apoptotic cells were determined kinetically. These results show that CAP- and PAM-mediated apoptosis induction in tumor cells are dependent on singlet oxygen and show similar kinetics. Statistical analysis: Apoptosis induction mediated by CAP after 1 h and later, and by PAM after 2 h and later is highly significant (p < 0.001). The inhibitory effect of histidine added during CAP or PAM treatment is highly significant (p < 0.001).

**Figure 4:**
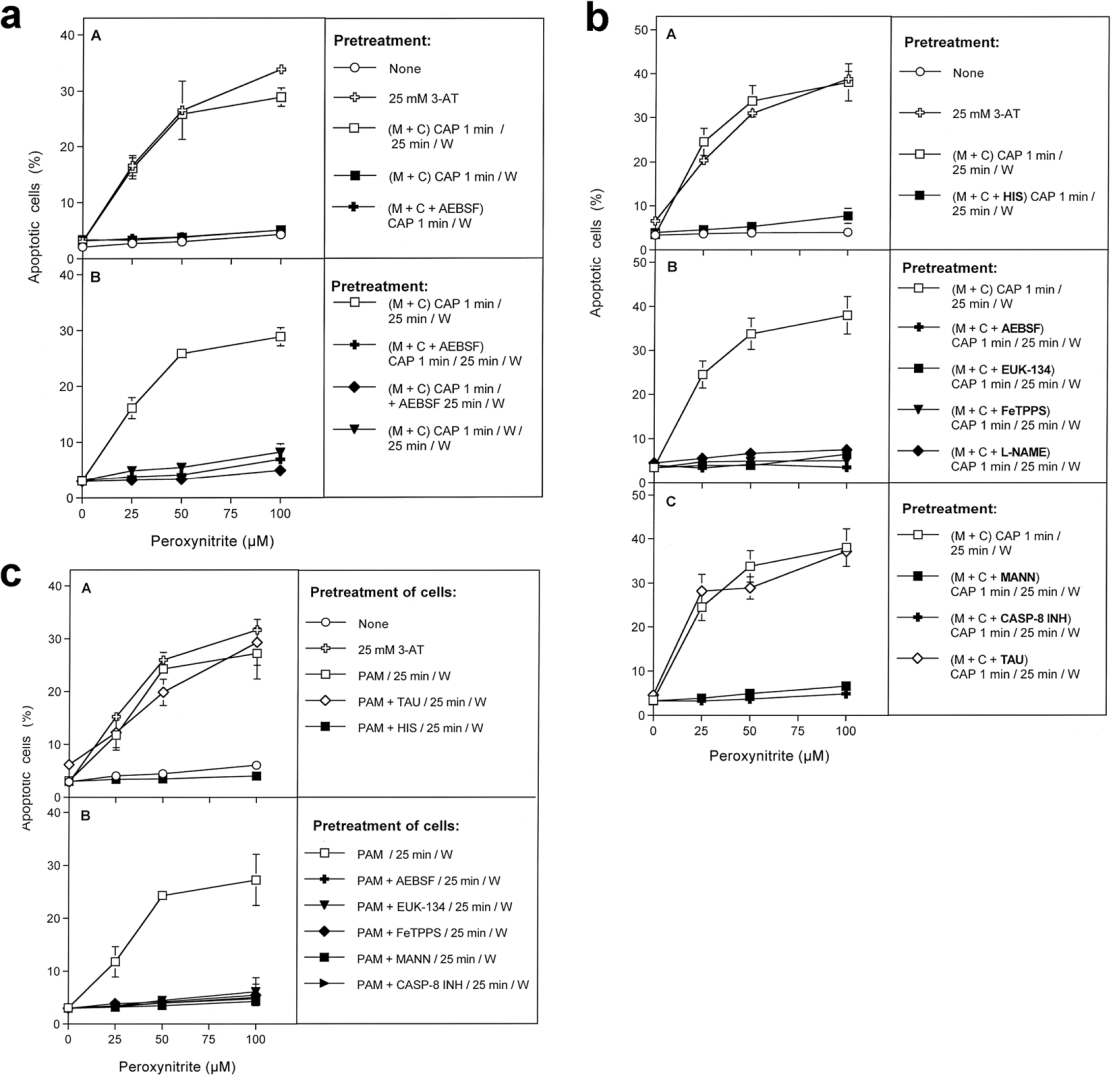
Inactivation of membrane-associated catalase of tumor cells by CAP and PAM. (**a**) Basic experiments. A. MKN-45 cells remained untreated (open circle) or were treated with 25 mM of the catalase inhibitor 3-AT (open cross), before they were challenged with peroxynitrite (ONOO^−^) in the presence of the NOX1 inhibitor AEBSF (100 µM). In parallel, cells were treated with CAP for 1 min, followed by 25 min in the same medium before they were washed (W), resuspended in fresh medium containing 100 µM AEBSF, and were challenged with ONOO^−^ (open square). Alternatively, cells in control medium (closed square) and cells in medium containing 100 µM AEBSF (closed cross) were treated with CAP for 1 min, followed directly by a washing step (W) and immediate challenge with ONOO^−^ in the presence of 100 µM AEBSF. B. Cells in medium were treated with CAP for 1 min, followed by an incubation step of 25 min in the same medium, before the cells were washed (W) and resuspended in fresh medium (open square). This regime was modified by adding AEBSF (100 µM) either before CAP treatment (closed cross) or at the beginning of the 25 min incubation (closed diamond), or by washing the cells immediately after the CAP treatment and incubating them for 25 min in fresh medium, followed by a second washing step (closed triangle). All assays were challenged with increasing concentrations of ONOO^−^, in the presence of 100 µM AEBSF. The percentages of apoptotic cells were determined 2 h after addition of ONOO^−^. The results show that catalase inhibition by 3-AT or treatment of the cells with CAP for 1 min, followed by 25 min of incubation in the same medium caused sensitization towards ONOO^−^, indicative for catalase inactivation. Catalase inactivation through CAP treatment required that the long-lived species generated by CAP need to interact with the tumor cells during the 25 min incubation step and that NOX1 was not inhibited during this incubation step. Statistical analysis: Apoptosis induction by all concentrations of ONOO^−^, in the presence of 3-AT or after pretreatment with CAP is highly significant (p < 0.001). The inhibitory effect of AEBSF is highly significant (p < 0.001). (**b**) Elucidation of the biochemical mechanism of CAP-mediated inactivation of catalase. (A) MKN-45 cells were not pretreated or treated with 25 mM of the catalase inhibitor 3-aminotriazole (3-AT), received 100 µM of the NOX1 inhibitor AEBSF and were then challenged by increasing concentrations of peroxynitrite (ONOO^−^). The percentages of apoptotic cells were determined 2 h after the ONOO^−^ challenge. In parallel, MKN-45 cells were treated with CAP for 1 min, followed by 25 min incubation in the same medium and three cycles of washing (W) (“(M + C) CAP 1 min/25 min/W”). In addition, the same regime was performed in the presence of the ^1^O_2_ scavenger histidine (HIS) during CAP treatment and the 25 min incubation step (“(M + C + HIS) CAP 1 min/25 min/W”) 100 µM AEBSF were then added and the cells were challenged with increasing concentrations of ONOO^−^. The percentages of apoptotic cells were determined two hours after addition of ONOO^−^. B,C: CAP treatment, 25 min incubation in the same medium, washing and ONOO^−^challenge were performed as described under A. This regime was modified by the presence of the following compounds during CAP treatment and the incubation step: the NOX inhibitor AEBSF (100 µM); the catalase mimetic EUK-134 (20 µM), the ONOO^−^ decomposition catalyst FeTPPS (25 µM); the NOS inhibitor L-NAME (2.4 mM); the ^⋅^OH radical scavenger mannitol (MANN) (20 mM), caspase-8 inhibitor (25 µM) and the HOCl scavenger taurine (TAU) (50 mM. These findings show that CAP-mediated inactivation of tumor cell protective catalase requires the action of ^1^O_2_, NOX1-derived O_2_^⋅−^, H_2_O_2_, ONOO^−^, NOS-derived ^⋅^NO, ^⋅^OH radicals and caspase-8, whereas it does not require HOCl. Statistical analysis: Apoptosis induction by *ONOO*^−^ after CAP treatment is highly significant *(p* < *0*.*001)*. *The inhibitory effects of all inhibitors*,*with the exception of taurine*, *are highly significant* (*p* < *0*.*001*). (**c**) *Inactivation of protective catalase of tumor cells by PAM*. PAM was generated by CAP treatment of the medium without cells for 1 min. Tumor cells were treated with 50% of PAM for 25 min at 37 °C, in the absence of inhibitors or in the presence of 50 mM of the HOCl scavenger taurine (TAU), 2 mM of the ^1^O_2_ scavenger histidine (HIS), 100 µM of the NOX1 inhibitor AEBSF, 20 µM of the catalase mimetic EUK-134, 25 µM of the ONOO^−^ decomposition catalyst FeTPPS, 20 mM of the ^⋅^OH radical scavenger mannitol (MANN) or 25 µM of caspase-8 inhibitor. Incubation was followed by three cycles of washing steps, followed by the addition of 100 µM AEBSF and the challenge with the indicated concentrations of ONOO^−^. The percentages of apoptotic cells were determined after 2 h. Untreated cells and cells treated with 25 mM 3-AT served as controls. The results show that PAM efficiently inactivates tumor cell protective catalase, utilizing the same molecular species as shown for direct CAP treatment in Fig. 4b. *Statistical analysis: Apoptosis induction by all concentrations of ONOO*^−^, *after pretreatment with 3-AT or PAM is highly significant (p* < *0*.*001)*. *The effect of all inhibitors*, *except taurine*, *is highly significant (p* < *0*.*001)*.

**Figure 5:**
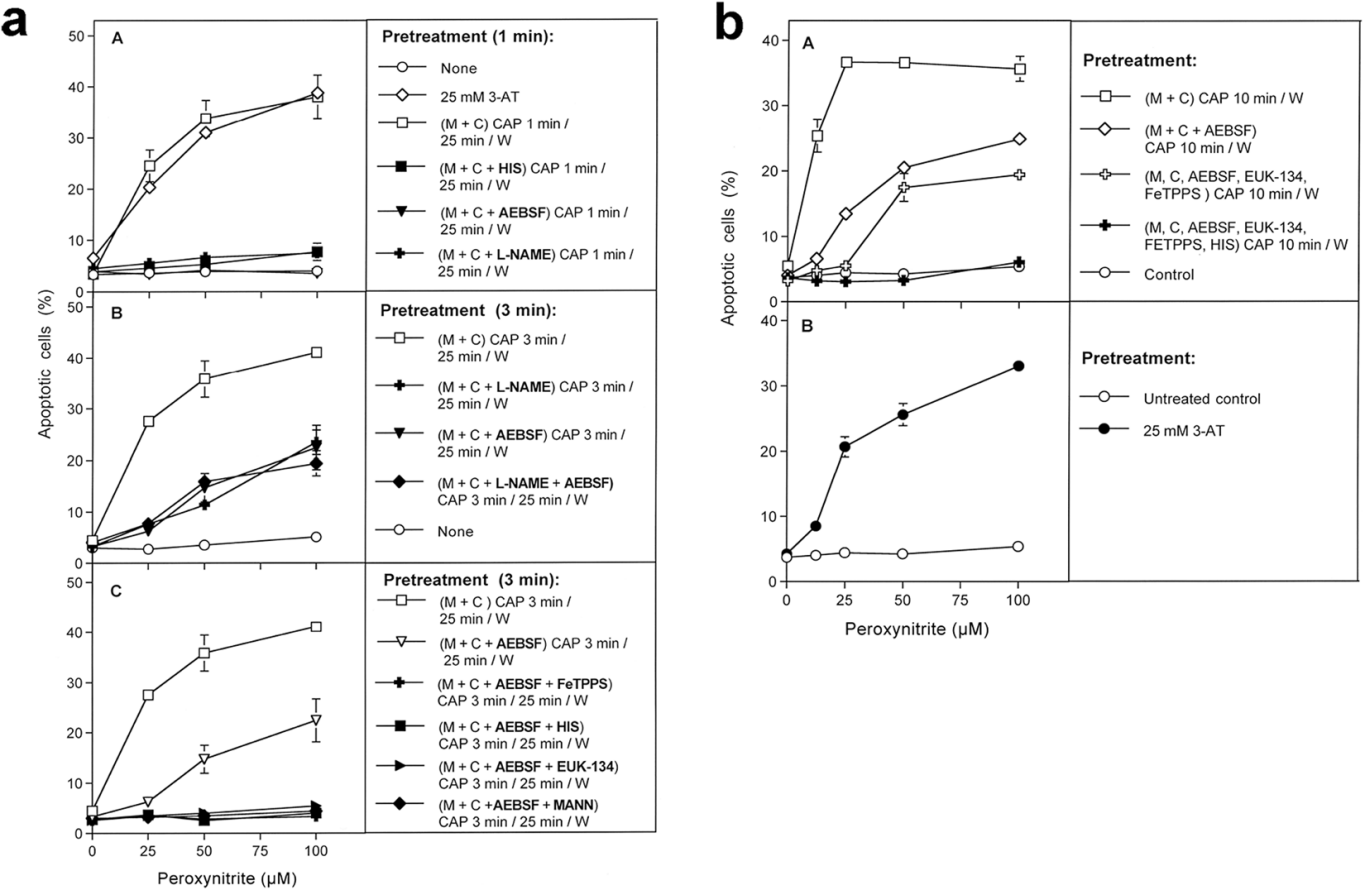
Demonstration of catalase inactivation by primary singlet oxygen (^1^O_2_) derived from long-lived species of CAP-treated medium or from the gaseous phase of CAP. (**a**) Catalase inactivation solely by primary singlet oxygen generated from long-lived species of CAP treated medium requires extending the time of CAP treatment and blocking the generation of secondary singlet oxygen (^1^O_2_). The experiment described in this Figure followed the same principles and used the same concentrations of inhibitors as described before in Fig. 4, but includes a variation of the time of CAP treatment. A confirms that the catalase inhibitor 3-AT as well as pretreatment of tumor cells with CAP for 1 min, followed by 25 min in the same medium allowed for inactivation of tumor cell protective catalase, as determined by a peroxynitrite (ONOO^−^) challenge. Part A also confirms that catalase inactivation is mediated by ^1^O_2_ (inhibition by histidine (HIS)) and that primary ^1^O_2_ is not sufficient for catalase inactivation under these conditions, as inhibition of NOX1 by AEBSF or NOS by L-NAME during pretreatment and incubation completely prevented catalase inactivation through inhibition of secondary singlet oxygen generation. B. When CAP treatment was extended to 3 min, followed by 25 min incubation in the same medium, significant catalase inactivation was determined despite the presence of L-NAME or AEBSF, i. e. even under conditions where secondary ^1^O_2_ generation is inhibited. C. As inactivation of catalase in the presence of AEBSF is inhibited by the parallel presence of either the ^1^O_2_ scavenger histidine (HIS), the ONOO^−^ decomposition catalyst FeTPPS, the catalase mimetic EUK-134 or the ^⋅^OH radical scavenger mannitol (MANN), the underlying effect of primary ^1^O_2_ is confirmed and its generation through the interaction of long-lived species derived from CAP is assured. The action of ^1^O_2_directly derived from CAP is excluded as a significant effective source, as its potential action would not have been inhibited by either FeTPPS, mannitol or EUK-134. Statistical analysis: Apoptosis induction by all concentrations of ONOO^−^after treatment with CAP for 1 or 3 min is highly significant (p < 0.001). The effects of all inhibitors shown under A-C are highly significant (p < 0.001). There is no significant difference between the effects of L-NAME, AEBSF or their combination on apoptosis induction after 3 min CAP treatment and ONOO^−^ challenge. (**b**) Demonstration of the effect of primary singlet oxygen (^1^O_2_) derived directly from the gaseous phase of CAP. The peroxynitrite (ONOO^−^) challenge was used for the determination of catalase inactivation as in the previous experiments. A. Extension of the treatment time of cells in medium (M + C) with CAP to 10 min resulted in massive inactivation of catalase, even if there was no further incubation step after CAP treatment ((M + C) CAP 10 min/W). When the generation of secondary ^1^O_2_ was prevented by the presence of the NOX inhibitor AEBSF (100 µM) during CAP treatment ((M + C + AEBSF) CAP 10 min/W), the degree of catalase inactivation was strongly lowered. Under these conditions, catalase inactivation must be due to primary ^1^O_2_ that may be generated by long-lived species from CAP-treated medium and/or by ^1^O_2_ directly derived from CAP. When the generation of secondary ^1^O_2_was inhibited by AEBSF and the generation of primary ^1^O_2_from long-lived species in CAP-treated medium was blocked through the addition of EUK-134 and FeTPPS, the degree of catalase inactivation was further reduced ((M,C,AEBSF, EUK-134, FeTPPS) CAP 10 min/W). This residual inactivation seemed to be mediated by primary singlet oxygen derived directly from CAP, as its effect was prevented through the presence of histidine in the assays (M,C,AEBSF, EUK-134, FeTPPS, **HIS**) CAP 10 min/W). Statistical analysis: Apoptosis induction after CAP treatment is highly significant for all concentrations of ONOO^−^ (p < 0.001). CAP treatment in the presence of AEBSF caused highly significant apoptosis induction by ONOO^−^ at 25 µM and higher, CAP treatment in the presence of AEBSF, EUK-134 and FeTPPS caused highly significant apoptosis induction at 50 µM ONOO^−^ and higher (p < 0.001).

**Figure 6:**
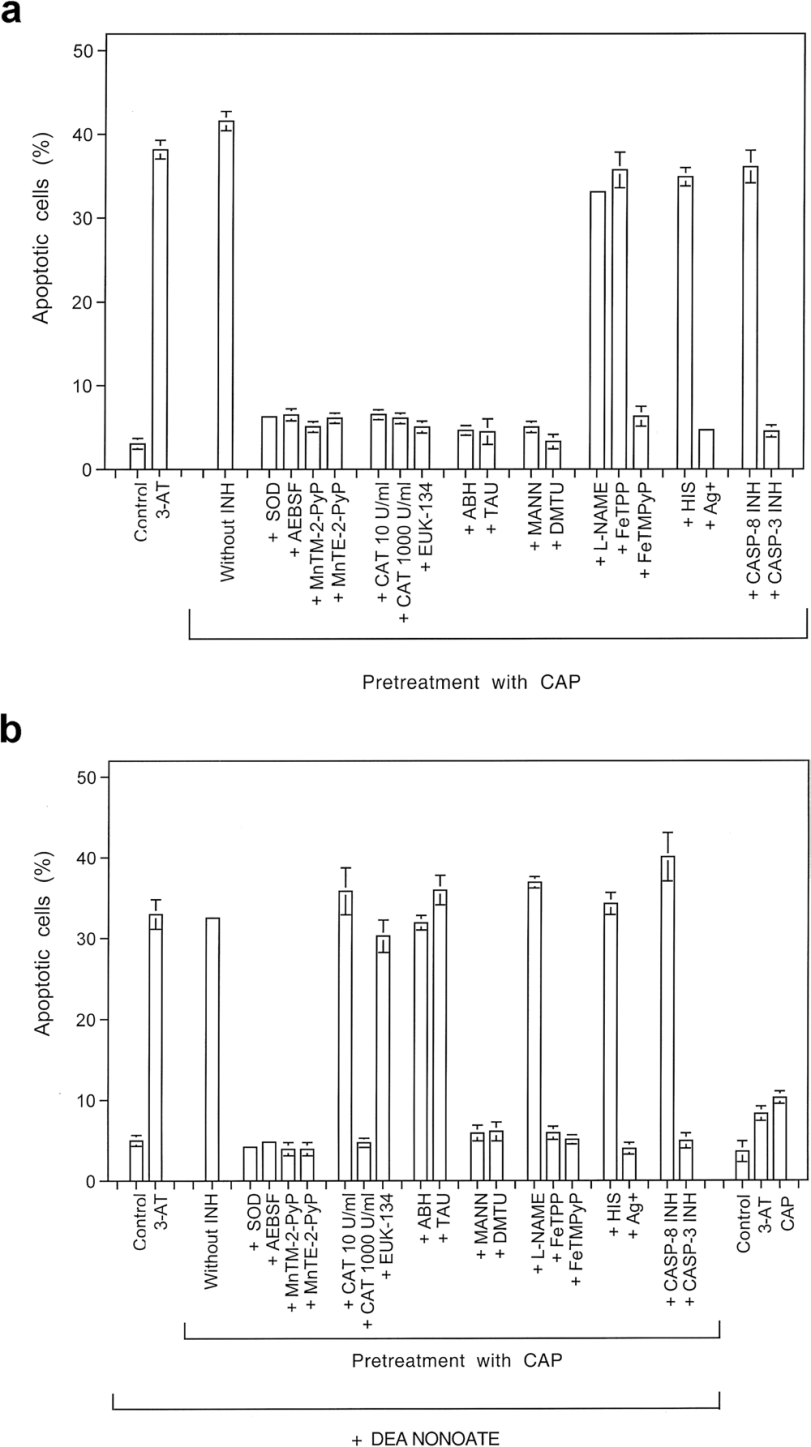
Inactivation of membrane-associated catalase allows for reactivation of intercellular RONS-mediated apoptosis-inducing signaling. (**a**) Catalase inactivation through CAP treatment allows for reactivation of intercellular apoptosis-inducing HOCl signaling. Control assays: MKN-45 cells received 100 mM of the catalase inhibitor 3-AT or remained untreated (“control”) and were then incubated for 3.5 h. CAP pretreatment: MKN-45 cells were pretreated with CAP for 1 min, incubated in the same medium for 25 min and then subjected to three washing cycles. The cells received either no inhibitors or were cultivated in the presence of the indicated inhibitors for 3.5 h, before the percentages of apoptotic cells were determined. SOD and the SOD mimetics MnTM-2-Pyp or MnTE-2-PyP scavenge superoxide anions (O_2_^⋅−^) and generate H_2_O_2_, AEBSF prevents O_2_^⋅−^ generation by NOX1, catalase and the catalase mimetic EUK-134 decompose H_2_O_2_, 4-aminobenzoyl hydrazide (ABH) inhibits peroxidase, taurine (TAU) scavenges HOCl, mannitol (MANN) and dimethyl urea (DMTU) scavenge ^⋅^OH radicals, L-NAME inhibits NOS, FeTPPS selectively decomposes peroxynitrite (ONOO^−^), FeTMPyP decomposes ONOO^−^ and scavenges O_2_^⋅−^, histidine (HIS) scavenges ^1^O_2_, Ag^+^ inhibits aquaporins, caspase inhibitor specifically inhibit caspase-8 or caspase-3. Please find the respective inhibitor concentrations in Table 1. The results show that pretreatment with CAP plus incubation has the same effect as inhibition of catalase with 3-AT. Cell death after catalase inactivation by CAP is mediated by HOCl signaling, depends on aquaporine activity and is executed through caspase-3. It does not require ^⋅^NO/ONOO^−^ signaling, caspase-8 or the action of ^1^O_2_. Statistical analysis: Apoptosis induction after CAP treatment, as well as inhibition by all inhibitors except L-NAME, FeTPPS, histidine and caspase-8 inhibitor was highly significant (p < 0.001). (**b**). The effect of the ^⋅^NO donor DEA NONOate on intercellular apoptosis-inducing signaling after catalase inactivation by CAP treatment. Assays without pretreatment (“control”), treated with 25 mM of the catalase inhibitor 3-AT or with CAP for 1 min, followed by 25 min incubation, three cycles of washing and addition of the indicated inhibitors received 0.5 mM of the ^⋅^NO donor DEA NONOate. Parallel controls without pretreatment or treated with 3-AT or CAP remained free of DEA NONOate (Controls of the right side of the panel). The percentages of apoptotic cells were determined 2 h after addition of the ^⋅^NO donor. The results show that the addition of the ^⋅^NO donor had shifted apoptosis inducing signaling by cells with inactivated catalase to ^⋅^NO/ONOO^−^ signaling, as inhibitors that specifically address HOCl signaling (like the peroxidase inhibitor ABH and the HOCl scavenger taurine (TAU)) were now effective, whereas cell death remained dependent on superoxide anions (O_2_^−^) and ^⋅^OH radicals. Inhibition by FeTPPS and the requirement for high concentrations of catalase for inhibition is indicative for ^⋅^NO/ONOO^−^ signaling. The NOS inhibitor L-NAME remained without effect, as DEA NONOate was the dominating source for ^⋅^NO. *Statistical analysis: Apoptosis induction after pretreatment with CAP and addition of DEA NONOate*, *as well as inhibition by all inhibitors except 10 U/ml catalase*, *EUK-134*, *ABH*, *taurine*, *L-NAME*, *histidine and caspase-8 inhibitor was highly significant (p* < *0*.*001)*.

This procedure was also successfully applied to the study of PAM action (regime B, Fig. 1(b)) (Results shown in Fig. 3a). Regime C in Fig. 1(b) describes a kinetic analysis of apoptosis induction after CAP treatment, in which the singlet oxygen scavenger histidine was present either during CAP treatment plus the subsequent incubation step for 25 min, or thereafter (Fig. 3(b)). This approach allowed to differentiate between an early, ^1^O_2_ – dependent step and a subsequent ^1^O_2_ – independent step. This approach was also applied to PAM treated cells (not explicitely shown in Fig. 1(b). The kinetic analysis of CAP- and PAM-mediated apoptosis induction will be shown in Fig. 3b. Based on this information, it was possible to study in detail the initial step of CAP (and PAM) action through a ONOO^−^ challenge, that allows to monitor the inactivation of membrane-associated catalase^12^ (regime D in Fig. 1(b)). The addition of various inhibitors/scavengers during the short treatment with CAP or PAM allowed to define the molecular species involved in this step. Results will be shown in Figs 4 and 5. Finally, the regime described under E in Fig. 1(b) was based on an initial treatment of cells with CAP, which was followed by an incubation in the absence or presence of various inhibitors. This experiment allowed to define the molecular species involved in intercellular apoptosis-inducing RONS signaling after CAP-mediated inactivation of catalase. These results will be shown in Fig. 6. Further experimental approaches, not included in this scheme, were studying the role of aquaporins and intracellular glutathione and the role of proton pumps. Finally, the window of CAP and PAM doses for selective action towards tumor cells was defined.

The experiments in the main part of this manuscript were performed with a CAP source operating in ambient air in the streamer corona regime. Supplementary information shows central data obtained through the application of a CAP source operation in the transient spark regime.

## Results

### Apoptosis induction by CAP and PAM

Cold atmospheric plasma, applied in the streamer corona regime, caused apoptosis induction in human MKN-45 gastric carcinoma cells (Fig. 2(a):A–D). Two hours after treatment, differences in CAP exposure time of 20 seconds to 1 minute resulted in slight, but significant differences in the apoptotic response, whereas at five hours past treatment, differences in the dose responses were only minor. Apoptosis induction in the tumor cells by CAP seemed to be mediated by a relatively fast singlet oxygen (^1^O_2_)-dependent process, as the presence of the ^1^O_2_ scavenger histidine (HIS) during the treatment completely prevented apoptosis induction, whereas the addition of HIS 20 minutes after CAP treatment only caused marginal inhibitory effects. The strong inhibitory effect of HIS added at two minutes after CAP treatment, demonstrates that ^1^O_2_ directly derived from CAP cannot be the major responsible agent, as ^1^O_2_ has an extremely short life time in the range of microseconds. This finding rather shows i) that CAP-treated medium generates ^1^O_2_ over a period of several to tens of minutes, and that the concentration of ^1^O_2_ generated during this time is actually required to trigger the observed biological effect.

Treatment of medium with CAP in the absence of cells, followed by subsequent transfer of this medium to tumor cells (i.e. formation of PAM), caused apoptosis induction in the same range of effectivity as direct treatment of cells by CAP (Fig. 2(a):E,F). This effect of “plasma-activated medium (PAM)” was also completely inhibited by the ^1^O_2_ scavenger histidine (HIS).

CAP-mediated apoptosis induction was dependent on CAP treatment time and on the density of the target cells, as shown in Supplementary Figs 1 and 2. The concentration response curve was characterized by a steep increase of the apoptotic response at low CAP treatment times, followed by a long-lasting plateau. CAP-dependent apoptosis induction was abrogated by the presence of ^1^O_2_ inhibitor HIS at all doses applied.

CAP-mediated apoptosis induction was dependent on an early and short-lasting singlet oxygen- and ONOO^−^-dependent step, which seemed to be completed within 20 minutes after CAP treatment (Fig. 2(b):A,B). In contrast, inhibition of NOX1 activity by AEBSF and inhibition of aquaporins through Ag^+^ prevented apoptosis even if the inhibitors had been added at a time point when the ^1^O_2_ and ONOO^−^-dependent step had already been completed (Fig. 2(b):C,D).

Apoptosis was induced in MKN-45 cells dependent on the concentration of PAM (Fig. 3(a)). A strong leftward shift of the concentration-response curve of PAM was observed with time (Fig. 3(a):AB versus Fig. 3(a):CD). This indicates substantial kinetic differences for apoptosis induction at higher and lower concentrations of PAM. Similar to CAP action, the effect of PAM was also characterized by a fast ^1^O_2_- and ONOO^−^-dependent step. This step seemed to be followed by a process that depended on (O_2_^−^), peroxidase and HOCl. In line with previous findings in model experiments performed with long-lived compounds from CAP and PAM, this is indicative of reactivation of intercellular HOCl-dependent apoptosis-inducing signaling^56^. Reactivation of HOCl signaling by tumor cells has been shown to be dependent on substantial inactivation of membrane-associated catalase^8,9,12^.

An initial treatment with CAP (Fig. 3(b):A) caused similar kinetics of apoptosis induction in tumor cells as PAM (Fig. 3(b):B)). Both treatments were confirmed to be strictly dependent on ^1^O_2_ in their initial step, whereas apoptosis induction following initial treatment with CAP or PAM was independent of ^1^O_2_.

### Dissection of the experimental system

#### Treatment with CAP or PAM leads to the inactivation of membrane-associated catalase of tumor cells

In analogy to the results obtained for reconstitution experiments^56^ with defined concentrations of the long-lived species H_2_O_2_ and nitrite (which are the essential components of PAM), and in line with established concepts on reactivation of intercellular ROS-dependent apoptosis induction^7–9,15,52^, CAP- or PAM-mediated inactivation of membrane-associated catalase seemed to be an attractive and conclusive hypothesis to explain the initial steps of action.

Inactivation of membrane-associated catalase can be specifically tested through a challenge with exogenous peroxynitrite (ONOO^−^)^12^, as intact membrane-associated catalase decomposes ONOO^−^, whereas inactivation of membrane-associated catalase allows apoptosis induction by exogenous ONOO^−^. This requires formation of peroxynitrous acid (ONOOH) and its decomposition into ^⋅^NO_2_ and apoptosis-inducing ^⋅^OH radicals.This process is favoured in close vicinity to the cell membrane, due to the activity of proton pumps. Intracellular catalase has no impact on this process, as it cannot reach extracellular ONOO^−^. Passage of ONOO^−^ through the membrane would cause lipid peroxidation before the intracellular catalase might interact with ONOO^−^.

As shown in Fig. 4(a), tumor cells that had not been pretreated were protected towards the applied concentrations of ONOO^−^. Treatment of tumor cells with CAP, followed by 25 min incubation in the same medium, seemed to cause the same degree of inactivation of membrane-associated catalase as the incubation with the established catalase inhibitor 3-AT, as both treatment regimes caused similar apoptotic responses to the dose-dependent ONOO^−^ challenge.

Treatment of tumor cells with CAP for one minute without the subsequent incubation period was not sufficient for the inactivation of catalase. Washing of the tumor cells immediately after CAP treatment and further incubation in fresh medium resulted in a negligible catalase inactivation. This finding shows that the presence and action of long-lived species from CAP is necessary to achieve catalase inactivation during the 25 min incubation step. The presence of the NOX1 inhibitor AEBSF during CAP treatment and/or the subsequent incubation for 25 min in the original medium, prevented the inactivation of catalase. This finding demonstrates that, following CAP treatment, the cells must have contributed to the inactivation of their catalase through NOX1-derived O_2_^⋅−^. As shown in Fig. 4(b), catalase inactivation by CAP under standard conditions required ^1^O_2_, NOX1-derived O_2_^⋅−^, H_2_O_2_, NOS-derived ^⋅^NO, ONOO^−^, ^⋅^OH radicals and the activity of caspase-8, as seen from the inhibitor profile of apoptosis induction by the ONOO^−^- challenge. Inactivation of catalase did not require the action of HOCl, as taurine had no inhibitory effect on catalase inactivation through CAP.

Inactivation of membrane-associated catalase by PAM seemed to be mediated by the same molecular players as the catalase inactivation by CAP (Fig. 4(c)). Again, PAM seemed to trigger a strong autoamplificatory secondary singlet oxygen generation by the tumor cells, as seen by the strong inhibitory effect of the NOX1 inhibitor AEBSF.

Figure 5(a):A confirms that 1 min treatment with CAP followed by 25 min incubation of the cells in the same medium did not cause detectable catalase inactivation when secondary ^1^O_2_ generation had been prevented by either inhibiting NOX1 or NOS; by AEBSF or L-NAME, respectively. When, however, CAP treatment had been extended to 3 min, inactivation of catalase despite the presence of AEBSF or L-NAME was demonstrated (Fig. 5(a):B). The degree of inactivation under these conditions was lower than in the absence of AEBSF or L-NAME. Inactivation of catalase in the presence of AEBSF seemed to be due to primary singlet oxygen generated by the long-lived species in plasma-treated medium, as it was prevented through scavenging of ^1^O_2_, ONOO^−^, H_2_O_2_ and ^⋅^OH radicals by histidine, FeTPPS, EUK-134 and mannitol (Fig. 5(a):C).

Further extension of the treatment time with CAP to 10 min (without subsequent incubation step) demonstrated catalase inactivation by CAP-derived ^1^O_2_. However, this approach required a prior application of the NOX1 inhibitor AEBSF in combination with EUK-134 and FeTPPS, which catalytically decompose H_2_O_2_ and ONOO^−^, respectively (Fig. 5(b)). These conditions prevent formation of primary ^1^O_2_ generation from long-lived species in PAM and the generation of secondary ^1^O_2_ by the cells, and thus allow to focus on the effects induced by singlet oxygen derived directly by CAP.

We note that it is possible that CAP can create ^1^O_2_ in the gas phase and that some of these species could enter the top parts of the cell culture medium before being lost. Demonstration of the possible action of singlet oxygen generated in this direct fashion by CAP was only possible in cell cultures that were partially in suspension, like MKN-45 cells, but not for SIHA or SKN-MC cells that were firmly attached to the bottom of the tissue culture plate and covered by medium.

#### Catalase inactivation allows for subsequent intercellular ROS/RNS signaling

Treatment of MKN 45 tumor cells with CAP for 1 min, followed by 25 minutes incubation and a washing step, allowed subsequent apoptosis induction to a similar degree as the direct inhibition of catalase by 3-AT (Fig. 6(a)). Apoptosis induction after CAP treatment was blocked when O_2_^⋅−^ synthesis was inhibited by AEBSF, when O_2_^⋅−^ was scavenged by SOD or the SOD mimetics MnTM-2-Pyp or MnTE-2PyP, when H_2_O_2_ was decomposed by catalase or the catalase mimetic EUK-134. Strong apoptosis inhibition occurred also when peroxidase was blocked by ABH, when HOCl was scavenged by taurine and when ^⋅^OH radicals were scavenged by mannitol or DMTU. These findings are in perfect alignment with the inhibitor profile of the HOCl signaling pathway. The ^⋅^NO/ONOO^−^ signaling pathway did not seem to play a role for apoptosis- inducing signaling under these conditions, as inhibition of NOS by L-NAME, or decomposition of ONOO^−^ by FeTPPS had no inhibitory effect on apoptosis induction. ^1^O_2_ and caspase-8 played no role for apoptosis induction, wherease the aquaporin inhibitor Ag^+^ and caspase-3 caused a strong inhibition of apoptosis.

When the fast decaying ^⋅^NO donor DEA NONOate was added to CAP-treated cells, apoptosis-inducing signaling was completely shifted to ^⋅^NO/ONOO^−^ signaling at the expense of HOCl signaling (Fig. 6(b)).

Abrogation of apoptosis-inducing HOCl as well as ^⋅^NO/ONOO^−^ signaling by exogenous soluble catalase was in line with the proposed dominant controling function of catalase for these processes. Importantly and in line with previous findings^9^. Inhibition of ^⋅^NO/ONOO^−^ signaling required higher concentrations of exogenous catalase than inhibition of HOCl signaling.

#### The relevance of catalase inactivation for apoptosis-inducing RONS signaling

**Figure 7:**
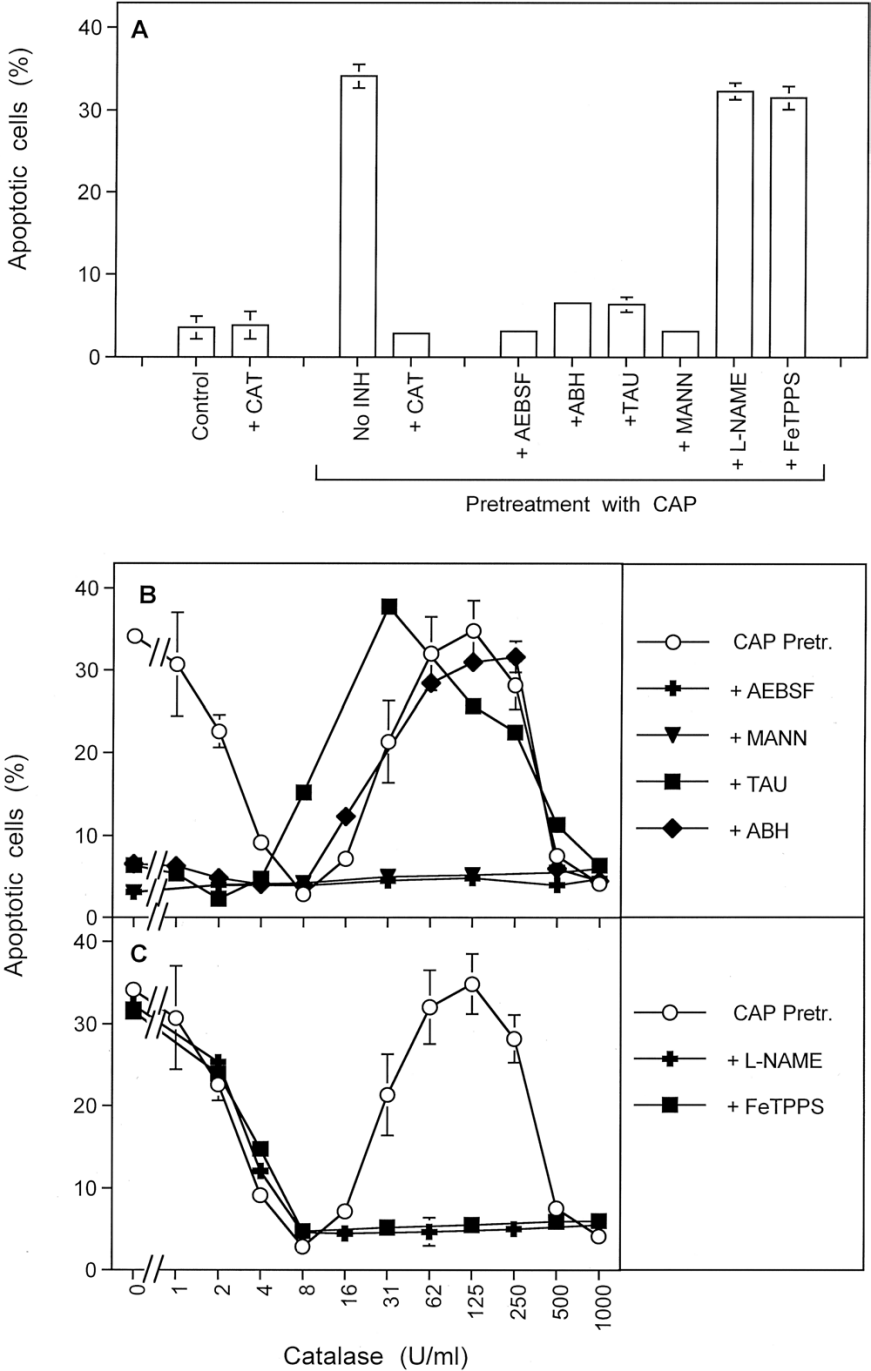
The relevance of catalase inactivation for apoptosis-inducing ROS/RNS signaling. (**A**) MKN-45 cells remained without pretreatment (control), received 8 U/ml exogenous catalase or were treated with CAP for 1 min, followed by 25 min of incubation and three cycles of washing. CAP-pretreated cells then remained either without inhibitors, or received 8 U/ml catalase, 100 µM of the NOX1 inhibitor AEBSF, 150 µM of the peroxidase inhibitor ABH, 50 mM of the HOCl scavenger taurine (TAU) or 25 µM of the peroxynitrite (ONOO^−^) decomposition catalyst FeTPPS. The determination of apoptotic cells after 3.5 h showed that apoptosis induction required treatment with CAP. The effect of CAP treatment was due to selective establishment of HOCl signaling and was completely inhibited by exogenous catalase. This allows to conclude on the functional role of catalase for the protection of tumor cells towards HOCl signaling. This is in line with catalase being the central target for CAP treatment. (**B**,**C**) MKN-45 cells were pretreated with CAP for 1 min, followed by 25 min incubation in the same medium and three cycles of washing steps. The cells remained without inhibitor or received the indicated inhibitors. All assays then received the indicated increasing concentrations of catalase and apoptosis induction was determined after 3.5 h. The result shows that CAP treatment reactivated specifically HOCl signaling, without initial contribution of ^⋅^NO/ONOO^−^ signaling. With increasing concentrations of catalase, first HOCl signaling was efficiently inhibited before ^⋅^NO/ONOO^−^ signaling was reactivated. Finally ^⋅^NO/ONOO^−^signaling was inhibited by very high concentrations of exogenous catalase. Statistical analysis: A: Apoptosis induction after CAP treatment, as well as inhibition by all inhibitors except FeTPPS was highly significant (p < 0.001). B, C: Apoposis induction after CAP treatment and in the presence of 0–2, as well as 31–250 U/ml catalase was highly significant (p < 0.001). The inhibition by AEBSF and mannitol was highly significant at all catalase concentrations (p < 0.001). Inhibition by taurine and ABH in the low concentration range of catalase (0–2 U/ml), and by L-NAME and FeTPPS in the high concentration range (31–250 U/ml) was highly significant (p < 0.001). The shifting effect of taurine in the concentration range of 16 and 31 U/ml was highly significant (p < 0.001).

As shown in Fig. 7A, apoptosis induction by HOCl signaling, in the absence of ^⋅^NO/ONOO^−^ signaling, was confirmed for CAP-treated tumor cells. As HOCl signaling was completely prevented in the presence of 8 U/ml exogenous catalase, the functional role of the targeted membrane-associated catalase of tumor cells for their protection was confirmed. Gradually increasing concentrations of exogenous catalase caused gradually decreased apoptosis induction in the CAP-treated tumor cells (Fig. 7B). Cell death was completely prevented at a concentration of 8 U/ml catalase. Further addition of exogenous catalase seemed to allow reactivation of ^⋅^NO/ONOO^−^ signaling, in the absence of HOCl signaling, as deduced from the inhibitor profile (Fig. 7B,C). Finally, apoptosis was completely blocked at 1000 U/ml of exogenous catalase. These findings again demonstrate the relevance and central role of catalase inactivation for cell death-inducing ROS/RNS signaling of tumor cells. They also show a differential requirement of catalase concentrations for the inhibition of the two signalling pathways. In addition, they confirm the established negative interference of HOCl signaling towards ^⋅^NO/ONOO^−^ signaling. Furthermore, adding the ^⋅^NO donor DEA NONOate, resulting in the formation of large amounts of ONOO^−^, showed similar negative interference of ^⋅^NO/ONOO^−^ signaling towards HOCl signaling.

#### SiRNA-based analysis of the molecular players involved in CAP-mediated apoptosis induction

Small interfering RNA (SiRNA)-mediated knockdown of defined proteins has been instrumental for the elucidation of ROS/RNS-mediated signaling and its control^4,13^. This instrument was therefore utilized for the analysis of CAP-mediated effects on tumor cells. However, in contrast to inhibitors that can be applied differentially, siRNA-mediated knockdown cannot determine per se at which step an enzyme is involved.

Supplementary Figure 3 shows that CAP-mediated, singlet oxygen- and ROS-signaling-mediated apoptosis induction in tumor cells was dependent on NOX1, but not on NOX-3, -4, -5. Apoptosis induction seemed to require expression of DUOX and iNOS, but not of nNOS. The signaling relevant molecules TGFbeta, its receptor as well as PKC zeta were also found to be essential. As recently shown, these compounds are essential for NOX1 activity^4^. The FAS receptor and caspase-8 seemed to be required for enhancement of defined signaling processes (as deduced in parallel by inhibitor studies), whereas the requirement for SMASe, Bak, Diablo, VDAC, CYTC, APAF, Caspase-9 and Caspase-3 was indicative for the execution of the mitochondrial pathway of apoptosis after CAP treatment.

Further information on the signaling relevant function of caspase-8 is presented in Supplementary Fig. 4.

#### CAP-mediated inactivation of SOD

Singlet oxygen can inactivate SOD as well as catalase^62,63^. Therefore, an impact of CAP and PAM treatment on the SOD activity on the surface of tumor cells was also expected. Inactivation of membrane-associated SOD leads to an increase in free extracellular superoxide anions that can be verified by an increased reaction with exogenous apoptosis-inducing HOCl. As shown in Supplementary Fig. 5A, CAP treatment seemed to cause a marked increase in free superoxide anions as detected by a leftward shift of the concentration-dependency of apoptosis induction by HOCl. This effect was analogous and in the same range of efficiency as the inactivation of SOD by neutralizing single domain antibodies (Supplementary Fig. 5B). Control assays confirmed that single domain antibodies that bound to SOD without neutralizing its activity did not cause an analogous effect.

#### The role of aquaporins for CAP-mediated apoptosis induction in tumor cells

**Figure 8:**
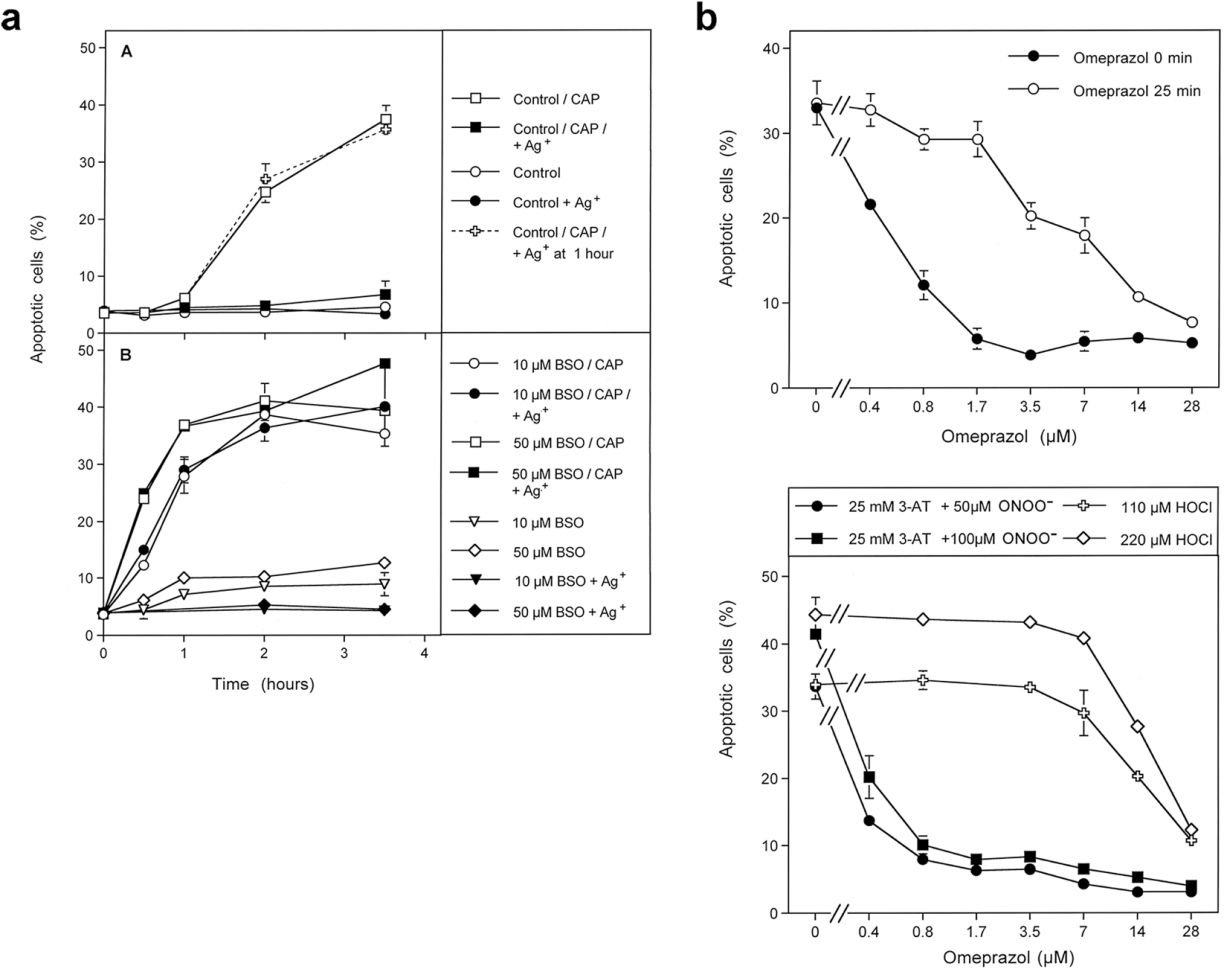
The role of aquaporins and proton pumps for CAP-mediated apoptosis induction. (**a**) The role of aquaporins for intercellular apoptosis-inducing signaling after CAP treatment. Tumor cells were pretreated in the absence of inhibitors (Control) or in the presence of 10 µM or 50 µM of buthionine sulfoximine (BSO), an inhibitor for glutathione synthesis, for 14 h. The cells were washed and either received no further treatment, addition of 5 µM of the aquaporin inhibitor Ag^+^, treatment with CAP for 1 min plus 25 min incubation or CAP treatment plus Ag^+^. The percentages of apoptotic cells were determined kinetically. Part A shows that control cells required a 1 hour lag phase before they responded to CAP treatment with apoptosis induction. CAP-mediated apoptosis induction was completely blocked when Ag^+^ had been added at 0 min of incubation, whereas there was no inhibitory effect when Ag^+^ had been added 1 hour after the beginning of the incubation. Part B shows that pretreatment of the cells with BSO, i. e. inhibition and consumption of GSH, allowed for a faster onset of CAP-mediated apoptosis induction that was no longer inhibited by Ag^+^. Statistical analysis: A: Apoptosis induction after CAP treatment at 2 h and later, as well as inhibition by Ag^+^ added before CAP treatment was highly significant (p < 0.001), whereas addition of Ag^+^ 1 h after CAP treatment was without significant inhibitory effect. B: Apoptosis induction after pretreatment with BSO, followed by CAP treatment was highly significant (p < 0.001), whereas Ag^+^ did not cause significant inhibition. (**b**) The role of proton pumps for CAP-mediated apoptosis induction. Upper graph: The proton pump inhibitor omeprazol was added at increasing concentrations to MKN-45 cells either at the beginning of CAP treatment for 1 min or 25 min after CAP treatment. Apoptosis induction was determined after 3.5 h. The result shows that later effects of CAP treatment are less sensitive to the proton pump inhibitor than earlier ones. Lower graph. Model experiments with omeprazol using defined signaling pathways for apoptosis induction. Increasing concentrations of omeprazol were added to MKN-45 cells that were either treated with the indicated concentrations of the catalase inhibitor 3-AT plus peroxynitrite (ONOO^−^) or HOCl. The percentages of apoptotic cells were determined after 2 h. The result shows that ONOO^−^-mediated apoptosis induction was more efficiently inhibited by omeprazol than HOCl-dependent apoptosis induction. Statistical analysis: Upper graph: When omeprazol had been added at 0 min, the inhibitor caused highly significant inhibition at 0.4 µM and higher concentrations, whereas it caused highly significant inhibition at 3.5 µM and higher when added at 25 min (p < 0.001). Lower graph: Inhibition of ONOO^−^-mediated apoptosis induction was highly significant (p < 0.001) at 0.4 µM omeprazol and higher, whereas highly significant inhibition of HOCl-mediated apoptosis induction required concentrations of 14 µM or higher.

Apoptosis induction after CAP treatment was completely prevented in the presence of the aquaporine inhibitor Ag^+^ (Fig. 8(a)). However, when the intracellular glutathione level had been lowered through preincubation of the tumor cells with buthionine sulfoximine (BSO), an inhibitor of glutathione synthesis, the kinetics of apoptosis induction after CAP treatment showed no lag phase and was faster. Importantly, apoptosis induction under these conditions was no longer blocked by Ag^+^. These data show that the action of aquaporins is not required when the glutathione level is lowered by biochemical treatment before application of CAP.

#### The role of proton pumps for CAP mediated apoptosis induction

The formation of peroxynitrous acid (ONOOH), with its high potential to spontaneously dissociate into ^⋅^NO_2_ and ^⋅^OH radicals, is essential for the generation of secondary ^1^O_2_^15,77^. The formation of ONOOH acid is favoured at the membrane of tumor cells through proton pumps, whereas ONOO^−^ distant from cell membranes has a higher chance to react with CO_2_ and be unavailable for activity near the cell membrane^78–80^. As shown in Fig. 8(b), inhibition of proton pumps by omeprazol had a very strong inhibitory effect on the early steps of CAP-mediated apoptosis induction, whereas omeprazol inhibited the following steps to a much lesser degree. Recall that acidification from proton pumps in buffered cell medium is necessary for CAP-generated H_2_O_2_ and nitrite to form primary ^1^O_2_. This pattern is in good agreement with the differential inhibition of ONOO^−^- and HOCl-dependent apoptosis induction by the proton pump inhibitor.

#### Differential effects of CAP and PAM on nonmalignant cells and tumor cells: clues to establishment of selective antitumor effects

In addition to apoptosis induction in MKN-45 human gastric carcinoma cells, CAP treatment of 1 min was also sufficient to substantially induce apoptosis in human neuroblastoma cells (SHEP), Ewing sarcoma cells (SKN-MC) and cervix carcinoma cells (SIHA) (Supplementary Fig. 6). Apoptosis induction in these cells seemed to depend on NOX1-derived O_2_^⋅−^, as it was inhibited by AEBSF. It was also dependent on ^1^O_2_, as seen by the efficient inhibition by the ^1^O_2_ scavenger histidine. The strong inhibitory effect of histidine that had been added one minute after CAP treatment indicated an action of ^1^O_2_ that was not generated in the gas phase but generated by CAP-generated nitrite and H_2_O_2_ in medium. The singlet oxygen-dependent step seemed to be completed 30–60 min after CAP treatment.

**Figure 9:**
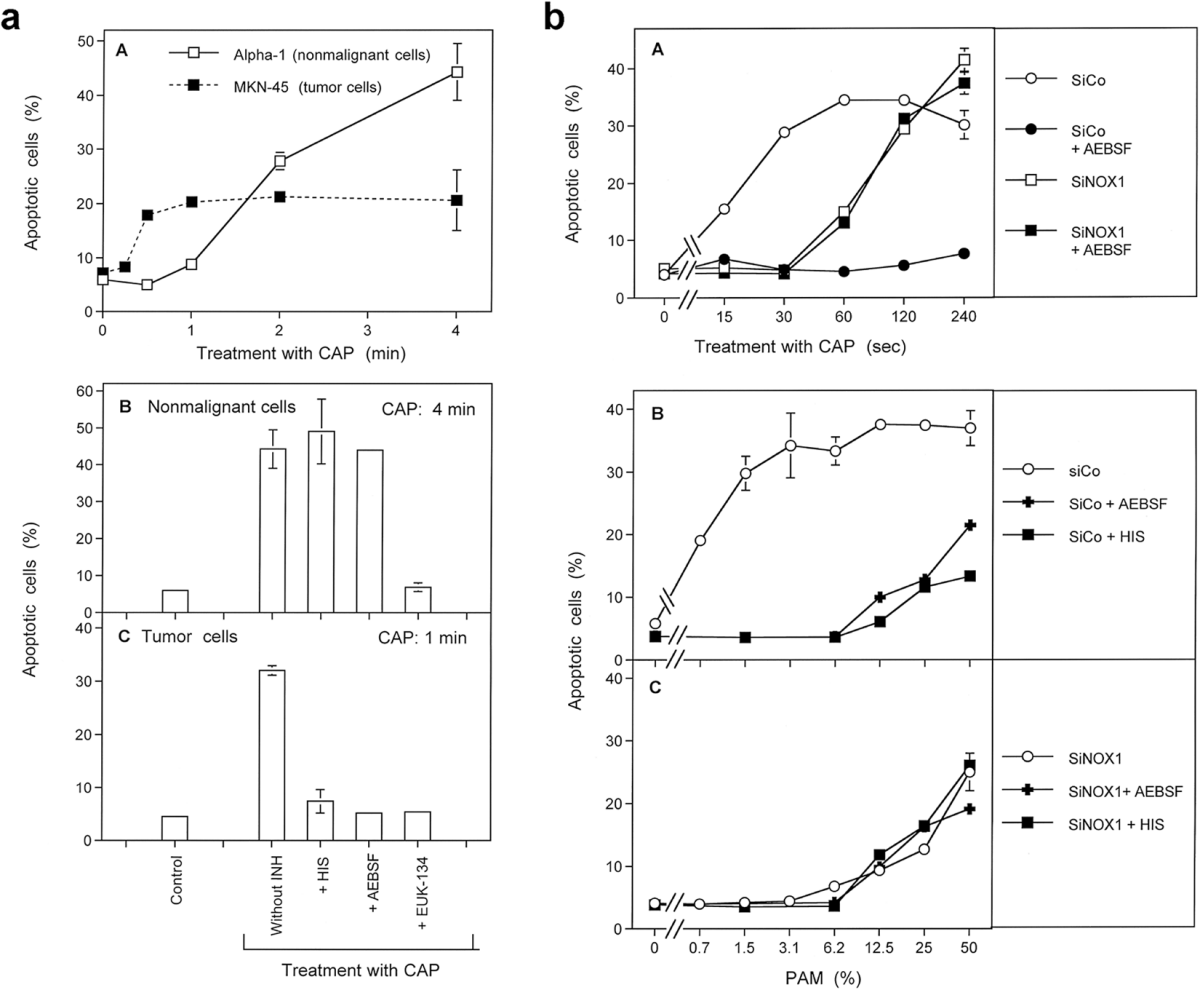
Differential effect of CAP on nonmalignant cells and tumor cells. (**a**) Defining the dose of selective action of CAP towards tumor cells and the underlying chemical biology. A. Nonmalignant diploid human fibroblasts (Alpha-1) and human gastric carcinoma cells (MKN-45) were treated with CAP for the indicated times. The percentages of apoptotic cells were determined after 4.5 h. The figure shows that CAP treatment up to 1 min caused apoptosis induction in tumor cells but not in nonmalignant cells. At higher times of treatment, nonmalignant cells showed a stronger response than tumor cells. This can be explained by the absence of catalase on the membrane of nonmalignant cells. B,C. Nonmalignant cells (Alpha-1) and tumor cells (MKN-45) remained either untreated (control) or were treated with CAP for 4 or 1 min, respectively, in the absence or presence of either 50 mM of the singlet oxygen (^1^O_2_) scavenger histidine (HIS), or 100 µM of the NOX1 inhibitor AEBSF or 20 µM of the catalase mimetic EUK-134. The percentages of apoptotic cells were determined after 4.5 h. The results show that CAP-mediated apoptosis induction in nonmalignant cells seems to be directly dependent on H_2_O_2_, and is independent of ^1^O_2_ or NOX-derived superoxide anions (O_2_^⋅−^). In contrast, CAP-mediated apoptosis induction in tumor cells is dependent on H_2_O_2_, ^1^O_2_ and NOX-derived O_2_^⋅−^). Statistical analysis: A: Apoptosis induction in the tumor cells was highly significant after CAP treatment of 0.5 min, whereas highly significant apoptosis induction in nonmalignant cells required 2 min (p < 0.001). B: Apoptosis induction in nonmalignant cells was highly significant after CAP treatment (p < 0.001). Inhibition of apoptosis induction by EUK-134 was highly significant (p < 0.001), whereas there was no inhibition by histidine or AEBSF. C: Apoptosis induction in tumor cells, as well as inhibition by all three inhibitors was highly significant (p < 0.001). (**b**) Biochemical basis for selective action of CAP and PAM towards malignant cells. MKN-45 cells were treated with either irrelevant control siRNA (“SiCo”) or siRNA directed towards NOX1 (“siNOX1”) for 24 h. This causes knockdown of NOX1 activity to less than 5% residual activity. As the expression of membrane-associated catalase is controlled by NOX1-derived superoxide anions (O_2_^⋅−^) and their dismutation product H_2_O_2_, siNOX1 cells are no longer protected by membrane-associated catalase^12^. Therefore, siCo cells show the phenotype of tumor cells, whereas siNOX1 cells resemble nonmalignant cells with respect to the redox biology at their membrane. A. SiCo and SiNOX1 cells were treated with CAP for increasing time, in the absence or presence of the NOX1 inhibitor AEBSF. The percentages of apoptotic cells were determined after 4 h. The results show that siNOX cells were reacting more sensitive to CAP treatment than siCo cells. Whereas the response of siCo cells depended on NOX1, the reaction of siNOX1 cells was not sensitive to the NOX inhibitor. Taken together, CAP treatment up to 1 min defines a window in which selective apoptosis induction in tumor cells can be achieved, whereas higher treatment times do not allow differential apoptosis induction in malignant and nonmalignant cells. B,C. SiCo and SiNOX1 cells were treated with increasing concentrations of PAM (generated through 1 min treatment of medium with CAP), in the absence and presence of the NOX1 inhibitor AEBSF or the ^1^O_2_ scavenger histidine (HIS). The percentages of apoptotic cells were determined after 4 h. The results define a window of selectivity of PAM action towards tumor cells compared to nonmalignant controls. Selective apoptosis induction in tumor cells was dependent on ^1^O_2_ and NOX1-derived O_2_^⋅−^, whereas apoptosis induction in the nonmalignant controls was independent on ^1^O_2_ and O_2_^⋅−^. Statistical analysis: A. Treatment of SiCo cells with CAP resulted in highly significant apoptosis induction after 15 sec of treatment, whereas treatment of siNOX1 cells required 60 min (p < 0.001). Whereas inhibition of apoptosis induction by AEBSF in siCo cells was highly significant (p < 0.001), there was no significant inhibition by AEBSF in siNOX1 cells. B: Apoptosis induction in siCo cells by PAM, as well as its inhibition by AEBSF and histidine was highly significant at all concentrations of PAM (p < 0.001). Apoptosis induction in siNOX1 cells by PAM was highly significant at concentrations of 25% PAM and higher (p < 0.001). There was no significant inhibition by AEBSF or histidine.

Apoptosis induction in nonmalignant diploid human fibroblasts by CAP required longer than 1 min treatment and its effects increased with exposure time (Fig. 9(a):A). Apoptosis induction in nonmalignant cells by high doses of CAP seemed to be mediated primarily by H_2_O_2_, independent of singlet oxygen and NOX-derived superoxide anions (Fig. 9(a):B). This is contrasted by the strict dependence of CAP-mediated apoptosis induction in tumor cells on singlet oxygen, NOX-derived superoxide anions and H_2_O_2_ (Fig. 9(a):C). As noted previously, H_2_O_2_ is involved in CAP-mediated apoptosis induction in tumor cells through its role during the formation singlet oxygen, intracellular glutathione depletion and substrate for peroxidase to generate HOCl.

Sustained NOX1 activity and expression of membrane-associated catalase by tumor cells are the most remarkable redox-related differences between tumor cells and nonmalignant cells^5–9,12,18,52^. As the expression of membrane-associated catalase of tumor cells is modulated by tumor cell-derived superoxide anions and H_2_O_2_, siRNA-mediated knockdown of NOX1 causes strong downmodulation of catalase expression^12^. As a result, tumor cells treated with siRNA directed towards NOX1 show the phenotype of nonmalignant cells with respect to their intercellular redox biology, i. e. they do not generate extracellular O_2_^⋅−^ and do not express membrane-associated catalase. Otherwise, they are genetically identical to the parental cells. This experimental approach therefore allows a comparison between redox chemistry-related reactions of nonmalignant and tumor cells. As shown in Fig. 9(b), treatment with CAP up to 30–60 sec caused selective apoptosis induction in control tumor cells (siCo), due to their activity of NOX1. The dependence on NOX1 is seen from the inhibition by the NOX1 inhibitor AEBSF. At higher doses of CAP, the effect on control tumor cells (siCo) remained dependent on NOX1, but cells with the knockdown of NOX1 (siNOX1) and subsequent downmodulation of catalase, also showed apoptosis induction. The effect on these cells with their nonmalignant phenotype (NOX1 negative/catalase negative) was proven to be completely independent of NOX1, as it was not inhibited by AEBSF.

Up to 12.5% PAM, apoptosis was selectively induced in control tumor cells (siCo) and was completely dependent on NOX1-derived O_2_^⋅−^ and ^1^O_2_, as it was inhibited by AEBSF and histidine. (Fig. 9(b):B). Higher concentrations of PAM caused apoptosis in the cells with the nonmalignant phenotype (siNOX1), independently of NOX1 and ^1^O_2_. These data show that CAP and PAM cause selective apoptosis induction in tumor cells in a defined window of CAP and PAM dose. This window is defined by the concentration of H_2_O_2_ in CAP and PAM^56^.

#### Mechanistic congruence between corona and transient spark CAP regimes of tumor cell treatment

The data shown in the preceding figures were all obtained after CAP application in the corona regime or with PAM generated by CAP in the corona regime. Supplementary Figs 7–10 show that CAP and PAM application in the transient spark mode, also in atmospheric air, resulted in analogous apoptosis induction in tumor cells as shown for the corona mode. Three different processes after CAP treatment of MKN-45 cells can be detected (Supplementary Figs 7 and 8). Also, catalase was confirmed as primary target for PAM action (Supplementary Fig. 7). The dose responses of both treatments were found to show a similar range (Supplementary Fig. 10).

## Discussion

As apoptosis induction in human gastric carcinoma cells MKN-45 mediated by CAP or PAM generated by a corona plasma pen was dependent on the concentration of CAP or PAM, on time, and on the density of the cells, it seems to be based on intercellular interactions.

As apoptosis induction mediated by CAP or PAM treatment was completely prevented by the ^1^O_2_ scavenger histidine, the central role of ^1^O_2_ for the control of this process is obvious. As addition of histidine 20 minutes after initial treatment no longer caused a strong inhibitory effect, the ^1^O_2_-dependent step must be occuring on a relatively fast timescale. As addition of histidine 2 minutes after CAP treatment still caused complete inhibition of apoptosis, ^1^O_2_ derived directly from CAP was excluded as the major source for active ^1^O_2_, as its life time is is on the order of microseconds. Rather, ^1^O_2_ generated from long-lived species generated through CAP treatment seem to play the essential role. In line with this conclusion, treatment of tumor cells with PAM, allowed a similar degree and kinetics of apoptosis induction as treatment with CAP.

As treatment of tumor cells with CAP for 1 min, followed by 25 min incubation in the same medium was sufficient to inactivate membrane-associated catalase to the same degree as the established catalase inhibitor 3-AT, irreversible inactivation of membrane-associated catalase seemed to be the essential consequence of this initial ^1^O_2_-dependent process. Inactivation of membrane-associated catalase of tumor cells is the crucial step to allow for autocrine apoptotic self destruction of tumor cells through ROS signaling^5,6,9–11,52^. As an incubation step in the CAP-pretreated medium was sufficient for catalase inactivation, whereas the incubation of CAP- treated tumor cells in fresh medium (immediately after CAP treatment) did not allow for detectable catalase inactivation, the role of long-lived species from plasma-activated medium for catalase inactivation was established. In line with this conclusion, treatment of tumor cells with PAM caused the same degree of catalase inactivation as treatment with CAP, when CAP treatment was followed by an incubation step in the same medium. Both processes were characterized by the same inhibitor profile. As 1 min treatment with CAP, without further incubation, was not sufficient to trigger detectable catalase inactivation, the action of ^1^O_2_ directly derived from CAP can be excluded as a sufficient driving force for catalase inactivation under the standard conditions of our experiments. Rather, the central role of the long-lived species is indicated.

**Figure 10:**
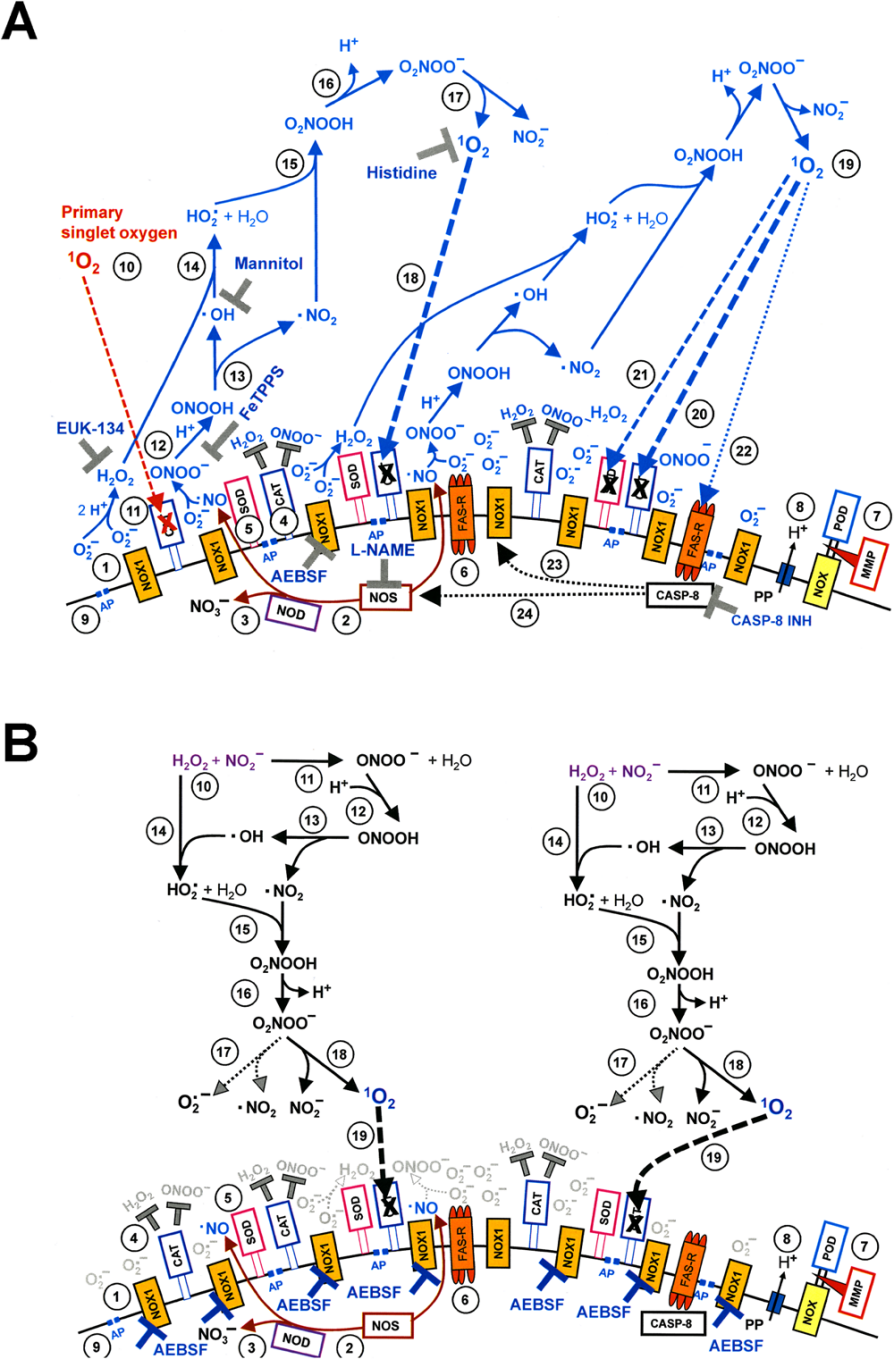
Redox reactions on the surface of tumor cells. (**A**) Biochemistry of secondary singlet oxygen generation. NADPH oxidase 1 (NOX1) is expressed in the membrane of malignant cells and generates extracellular superoxide anions (#1). NO synthase (NOS) (#2) generates ^⋅^NO which can be either oxidised by NO dioxygenase (NOD) (#3) or pass through the cell membrane. Membrane-associated catalase (#4) protects tumor cells towards intercellular ROS/RNS-mediated signaling. Comodulatory SOD (#5) is required to prevent superoxide anion-mediated inhibition of catalase. Further important elements in the membrane are the FAS receptor (#6), Dual oxidase (DUOX) (#7), from which a peroxidase domain is split through matrix metalloprotease, proton pumps (#8) and aquaporins (#9). Primary singlet oxygen from an exogenous source (such as a photosensitizer, long-lived species from PAM or ^1^O_2_ derived from CAP) causes local inactivation of catalase (#10). As a consequence, tumor cell-derived H_2_O_2_ (#11) and ONOO^−^ (#12) are no longer decomposed at the site of inactivated catalase and allow the subsequent generation of secondary singlet oxygen through reactions # 13-#17. These have autoamplificatory potential, as further singlet oxygen-mediated inactivation of catalase (#18) allows for more generation of secondary singlet oxygen (#19). In addition to inactivating catalase (#20), secondary singlet oxygen may inactivate SOD (#21) or activate the FAS receptor independent of its ligand (#22). This causes caspase-8-dependent enhancement of NOX1 and NOS activities. As a result, the generation of secondary singlet oxygen is further enhanced. Figure 10 A also shows the site of action of established inhibitors and scavengers that have been instrumental for the elucidation of the biochemistry of signaling. (**B**) Detection of catalase inactivation by primary singlet oxygen generated from long-lived species in plasma-activated medium. #1-#9 shows redox biology related elements of tumor cells, with the focus on the membrane, as described under A. In order to be able to detect inactivation of membrane-associated catalase by primary singlet oxygen generated from long-lived species present in PAM, i) the generation of secondary singlet oxygen by tumor cells has to be prevented through AEBSF-mediated inhibition of NOX1 and ii) the effective dose of primary singlet oxygen (^1^O_2_) needs to be brought to a substantial level. The increase in the generation of primary singlet oxygen (^1^O_2_) is symbolized by the twofold reaction scheme for its generation. Primary ^1^O_2_ generated through the interaction between H_2_O_2_ and nitrite (#10) is based on reactions #11-#18. Please note that the generation of primary singlet oxygen shows a strong mechanistic overlap (reactions # 12-#18) with the generation of secondary ^1^O_2_ as outlined under A. Inactivation of a substantial concentration of membrane-associated catalase by primary singlet oxygen can be detected through sensitization of the tumor cells for a challenge with exogenous *ONOO*^−^, as determined in the experiment described in Fig. 5.

The detailed inhibitor study of inactivation of membrane-associated catalase after CAP and PAM-treatment revealed that long-lived species derived from CAP/PAM are essential and sufficient to initiate this process, but that the generation of tumor cell-derived secondary ^1^O_2_ is the dominant mechanism to inactivate catalase under standard conditions of our experiments. The dominating role of *secondary* ^1^O_2_ was deduced from the dependence of catalase inactivation on NOX1-derived O_2_^⋅−^ (inhibition by AEBSF), NOS-derived ^⋅^NO (inhibition by L-NAME), requirement for caspase-8 activity and overall dependence on ^1^O_2_ (inhibition by histidine). Together with the inhibitory effect of the ONOO^−^ decomposition catalyst FeTPPS and the ^⋅^OH radical scavenger mannitol, a reaction scheme for the generation of secondary ^1^O_2_ was established (Fig. 10A). This scheme is in complete agreement with the process of secondary ^1^O_2_ generation triggered by the model compounds H_2_O_2_ and NO_2_^−^, i. e. two essential long-lived species in PAM^56^. The dominance of secondary singlet oxygen was also confirmed in two subsequent studies that were based on the quantitative analysis of transmission of induction of ^1^O_2_ generation from plasma pretreated cells to an untreated population^81,82^.

NOX1, NOS and caspase-8 (with its enhancing function on NOX1 and NOS activity^83–85^, (reviewed in ref.^52^) are the driving motor for sustained generation of secondary ^1^O_2_ and its autoamplificatory expansion, followed by extensive catalase inactivation.

Thus the role of primary ^1^O_2_ generated from long-lived species derived from CAP treatment seems to be restricted to a merely triggering function for the “biochemical switchboard” of the tumor cells that is composed of NOX1, catalase and SOD. This then leads to massive generation of secondary ^1^O_2_, which is essentially responsible for catalase inactivation to a degree that allows subsequent intracellular glutathione depletion through influx of H_2_O_2_ and reactivation of apoptosis-inducing intercellular ROS signaling.

Triggering of secondary ^1^O_2_ generation requires the local inactivation of a few molecules of membrane-associated catalase^14,15,52,56,64,77^. This can be achieved by primary ^1^O_2_ (derived from CAP treatment or generated by PAM), but alternatively also by high local concentrations of ^⋅^NO^52,86,87^ or by O_2_^⋅−^^88–91^. O_2_^⋅−^ and ^∙^NO can be easily excluded as triggering molecules in the system studied here, as catalase inactivation required long-lived species derived from CAP.

**Figure 11:**
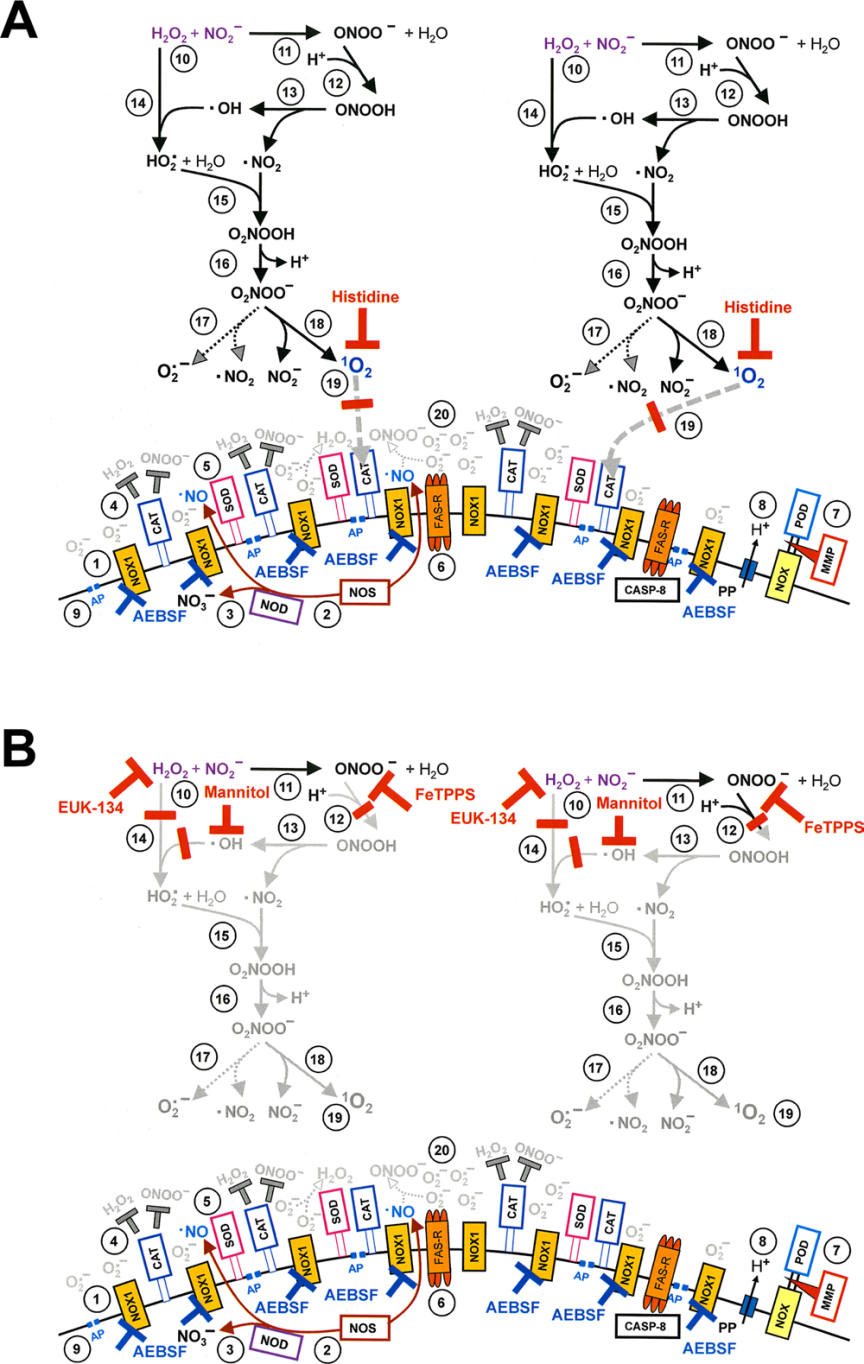
Elucidation of the action of primary singlet oxygen (^1^O_2_) directed towards membrane-associated catalase. (**A**) The NOX1 inhibitor AEBSF prevents the generation of secondary singlet oxygen (^1^O_2_). The singlet oxygen scavenger histidine (HIS) completely prevents inactivation of catalase by primary singlet oxygen (^1^O_2_) generated from long-lived species in PAM and thus ensures that singlet oxygen is the cause of inactivation in this scenario. (**B**) AEBSF prevents the generation of secondary singlet oxygen (^1^O_2_). The addition of either the catalase mimetic EUK-134, or the hydroxyl radical scavenger mannitol (MANN) or the ONOO^−^ decomposition catalyst FeTPPS prevent the generation of primary singlet oxygen (^1^O_2_) derived from long-lived species in CAP-treated medium (#11-#19). Inhibition by these compounds discriminates between primary singlet oxygen generated through interaction between long-lived species in PAM and primary singlet oxygen that is directly derived from the gaseous phase of CAP. Supplementary Figs 11–13 show details of the detection of singlet oxygen directly derived from CAP.

The detection of the effect of primary ^1^O_2_ generated by plasma-activated medium and the study of its generation required a) to block secondary ^1^O_2_ generation through inhibition of NOX1 (or alternatively NOS) and b) to increase the concentration of long-lived species through increasing the time of CAP treatment (Fig. 10B). This allowed the detection of catalase inactivation by a process that was mediated by primary ^1^O_2_ (Fig. 11A) and was dependent on H_2_O_2_, ONOO^−^ and ^⋅^OH radicals (Fig. 11B). This biochemical pathway is in line with recent model experiments with defined compounds^15,56,81^. It defines the interaction between the long-lived species H_2_O_2_ and NO_2_^−^, derived from CAP treatment as the most likely trigger for subsequent generation of ^1^O_2_. As this analysis was performed under conditions of inhibited NOX1, H_2_O_2_ involved in this particular process must have been derived from CAP treatment of medium. For the same reason it was also excluded that ONOO^−^ involved in the process was derived from cellular sources. As ONOO^−^ represents a rather short-lived molecular species, it is excluded that it was derived directly from CAP treatment. Rather the interaction between H_2_O_2_ and NO_2_^−^, two compounds that are known to represent long-lived species in CAP-treated medium seems to define the source of ONOO^−^ that is required for the generation of primary ^1^O_2_.

Even under the conditions of extended CAP treatment, ^1^O_2_ generated directly by CAP did not seem to significantly contribute to catalase inactivation, as the ^1^O_2_-dependent step required the presence of precursor molecules like H_2_O_2_ and ONOO^−^.

**Figure 12:**
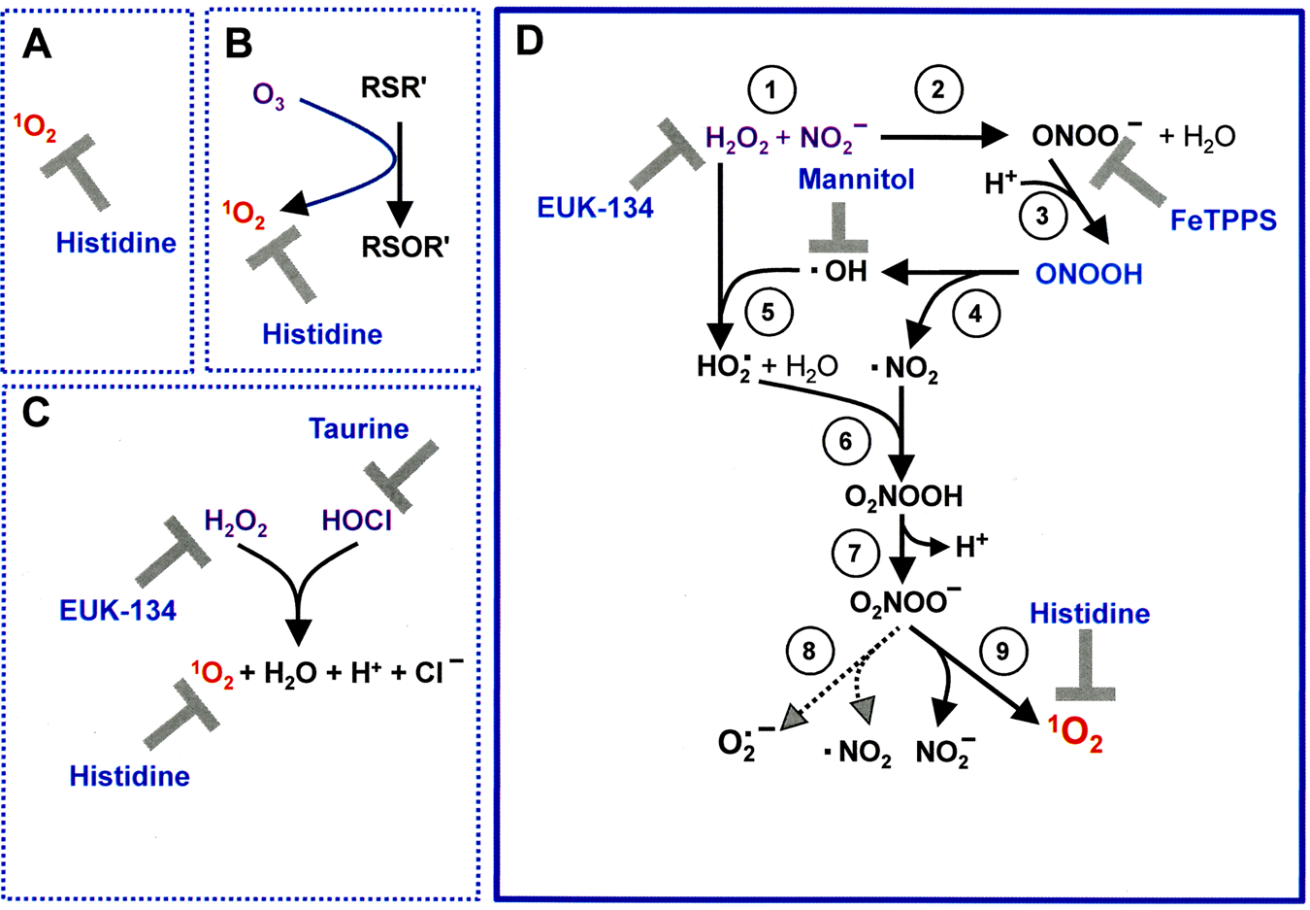
Conceivable ways for the generation of primary singlet oxygen. *(***A**) Primary singlet oxygen (^1^O_2_) directly derived from the gaseous phase of CAP would only be scavenged by histidine. Due to specificity, other inhibitors used in this study would be without any effect on primary ^1^O_2_. (**B**) The interaction between ozone (O_3_) and certain amino acids can lead to the formation of ^1^O_2_. (**C**) The reaction between H_2_O_2_ and HOCl leads to the generation of ^1^O_2_. Inhibition by the catalase mimetic EUK-134, the HOCl scavenger taurine (TAU) and the ^1^O_2_ scavenger histidine (HIS) would be indicative for this pathway. (**D**) The interaction of the long-lived species H_2_O_2_ and nitrite (NO_2_^−^) (#1) leads to the formation of peroxynitrite (ONOO^−^) (#2). Protonation of ONOO^−^ (#3) generates peroxynitrous acid (ONOOH), which decomposes into ^⋅^NO_2_ and ^⋅^OH radicals (#4). The interaction between ^⋅^OH radicals and H_2_O_2_ leads to the generation of hydroperoxyl radicals (HO_2_^⋅^) (#5), which form peroxynitric acid (O_2_NOOH) through interaction with ⋅^∙^NO_2_ (#6). Deprotonation of O_2_NOOH leads to the generation of peroxynitrate (O_2_NOO^−^), which can either decompose into superoxide anions (O_2_^⋅−^) and ^⋅^NO_2_ (#8), or into NO_2_^−^ and ^1^O_2_ (#9).The generation of primary ^1^O_2_ through this pathway has a unique inhibition profile, with strong effects of the ^1^O_2_ scavenger histidine (HIS), the catalase mimetic EUK-134, the ^⋅^OH radical scavenger mannitol (MANN) and the ONOO^−^ decomposition catalayst FeTPPS.

Therefore, among the four conceivable sources for primary ^1^O_2_ (Fig. 12), the generation of ^1^O_2_ through the interaction between H_2_O_2_ and NO_2_^−^ is the most likely (Fig. 12D), whereas ^1^O_2_ directly derived from the gaseous phase of CAP (Fig. 12A) is excluded as major source under the conditions of our assays. The generation of primary ^1^O_2_ through the interaction between H_2_O_2_ and HOCl^92^ (Fig. 12C) was also excluded as essential mechanism, as the inactivation of catalase was not inhibited through the HOCl scavenger taurine. The significance of the initial reaction between H_2_O_2_ and NO_2_^−^ (Fig. 12D), leading to the generation of ONOO^−^ (according to ref.^28^), has also been recognized by Girard *et al*., Kurake *et al*. and Jablonowski and von Woedke^23,24,26^ in their recent studies. Their conclusions were crucial for our experiments and appear to support our model, which extends NO_2_^−^/H_2_O_2_ interaction and ONOO^−^ formation to ^1^O_2_ formation and antitumor cell effects through ^1^O_2_-mediated catalase inactivation.

The reaction of ozone (O_3_) with certain amino acids from the medium might lead to the generation of ^1^O_2_^93,94^ that might target tumor cell protective catalase (Fig. 12B). However, as O_3_ and ^1^O_2_ are relatively short-lived molecular species, this reaction cannot explain the effects observed in our study. The same argument excludes a role for a potential direct catalase inactivation by O_3_^95,96^ as a valid explanation. Furthermore, corona and spark regimes of CAP generation strongly differ in the concentrations of O_3_ generation^27^. Corona creates far more O_3_ than spark. However, both discharge modes mediated apoptosis induction in tumor cells with similar efficiency and through ^1^O_2_. We conclude that the role of O_3_ is at least not dominant, although it may play a minor role.

Only when this experimental approach was further modified by parallel prevention of primary and secondary ^1^O_2_ generation, combined with further extension of the time of CAP treatment, the effect of the ^1^O_2_ that was directly derived from the gaseous phase of CAP was also demonstrated (Fig. 5(b), discussed in Supplementary Figs 11–13). Its concentration seemed to be significantly lower than that generated by long-lived species from PAM. Importantly, the action of gas phase ^1^O_2_ was only detectable in cell cultures of MKN-45 cells that are partially in suspension, but not in cultures of tumor cells like SHEP, SKNMC and others, that are strictly attached to the bottom of the tissue culture cluster. This finding is in line with the small free diffusion path length of ^1^O_2_ in medium. Cells that are in suspension might be affected by gas phase species since these cells will occasionally be exposed to the surface of the medium by mixing. Even a relatively short lived species in liquid such as ^1^O_2_ might be able to react with cells during their time near the gas-liquid interface.

Catalase inactivation through CAP and PAM treatment reactivated intercellular ROS-dependent apoptosis-inducing signaling that was independent of the further presence of CAP or PAM components. Intercellular apoptosis-inducing signaling did not directly require ^1^O_2_, ^⋅^NO, ONOO^−^ and caspase-8 – i. e. the components that were essential for catalase inactivation in the preceding step. Rather, HOCl signaling dominated and was strictly required for apoptosis induction. This conclusion followed from observed strong apoptosis inhibition through any one of the following actions: blocking O_2_^⋅−^ generation; scavenging of O_2_^⋅−^; decomposition of H_2_O_2_; inhibition of peroxidase; and scavenging of HOCl and ^⋅^OH radicals. The inhibitory effect of the caspase-3 inhibitor is in line with caspase-dependent cell death that has been defined as part of the mitochondrial pathway of apoptosis in the subsequently discussed approach of specific siRNA-mediated knockdown of essential signaling elements. The strong inhibition through aquaporin-inhibiting Ag^+^ indicates the role of aquaporins for apoptosis induction, as will be discussed later.

In line with previous reports, the dominant role of HOCl signaling indicates optimal inhibition of catalase to allow this process^8,9^. As HOCl and H_2_O_2_ exhibit a strong suppressive effect on ^⋅^NO/ONOO^−^ signaling^7^, the lack of ^⋅^NO/ONOO^−^ signaling in the present experiments is understandable. However, addition of a fast-decaying exogenous ^⋅^NO donor allows to invert this scenario, inducing suppression of HOCl signaling through consumption of H_2_O_2_ as well as establishment of ^⋅^NO/ONOO^−^ signaling. As both signaling pathways depend on O_2_^⋅−^ coupled with creation of ^⋅^OH radicals near the cell membrane, as well as the presence of functioning aquaporins, a strong mechanistic overlap between the two pathways is recognized.

This study has determined membrane-associated catalase of tumor cells as the central target for primary and secondary ^1^O_2_ that is generated after the action of CAP and PAM. Catalase inactivation by CAP and PAM treatment has been directly demonstrated through application of a ONOO^−^ challenge. The functional relevance of catalase inactivation for cell death can be concluded from reactivation of intercellular apoptosis-inducing ROS signaling, which is under the control of membrane-associated catalase. It is further proven by inhibition of CAP-mediated apoptosis-inducing ROS/RNS signaling through addition of soluble catalase.

This study also demonstrates the complex interaction between catalase and ROS/RNS. Figure 7 defines three distinct roles for catalase in this scenario: Inhibition of HOCl signaling at low concentrations of catalase, interference with the consumption of ^⋅^NO by H_2_O_2_ and HOCl at intermediate concentrations, and interference with ^⋅^NO/ONOO^−^ signaling at high concentrations. The very high concentration of soluble catalase required for the inhibition of ^⋅^NO/ONOO^−^ signaling results from the steric and kinetic problem of interfering with a highly efficient process that is exclusively localized close to its target, the cell membrane^9^. Tumor cells solve this problem through attachment of catalase to the membrane and thus generate a high local density of catalase at the site where it is actually needed for their protection.

Most conclusions in this study are based on inhibitor studies, which were complemented by an approach that utilized siRNA-mediated knockdown of signaling relevant elements in the target cells (Supplementary Fig. 3). This analysis confirms the role of NOX1, NOS, TGF-beta and its receptor, protein kinase C zeta, the mitochondrial pathway of apoptosis with caspases-9 and -3, as well as the FAS receptor and caspase-8 that are required for enhancement of NOX1 and NOS activities during the initial steps. These basic data require parallel assessment of inhibition data to draw the precise picture of signaling events. They are perfectly in line with the findings for the model compounds H_2_O_2_ and NO_2_^−^ ^56,81^ and with previous studies on siRNA-based analysis of intercellular ROS/RNS signaling^4,13^.

Is it very likely that other authors who have documented the effects of CAP on tumor cells have identified mechanistic sequences that are contained in our model. This certainly seems to be the case for the model established by Yan *et al*.^44,45^.

It seems that in reports on caspase activation as well as on an increase of intracellular ROS after CAP treatment, the authors have identified “secondary” responses, such as activation of the mitochondrial pathway (with its inherent caspase activation and ROS generation by uncoupled mitochondria) as the “primary” effects of CAP.

Several groups have shown that addition of the broadly acting antioxidant N-acetyl cysteine abrogates CAP effects. This is consistent with our model, but does not imply that RONS act exclusively by entering cells and inducing observed effects just by that ingress.

In their recent review, von Woedtke *et al*.^97^ raised a series of important questions. It seems, we have provided adequate answers to these questions in this manuscript. Their question “*are there single and specific ROS and RNS responsible for distinct biological effects or is it only a matter of redox potential of the cellular target sites?*” has been answered by our experiments: ^1^O_2_ mainly generated through the interaction between long-lived species in CAP and PAM attacks catalase that is specifically located on the membrane of tumor cells. Due to the parallel expression of NOX1 in the membrane of tumor cells, the attack on catalase by primary ^1^O_2_ provokes secondary ^1^O_2_ generation, further catalase inactivation, intracellular glutathione depletion and intercellular RONS-mediated apoptosis signaling. Therefore, specific CAP action towards tumor cells requires i) defined compounds generated by CAP and their interaction with ii) defined redox-related elements specifically expressed on the surface of tumor cells.

The second central question was: “*How to identify and analyze specific ROS and RNS at their site of action?*”. This question has been answered through multiple strategies. One strategy was to use reconstitution experiments, i. e. the addition of RONS that are known to be active in CAP and to test what effect they have compared to CAP exposure. Another strategy was to use inhibitors and scavengers with CAP exposure. These compounds offer a defined degree of specificity in action and thus allow to analyze individual steps in a proposed mechanism. This approach was coupled with varying incubation times and washing sequences. A third strategy used siRNA to target individual genes to test for the role of the knocked-down enzymes in the proposed model. A fourth strategy was to test the effects of cell-cell communication by adding varying numbers of CAP-treated cells to untreated cells to detect non-linear (i.e. amplification) effects of treated cells on collections of untreated cells^82^.

Finally, the question: “*Is it possible to find a measure for biological plasma effects that can servce as a kind of “treatment dose*?” can be addressed by the comment that this can only be done with a firm grasp of the mechanisms that CAP uses to effect the desired cellular responses. The key point is that the appropriate doses therefore depend on understanding the mechanisms. The mechanism presented in our manuscript represents a basic mechanism that is applicable to dense populations of tumor cells. Therefore, further studies are required to define the requirements for the treatment of other constellations, for example sparsely distributed tumor cells after excision of a tumor.

Tumor cells are protected towards intercellular ROS/RNS signaling through tight control based on membrane-associated catalase that is supplemented by comodulatory SOD on the membrane of the cells. As SOD, like catalase, has histidine in its active center, CAP/PAM-triggered primary ^1^O_2_, and more likely tumor cell-derived secondary ^1^O_2_ can inactivate SOD in parallel to catalase^62,63^. Based on an established synergistic effect induced by parallel inhibition of membrane-associated catalase and SOD of tumor cells by neutralizing single domain antibodies directed towards catalase or SOD^53^, it can be predicted that parallel inactivation of catalase and SOD by ^1^O_2_ should result in an analogous synergistic effect^21^. It is most likely that this synergistic effect contributes to the high efficiency of CAP and PAM action.

The treatment of tumor cells with CAP or PAM finally results in ^1^O_2_-mediated inactivation of catalase to a degree that allows for reactivation of intercellular HOCl signaling. As apoptosis induction through the HOCl pathway, as well as through the ^⋅^NO/ONOO^−^ pathway is prevented through inhibition of aquaporins, their dominant controling function is confirmed. This is in line with the basic findings by Yan *et al*.^44,45^. However, the role of aquaporins does not seem to be prominent as determinining factor for responsiveness of cells, but rather seems to have a strong impact after catalase inactivation and before the onset of apoptosis induction. As the effect of inhibition of aquaporins can be abrogated through glutathione depletion through pretreatment with an inhibitor of GSH synthesis, the role of aquaporins is most likely to allow an influx of H_2_O_2_ that leads to intracellular depletion of GSH. This then prevents the repair of lipid peroxidation by glutathione peroxidase-4 and GSH^98^. In line with this conclusion, the kinetics of apoptosis induction in glutathione-depleted tumor cells through BSO treatment does not show the initial lag phase that is typical for the action of CAP on control tumor cells, and also is no longer inhibited by the aquaporin inhibitor Ag^+^. However, this kinetics is still completely blocked by inhibitors of HOCl signaling, indicating that this pathway, and not intruding H_2_O_2_ itself, triggers apoptosis induction.

The previously established role of proton pumps for the generation of ONOOH from ONOO^−^ and, to a lesser extent for the generation of HOCl from OCl^−^ anions^52^ was confirmed in this study. The differential inhibitory response at different time points correlates well with the differential requirement for ONOOH during the initial inactivation of catalase and for HOCl during subsequent apoptosis-inducing signaling.

In addition to human MKN-45 gastric carcinoma cells, also human neuroblastoma, Ewing sarcoma and cervical carcinoma cells where shown to respond to CAP treatment with NOX-1 dependent apoptosis induction, mediated by ^1^O_2_. The inhibition profile by the ^1^O_2_ scavenger histidine indicated that in all these cell lines the long-lived species from CAP treatment were essential to trigger apoptosis induction. As protection of tumor cells by membrane-associated catalase has been found a regular feature of tumor cells, their reaction was not unexpected. However, due to variable concentrations of O_2_^−^ versus ^⋅^NO, tumor cells do not necessarily reactivate HOCl signaling after catalase inactivation. In some tumor systems, ^⋅^NO/ONOO^−^ signaling is prevailing.

Apoptosis induction in nonmalignant diploid fibroblasts required longer CAP treatment than apoptosis induction in tumor cells. It was not dependent on extracellular superoxide anions, which is in line with the lack of sustained NOX expression in nonmalignant cells. ^1^O_2_ did not seem to play a determining role for apoptosis induction which seemed to be mediated by H_2_O_2_.

Model experiments with tumor cells that were positive for NOX1 and membrane-associated catalase and perfectly matched control cells withough NOX1 expression and lack of membrane-associated catalase allowed to define the switch between selective and nonselective apoptosis induction by long-lived species in CAP and PAM more precisely. Low concentrations of CAP or PAM derived species were sufficient to trigger apoptosis induction in tumor cells, provided NO_2_^−^ was present to allow for synergistic interaction, based on the generation of ^1^O_2_. Under these conditions, nonmalignant cells, due to their lack of catalase and NOX1 were not affected. This scenario describes the window of selective CAP and PAM action. At higher concentrations of PAM, nonmalignant cells went into H_2_O_2_-dependent cell death, due to their lack of catalase. Therefore, the secret of selective PAM and CAP action is to find the window where ^1^O_2_ generation is achieved at concentrations of H_2_O_2_ that do not affect nonmalignant cells.

This study shows that low concentrations of primary ^1^O_2_ are generated by long-lived species derived from CAP treatment of medium. This triggers a massive, autoamplificatory generation of secondary ^1^O_2_ by the tumor cells, resulting in substantial inactivation of their membrane-associated catalase. After parallel intrusion of H_2_O_2_ through aquaporins, apoptosis-inducing HOCl signaling is then reactivated and causes cell death through the mitochondrial pathway of apoptosis. Apoptosis induction by Fenton chemistry of the intruding H_2_O_2_ has been excluded as significant contribution to tumor cell death, as inhibition of HOCl signaling completely prevented apoptosis induction, despite completed glutathione depletion by intruding H_2_O_2_.

Based on recent insights into the role of immunogenic cell death^99–106^, it can be assumed that the initial apoptosis induction triggered by CAP and PAM might activate adequate T cell responses *in vivo* that finalize the process of tumor destruction. These aspects are further discussed under Supplementary Discussion.

The demonstration of the potential of H_2_O_2_ and nitrite to efficiently trigger selective apoptosis induction in tumor cells *in vitro* explains the established antitumor effects of PAM with its restriction to long-lived molecular species. However, it does not mean that CAP treatment might have no additional benefits compared to the application of only H_2_O_2_ and NO_2_^−^. One aspect that is essential in this context and that requires more elucidation is the stimulation of immunological processes by CAP. These are most likely triggered by immunogenic cell death, but are potentially also enhanced by CAP constituents different from the species studied here. Furthermore, under conditions, where relatively short-lived CAP-derived species like ONOO^−^ or ^⋅^NO do actually reach the tumor cells, a valuable synergistic effect of these two species with the H_2_O_2_/NO_2_^−^-mediated, ^1^O_2_-dependent processes is feasible, based on the results from a previous study^77^.

Furthermore, the combination of increased ^1^O_2_ production by the plasma source with lowering the layer of medium can be expected to enhance the biological effects *in vitro* of ^1^O_2_ derived from the gaseous phase of plasma. It is predictable that these modifications might cause an increase in the generation of secondary ^1^O_2_ by the tumor cells. *In vivo*, analogous results might be obtained by a combination of increased primary ^1^O_2_ production by the plasma source with a closer contact of the source to the tumor. These considerations are also valid for the analysis of potential ozone effects. They also might be applicable to the evaluation of potential synergistic effects that are possibly established when CAP-derived NO, peroxynitrite or NO_2_ interact with ^1^O_2_ generation and ^1^O_2_-mediated processes. Furthermore, the biological significance of hypochlorite/hypochlorous acid as demonstrated by Bekeschus *et al*.^107^ and the well characterized chemical biology of hypochlorite/hypochlorous acid and dichloride anion radicals, as published by Wende *et al*. and Jirisek and Lukes^108,109^ should encourage further studies on the effects of chlorine/chloride-related compounds in CAP and PAM. Interference of hypochlorous acid with catalase activity discussed by Krych-Madej and Gebicka^110^ defines another path of interest in this context.

Though our own study has defined one central path that is sufficient to cause induction of apoptosis specifically in tumor cells, triggered by long-lived species derived from CAP and PAM, the search for additional active ROS/RNS (or their reaction products) in CAP and PAM should continue. We hope that our study has defined a new conceptual and experimental approach, with the potential to analyze and to optimize CAP and PAM applications. Hopefully, this may have a positive impact on CAP- and PAM-based tumor therapy in the future.

The data shown in our present study are applicable to dense populations of tumor cells, as key processes in this scenario are cell density-dependent. Therefore, the removal of residual tumor cells by plasma treatment after resection of tumors, as established by Canady *et al*.^111^ can be predicted to possibly require different species as well as a different biochemical process, as the most likely lower cell density of residual tumor cells compared to a tumor has a predictable impact of cell density signaling processes like the generation of secondary ^1^O_2_ and intercellular apoptosis-inducing signaling. The modulation of the composition of RONS generated by CAP and the knowledge of the redox-relevant composition of the surface of tumor cells open a chance to resolve this potential problem.

## Concluding Remarks

The anti-tumor mechanisms of cold atmospheric plasma (CAP) and plasma-activated media (PAM) have generally been acknowledged to involve the creation of reactive oxygen and nitrogen species (RONS). These species are known to play multiple biochemical roles in wound healing, fighting infections and tumors, among others, including involvement in multiple existing non-plasma based therapies. However, there is less appreciation for the fact that cells themselves, and especially tumor cells, often are prolific sources and sinks for RONS.

The direct application of plasma to tissue takes place at external or internal surfaces and this application rarely exceeds several minutes of exposure. By contrast, the biological effects of this spatially localized and relatively brief application extend over timescales of hours to days and possibly longer. And the effects of the plasma can extend over tissue depths of distances of more than centimeters. There are clearly, in general, significant non-local effects involved in CAP and PAM treatments in cancer therapy and in other applications. Indeed, for any effective therapy of extended tissue or organisms, the therapeutic effects must be correspondingly extensive. In the special case of cancer tumor treatment, the question manifests itself as how the interior parts of a solid tumor could be affected by treatment at its surface boundaries.

It seems clear from this analysis that there must be some non-local responses to the local plasma treatment. Possible ways this could occur include effects on local blood flow; stimulation of the organism’s immune system; and some form of cell-cell communication. It is the latter effect that we primarily focus on here. We show how the natural intercellular and extracellular signaling machinery involving the creation and removal of RONS is responsible for cell-cell apoptotic signaling that can lead to selective tumor cell death.

The present paper describes extensive experiments using different cell types, both malignant and nonmalignant, with CAP in both corona and spark regimes of operation. Experiments using enzyme inhibitors; reactive species scavengers; reactive species donors; mimetics; and gene (enzyme) knockdowns, allowed the development of a detailed mechanistic picture of CAP and PAM induced selective apopotosis of tumor cells relative to nonmalignant cells.

In particular, in the context of *in vitro* experiments, we show that CAP or PAM application to tumor cells works by triggering tumor cells to no longer decompose extracellular RONS that they constantly generate. This finally results in their own apoptosis. The key triggering step involves inactivation of surface membrane associated catalase by singlet oxygen created by CAP or PAM chemistry. This triggering step occurs by a short (~1 minute) exposure of CAP to the medium, followed by liquid phase reactions between CAP- or PAM-generated species that take place over ~10–15 minutes. These relatively brief steps create what we refer to as ‘primary’ singlet oxygen in the liquid medium in relatively small numbers, but they inactivate at least a few of the membrane-bound catalase enzymes of tumor cells in solution. A similar process occurs with reconstitution experiments in which the known CAP-generated species (NO_2_^−^ and H_2_O_2_) are added to solution directly rather than through CAP or PAM addition.

Remarkably, after this initial ‘triggering’ step, the role of CAP and PAM ceases. Once a few tumor cells have had their membrane catalase inactivated, these cells themselves generate large amounts of what we refer to as ‘secondary’ ^1^O_2_. The burst of secondary ^1^O_2_ serves to inactivate adjacent cell membrane catalase enzymes, thus providing a mechanism for spatial propagation of the initial trigger. In addition, the absence of membrane-bound catalase allows tumor cell-generated RONS to initiate an apoptotic process that takes place over typically 3–5 hours. This apoptotic process is multi-step, and we demonstrate that if any of the individual steps is eliminated or significantly altered, apoptosis is halted.

The apoptotic pathway identified here involves cellular mitochondria, is initiated by lipid peroxidation (LPO) of the cellular membrane and affected by the oxidizing effects of H_2_O_2_ that enter the cell via aquaporins. The LPO occurs via ^⋅^OH radical attack of cell membrane lipids, and these radicals are created by HOCl/O_2_^∙−^ interaction very near the membrane surface. The HOCl is created by a peroxidase (POD) enzymatic reaction between H_2_O_2_ and Cl^−^. HOCl reacts with membrane-localized O_2_^⋅−^ to form products including ^⋅^OH, and some of this ^⋅^OH peroxidizes the lipid membrane, an important step in the sequential pathway associated with tumor cell apoptosis.

Importantly, lipid peroxidation only seems to effectively induce the mitochondrial pathway of apoptosis when an influx of H_2_O_2_ into the cells has lowered the intracellular glutathione level. This finding connects our study to previous work by Keidar and Yan^44,45^.

All the RONS involved in this apoptotic pathway come from cellular processes and have no connection to CAP or PAM: ^⋅^NO is created within the cell via nitric oxide synthase (NOS); O_2_^⋅−^ is created near the cell exterior membrane boundary by membrane bound enzymes (NADPH Oxidase1, or NOX1). NOX1-generated O_2_^⋅−^ dismutates in the near-cell region to form H_2_O_2_ and also reacts with ^⋅^NO to form ONOO^−^. Proton pumps in the cell boundary act to reduce pH near the exterior cell membrane, and this is important for several key reactions. In the cell interior, the most prominent antioxidant is glutathione (GSH). This compound can repair LPO damage and can help protect the cell from oxidation damage associated with the influx of H_2_O_2_ through aquaporins in the cell membrane. Reactions among these species generate all the intermediate and final RONS products involved in the apoptotic pathway.

The overall process starts with a brief CAP- or PAM-initiated inactivation of membrane associated catalase. The subsequent steps involve spatial propagation of an apoptotic ‘wave’ through tumor cells that we term auto-amplification or autocrine activation. This RONS-based mechanism is initiated by CAP or PAM, and acts over time and length scales that are far larger than the original plasma or plasma-treated medium exposure. The mechanism relies on cellular extracellular and intracellular RONS signaling. Thus, we conclude that CAP and PAM act only as a trigger for a natural RONS-based apoptotic pathway, extending over longer time and space scales than the original stimulation. We suggest that these observations represent an important general feature of CAP and PAM based therapy: most of the RONS-based therapeutic signaling is only *initiated* by CAP/PAM. The triggered tumor cells are not at all passive: they do most of the subsequent work on their own.

## Supplementary information

Supplementary Information

## Data Availability

All data generated or analyzed during this study are included in this published article (and its Supplementary Information Files).
