# Supplementary material for: Cold Atmospheric Plasma and Plasma-Activated Medium Trigger RONS-Based Tumor Cell Apoptosis
Source: Sci Rep. 2019 Oct 2;9:14210. doi: 10.1038/s41598-019-50291-0 (PMC6775051; doi:10.1038/s41598-019-50291-0)
Supplement: Supplementary file 1 — Supplementary Information [file 41598_2019_50291_MOESM1_ESM.pdf]

Supplementary material

Cold atmospheric plasma and plasma-activated medium trigger RONS-based tumor cell apoptosis

Georg Bauer, Dominika Sersenová, David B Graves and Zdenko Machala

A. Supplementary Introduction

Cold atmospheric plasma (CAP) contains electrons, photons and a pleothora of radical and nonradical reactive oxygen and nitrogen species (RONS) in its gaseous and/or liquid phase. Most important are superoxide anions ($O_2^{\bullet -}$), hydroperoxyl radicals ($HO_2^{\bullet}$), hydrogen peroxide (H_2O_2), hydroxyl radicals ($^{\bullet}OH$), atomic oxygen (O), singlet oxygen ($^1O_2$), ozone (O_3), nitric oxide ($^{\bullet}NO$), nitrogen dioxide ($^{\bullet}NO_2$), peroxynitrite ($ONOO^-$), nitrite (NO_2^-), nitrate (NO_3^-), dichloride radicals ($Cl_2^{\bullet -}$) and hypochloride anions (OCl^-) (Machala et al., 2007; Stoffels et al., 2008, Fridman et al., 2008; Graves 2012, 2017; Laroussi 2009, 2014, 2015; Laroussi et al., 2017; Lu et al., 2016; Graves 2014 a,b; Jablonowski et al., 2015; Sousa et al., 2011, Machala et al., 2013; Wende et al., 2015; Schmidt-Bleker et al., 2016, Gianella et al., 2016; Jirasek and Lukes, 2019). CAP-derived ROS and RNS represent a unique scenario of ROS/RNS chemical biology, based on variable life-times, ranges of actions and multiple potentials of interactions.

In most studies that directly compared tumor cells with nonmalignant cells, CAP and PAM were found to act selectively towards malignant target cells *in vitro* and *in vivo* (Kalghati et al., 2011; Tanaka et al., 2011; Zucker et al., 2012; Wang et al., 2013;

Guerrero-Preston et al., 2014; Kaushik et al., 2014; Utsumi et al., 2014; Siu et al., 2015; Kim and Chung, 2016; Kurake et al., 2016; Duan et al., 2017; Canal et al., 2017; Liedke et al., 2017). Only a few reports claimed nonselective apoptosis-inducing effects of CAP or PAM (Wende et al., 2014; Hirst et al., 2015; Bekeschus et al., 2013, 2015; Bundscherer et al., 2013). It has been suggested that this discrepancy might be resolved by standardization of CAP and PAM doses and composition (Bauer, 2019).

B. Supplementary Results

1. The dependency of the effect of CAP treatment on the treatment time and on cell density

When MKN-45 cells were treated with CAP (Corona regime) for increasing times and apoptosis induction was determined after 4 h, 30 s of CAP treatment were sufficient to trigger nearly the maximal apoptosis-inducing effect, whereas 15 s treatment remained without significant effect (Supplementary Figure 1). The nature of the plateau-type of curve obtained in this experiment is in line with the finding that CAP causes initial effects on tumor cells that are multiplied by autoamplificatory mechanisms. In line with the central role for CAP-mediated apoptosis induction, the $^1O_2$ scavenger histidine caused a strong inhibitory effect in this experiment.

Treatment of tumor cells at varying cell density with CAP showed that the optimal effect of CAP was dependent on high cell density. When cells were treated with CAP at the highest cell density and then were shifted to lower density, the optimal effect of treatment at the highest density remained (Supplementary Figure 2). These findings show that cells seem to contribute to CAP-mediated effects and that the initial, density-dependent effects are very fast.

Supplementary Figure 1

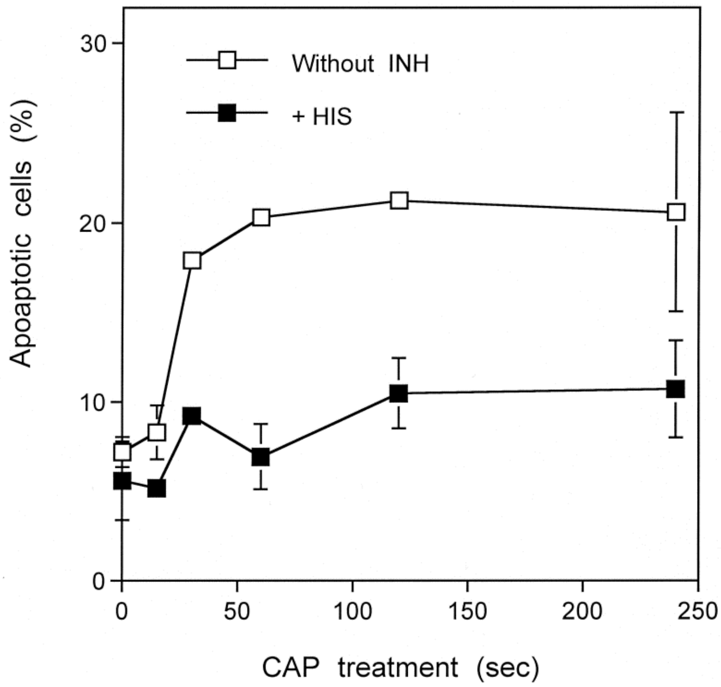

Supplementary Figure 1: *Dependency of apoptosis induction in tumor cells on the time of CAP treatment*. MKN-45 cells were treated with CAP for the indicated times, in the absence or presence of the $^1O_2$ scavenger histidine. The percentages of apoptotic cells were determined after 4 h. The result confirms that CAP-mediated apoptosis induction is mediated by $^1O_2$. The dose-response curve is characterized by a steep increase, followed by a plateau.

Statistical analysis: Apoptosis induction by CAP treatment for 30 min or longer, as well as inhibition by histidine was highly significant ($p<0.001$).

Supplementary Figure 2

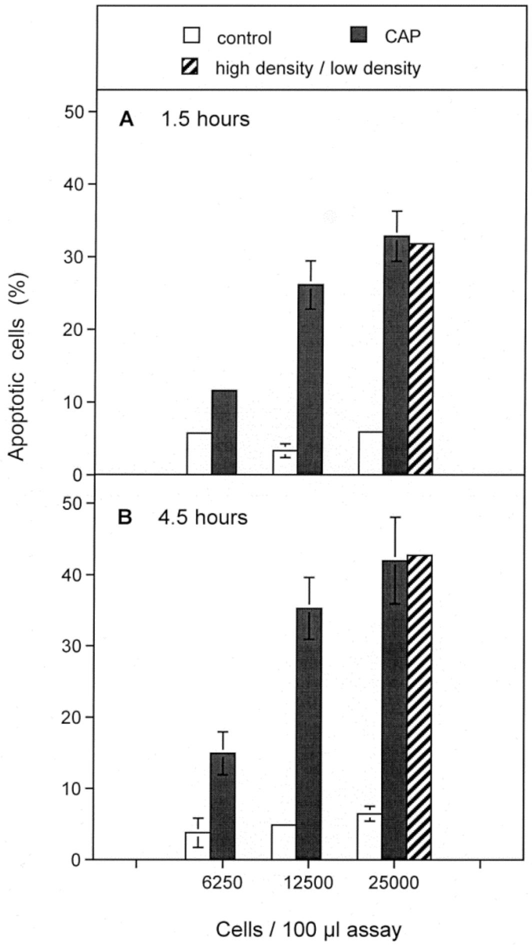

Supplementary Figure 2: *Dependency of CAP-mediated apoptosis induction in tumor cells on the cell density.* MKN-45 tumor cells were seeded in 96 well plates at the indicated densities and remained either untreated (control) or where treated with CAP for 1 min (CAP). The percentages of apoptotic cells were determined after 4.5 h.

In some assays (labelled as "high density/low density"), the cells were treated with CAP for 1 min at a density of 25 000 cells per 100 µl assay and where further incubated for 25 min ("high density/low density"). These cells were then washed, reseeded at a density of 12 500 cells/assay and further incubated for 4 h.

These results show that CAP-mediated apoptosis induction is dependent on the density of the target cells.

Statistical analysis: Apoptosis induction by CAP at all cell densities was highly significant ($p<0.001$).

2. SiRNA-based analysis of CAP–mediated effects

SiRNA-based knockdown of potential signaling components has been found to be instrumental for the analysis of intercellular ROS/RNS-mediated apoptosis-inducing signaling (Bauer, 2017 a, b). Therefore, the expression of potentially interesting candidate genes was knocked-down and the effect of knockdown on CAP-mediated apoptosis induction was determined. As shown in Supplementary Figure 3, control cells (“SiCo”) that had been treated with irrelevant control siRNA that did not affect any known gene responded to CAP treatment with apoptosis induction. Knockdown

Supplementary Figure 3

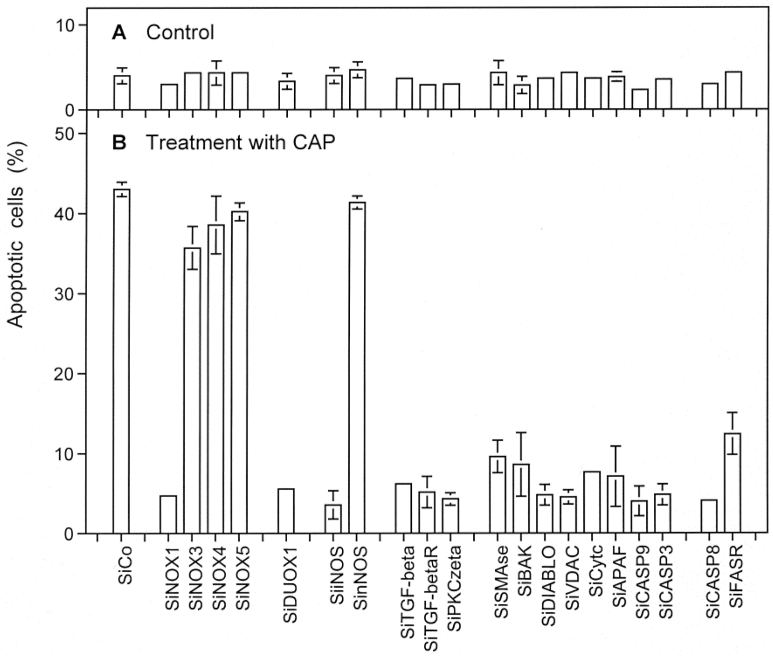

Supplementary Figure 3: *SiRNA-based analysis of CAP–mediated effects.* MKN-45 were treated with siRNAs directed towards the indicated targets or with control siRNA for 24 h. The cells then remained either untreated (A) or were treated with CAP for 1 min (B). The percentages of apoptotic cells were determined after 3 h. The result defines cellular functions that are essential for CAP-mediated apoptosis induction, a process that is determined by a) $^1O_2$-mediated inactivation of catalase, b) influx of H_2O_2 through aquaporins and c) intercellular apoptosis inducing signaling. As outlined in the text, the role of some cellular functions may be restricted to one specific step (e. g. iNOS, caspase-8), whereas others may contribute to all three steps (e. g. NOX1).
Details of siRNAs, knockdown and control of efficiency can be found in Bauer, 2017 a.

Statistical analysis: Apoptosis induction in siCo cells was highly significant ($p<0.001$). Inhibition of CAP-mediated apoptosis induction by knockdown with siNOX1, siDUOX1, siiNOS, siTGF-beta, siTGF-beta receptor, siPKC-zeta, siSMase, siBAK, siDIABLO, siCytochrome C, siAPAF, siCASP-9, 8, 3, and siFAS receptor was highly significant ($p<0.001$).

of NOX1, but not of NOX3, NOX4, NOX5 caused complete inhibition, demonstrating the role of superoxide anions generated by NOX1. NOX1-derived superoxide anions are essential for catalase inactivation through secondary $^1O_2$, for the generation of H_2O_2 that passes aquaporins and lowers the intracellular GSH level and for HOCl signaling (generation of the peroxidase substrate H_2O_2 and interaction of superoxide anions with HOCl to yield apoptosis-inducing hydroxyl radicals). DUOX was defined in this experiment as the source for peroxidase, which is required for HOCl signaling after catalase inactivation, but not for the process of catalase inactivation itself. In contrast, iNOS, but not nNOS, is responsible for the generation NO that is required for secondary generation of $^1O_2$, but not for apoptosis-inducing signaling. The role of TGF-beta, its receptor and of protein kinase C-zeta is explained by the controlling function of these elements on NOX1 activity (please see Bauer, 2017 a for details). The inhibitory effect of knockdown of sphingomyelinase, diablo, VDAC, Cytochrome c, APAF, caspase-9 and caspase-3 is indicative of the dominant role of the mitochondrial pathway of apoptosis (Bauer, 2017 a). The requirement for caspase-8 and the FAS receptor is explained by their role for enhancement of NOX1 activity and NOS expression during the generation of secondary $^1O_2$. As shown below, the requirement for FAS receptor and caspase-8 is not indicative of any contribution of death receptor-mediated apoptosis induction to CAP-determined apoptosis induction.

3. Differential roles of caspases during CAP and PAM-mediated apoptosis induction

When tumor cells were treated with the long-lived species in PAM that are derived from CAP treatment, caspase-8 only had an inhibitory effect if it was added before

PAM (Supplementary Figure 4). The caspase-8-dependent effect was very fast, as addition of caspase-8 25 min after PAM did not allow to detect any inhibitory effect. This finding is in line with the stimulatory role of FAS-activated caspase-8 for the enhancement of NOX1 and NOS (Zhuang et al., 2001: Suzuki et al., 1998; Reinehr et al., 2005; Selleri et al, 1997; reviewed in Bauer, 2015), rather than with a role of death receptor-mediated apoptosis induction in this process. In this scenario, the FAS receptor seems to be activated by $^1O_2$ (Zhuang et al., 2001, Bauer, 2015). In contrast to caspase-8, caspase-3 and caspase-9 seemed to play a role late in signaling which is compatible with its role in the mitochondrial pathway of apoptosis.

Supplementary Figure 4

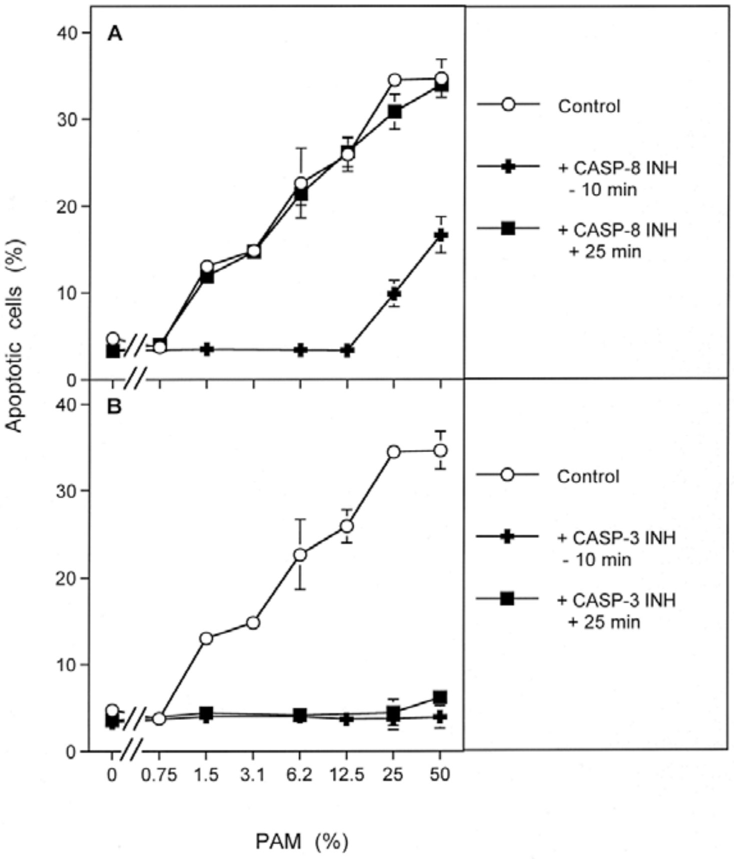

Supplementary Figure 4. *Differential roles of caspase-8 (A) and caspase-3 (B) during CAP and PAM-mediated apoptosis induction.* MKN-45 cells were treated with increasing concentrations of PAM (generated by 1 min treatment of medium with CAP), in the absence or presence of 25 µM caspase-8 inhibitor or 50 µM caspase-3 inhibitor. The inhibitors had

been either added 10 min before addition of PAM or 25 min after PAM addition, as indicated in the figure. The percentages of apoptotic cells were determined 3.5 h after addition of PAM: The results show an involvement of caspase-8 in an early step, in line with its role for enhancement of NOX1 and NOS during the generation of secondary $^1O_2$. In contrast, the role of caspase-3 is related to late effects, in line with its known role for execution of apoptosis. *Statistical analysis: Apoptosis induction by 1.5 % PAM and higher concentrations was highly significant (p<0:001). Inhibition by caspase-8 inhibitor added before PAM and by caspase-3 inhibitor added at both times was highly significant (p<0.001).*

4.Inactivation of membrane-associated SOD of tumor cells by CAP

Tumor cells are protected towards intercellular ROS/RNS signaling through a tight control based on membrane-associated catalase that is supplemented by comodulatory SOD on the membrane of the cells (Bauer, 2014; Bauer and Motz, 2016). The function of SOD is to lower the concentration of free superoxide anions below a level where catalase can be inhibited by them. The removal of superoxide anions by SOD is not complete. Therefore, despite the presence of active SOD on the membrane, superoxide anion-dependent processes like the interaction between HOCl and superoxide anions, leading to apoptosis inducing hydroxyl radicals, are still possible (Bauer, 2013). Inhibition of SOD by neutralizing single domain antibodies causes an increase in free superoxide anions that lead to a leftward shift of the concentration-dependency curve for the action of HOC (Supplementary Figure 5 B). An analogous shift of the concentration curve is obtained after treatment of the cells with CAP. This is in line with inactivation of SOD by CAP. For the evaluation of the significance of this conclusion, it is important to reflect on the kinetic situation during HOCl-mediated apoptosis induction. HOCl brought into the system in one step during bolus addition is confronted with a defined concentration of free superoxide anions close to the surface of tumor cells. Numerous other molecules in the medium are competing for HOCl in parallel and can be expected to consume a significant part of total HOCl. An increase in the available concentration of superoxide anions through inactivation of SOD will increase the chance of HOCl to react with superoxide anions

rather than with irrelevant material. This then contributes to the leftward shift of the concentration-dependency of of HOCl action on the cells.

It is predictable that the resultant superoxide anions have a negative impact on the activity of catalase. They thus contribute to overall catalase inhibition and enhancement of the generation of secondary $^1O_2$ in a synergistic mode (Bauer, 2019).

Supplementary Figure 5

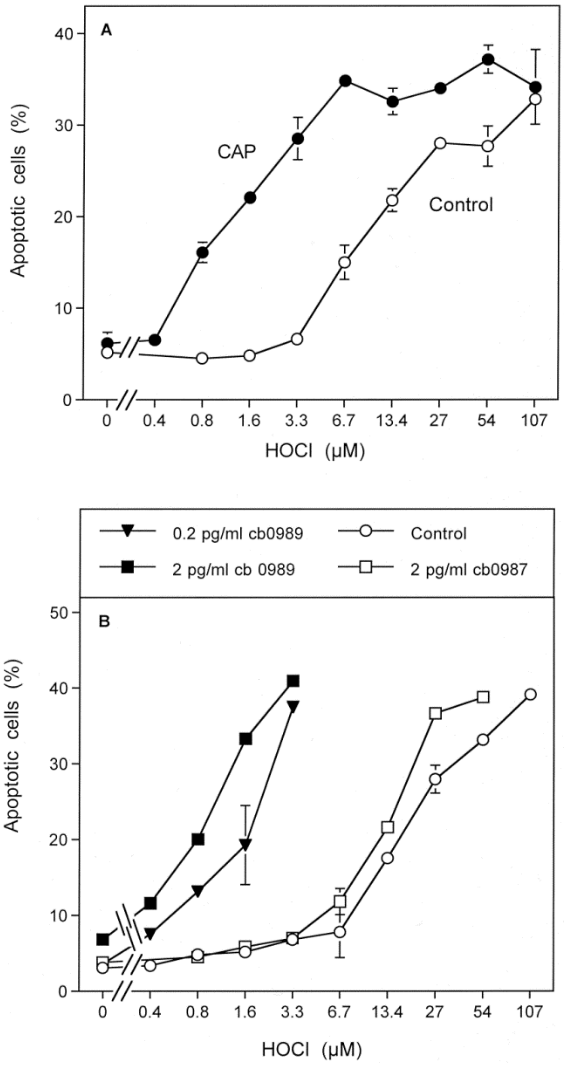

Supplementary Figure 5. *Inactivation of membrane-associated SOD of tumor cells by CAP.* A. MKN-45 cells were treated with CAP for 1 min, followed by 25 min incubation, or remained untreated (control). Then the assays received 2 µM EUK-134 to ensure the decomposition of cell-derived H2O2 that has a negative effect on HOCl. HOCl was then added at the indicated concentrations and the percentages of apoptotic cells were determined 1 h later.

B. The tumor cells were treated with the indicated concentrations of the SOD-neutralizing single domain antibody cb0989 or with single domain antibodies cb0987 that bind to SOD, but do not affect its activity. Further control assays remained without antibodies. After 10 min incubation at room temperature, the assays received 2 µM EUK-134 and the indicated concentrations of HOCl. Apoptosis induction was monitored after 1 h.
These results show that CAP-treatment of tumor cells has the same effect as neutralizing single domain antibodies that are directed towards SOD. The leftward shift of the concentration-dependency curve is explained by the increased concentrations of free superoxide anions after SOD inactivation.
Statistical analysis: A: Apoptosis induction by HOCl at 6.7 µM and higher in the controls and by 0.8 µM and higher in the CAP-treated assays was highly significant. The shift between the two curves is highly significant ($p<0.001$). B: Apoptosis induction by 13.4 µM HOCl (control curve, open circles) and higher, as well as the shift of the curves obtained in the presence of cb0989 was highly significant ($p<0.001$).

5. The effect of CAP on neuroblastoma, Ewing sarcoma and cervical carcinoma cells

Treatment of human neuroblastoma SHEP, Ewing sarcoma SKN-MC and cervical carcinoma cells SIHA with had the same effect as treatment of MKN-45 cells that have been used in most experiments described in this manuscript (Supplementary Figure 6). CAP-mediated apoptosis induction in SHEP, SKN-MC and SIHA was completely dependent on superoxide anions generated by NOX1 and seemed to be mediated by $^1O_2$. Like in MKN-45 cells, the $^1O_2$-mediated effect was fast and related to the long-lived species of CAP-treated medium. As these cell lines (like all tumor cell lines tested sofar (Bauer, 2014) are protected by membrane-associated catalase, targeting of catalase is the most likely effect of CAP on these cells. It is in line with the finding the inhibition of catalase by 3-AT, neutralizing antibodies or photofrin-derived $^1O_2$ leads to reactivation of NOX1-driven intercellular apoptosis-inducing signaling. It seems very likely that the very broad effect of CAP towards many different tumor systems is due to its interaction with membrane-associated catalase.

Supplementary Figure 6

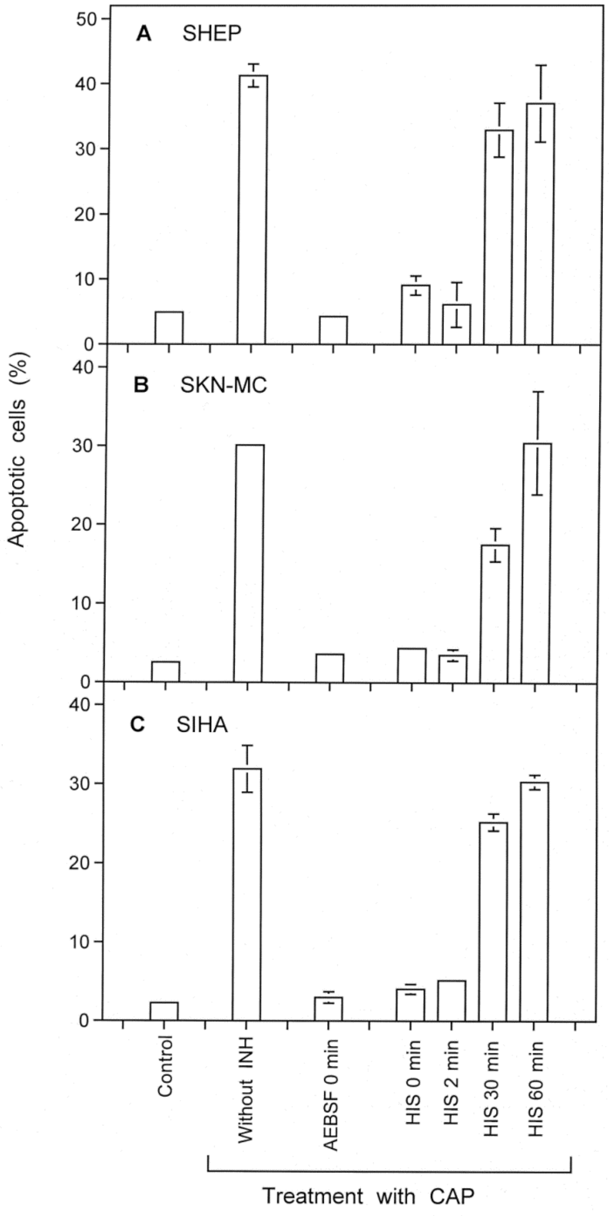

Supplementary Figure 6. *The effect of CAP on neuroblastoma, Ewing sarcoma and cervical carcinoma cells.* The human neuroblastoma (SHEP), Ewing sarcoma (SKN-MC) and cervical carcinoma (SIHA) cells were seeded in 24 well tissue culture clusters in 1 ml of medium. After the cells had attached to the plate, they remained untreated (control) or were treated with CAP for 1 min, in the absence or presence of the indicated inhibitors, added at the indicated times.

These data show that all three tumor cell lines responded to CAP treatment with apoptosis induction that was dependent on NOX1-derived superoxide anions and was mediated by a fast $^{1}O_2$-dependent step.

Statistical analysis: CAP-mediated apoptosis induction in the absence of inhibitor was highly significant for all three cell lines (p<0.001). Inhibition by AEBSF, Histidine (0 min) and Histidine (2 min) was highly significant (p<0.001). Inhibition by histidine added a 30 min was significant for SHEP cells (p<0.01), highly significant for SK-NMC cells (p<0.001) and not significant for SIHA cells. Addition of histidine at 60 min did not cause significant inhibition.

*6. The biological effects of CAP treatment in the **Spark** regime*

All experiments presented in the main manuscript and in Supplementary Figures 1-6 of the Supplement had been performed with CAP in the Corona regime. This final chapter shows that treatment of tumor cells with CAP in the Spark regime causes an analogous effect.

When MKN-45 human gastric carcinoma cells were treated with CAP in the Spark regime, apoptosis induction seemed to be due to enhancement of primary $^1O_2$ action through triggering secondary $^1O_2$ generation by the cells, as seen by the effect of transient inhibition of the process by AEBSF (Supplementary Figure 7). Also, like in Corona treatment, under conditions that prevented the generation of secondary $^1O_2$ and the generation of primary $^1O_2$ through interaction of NO_2^- with H_2O_2 (long-lasting components of PAM), $^1O_2$ derived directly from CAP seemed to be responsible for delayed apoptosis induction that started four hours after CAP treatment. This points to a relatively low concentration of direct $^1O_2$, in analogy to the finding obtained for Corona treatment.

Like in Corona treatment, treatment of tumor cells with PAM generated through application of CAP in the Spark regime allowed subsequent $^1O_2$ mediated apoptosis induction in tumor cells that was dependent on the reactivation of intercellular HOCl signaling, as it was inhibited by the HOCl scavenger taurine, the NOX inhibitor AEBSF and the hydroxyl radical scavenger mannitol (Supplementary Figure 8).

PAM generated through treatment of medium with CAP in the Spark regime caused $^1O_2$-mediated inactivation of membrane-associated catalase of tumor cells, as determined by sensitization of the cells for the apoptosis-inducing effect of an exogenous challenge with $ONOO^-$ (Supplementary Figure 9). The effect of

inactivation was comparable to the effect of the established catalase inhibitor 3-AT. $^1O_2$-mediated inactivation of catalase required the interaction between NOX-derived superoxide anions, H_2O_2, NOS-derived NO and $ONOO^-$, whereas HOCl played no role. These findings point to an analogous mechanism as found for catalase inactivation by Corona-dependent catalase inactivation, with an interaction between primary and secondary $^1O_2$ generation as mechanistic cause.

Comparison of apoptosis induction in MKN-45 cells by increasing concentrations of PAM generated either in the Corona or the Spark regime showed that in both cases concentration-dependent apoptosis was achieved. It seemed that PAM generated by Corona treatment was more efficient than PAM generated by Spark treatment. Whereas PAM generated by Corona treatment caused NOX1-dependent apoptosis induction over the whole range of concentrations applied, the apoptosis-induction by PAM generated through Spark was not dependent on NOX after longer exposure and at high concentrations of PAM applied (Supplementary Figure 10).

Supplementary Figure 7

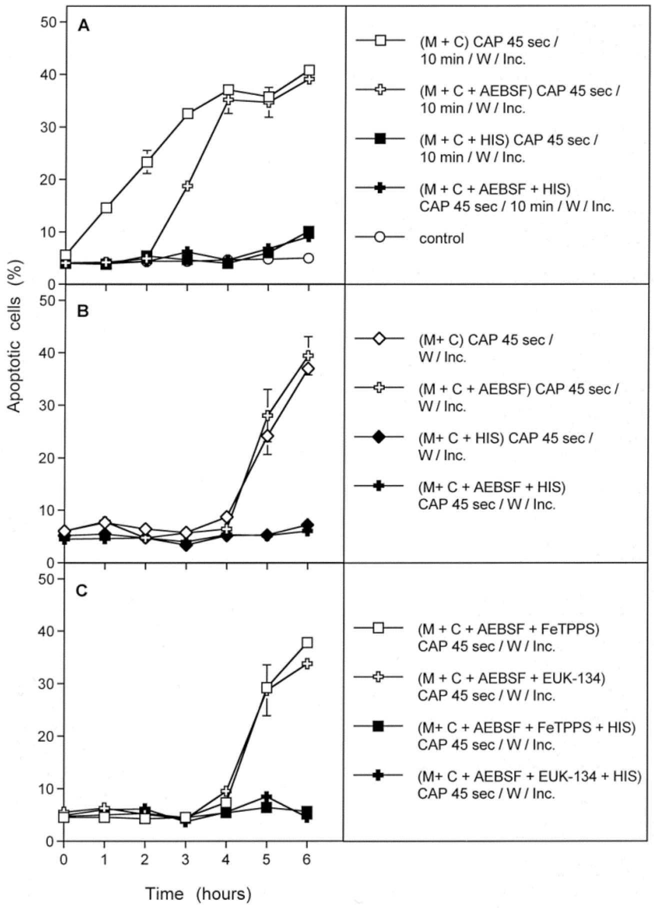

Supplementary Figure 7. *Primary and secondary $^1O_2$ after treatment of tumor cells with CAP in the SPARK regime.*

A. *When* MKN-45 cells were treated with CAP (SPARK regime) for 45 sec, followed by 10 min incubation in the same medium and two cycles of washing steps ((M+C)CAP 45 sec/ 10 min /W / Inc.), the cells showed an immediate start of time-dependent apoptosis induction. The presence of AEBSF during CAP treatment and 10 min incubation caused a delay in apoptosis induction, which is explained through the inhibition of secondary $^1O_2$ generation during this time period. Both processes were dependent on $^1O_2$, as shown by the strong inhibitory effect of histidine.

B. When tumor cells were treated with CAP (SPARK regime) for 45 seconds and then were immediately washed and resuspended in fresh medium, the kinetics of apoptosis induction did not start earlier than 4 h after initial treatment. The presence of AEBSF during CAP treatment did not affect the kinetics. The inhibition by histidine points to a $^1O_2$dependent step during CAP treatment.

C. When tumor cells were treated with CAP (SPARK regime) for 45 seconds in the presence of AEBSF plus FeTPPS, or AEBSF plus EUK-134, and thus the generation of primary $^1O_2$from long-lived species in CAP-treated medium, as well as the generation of secondary $^1O_2$by the tumor cells was prevented, the resultant kinetics of apoptosis induction was not different from that observed before under B. As the process was inhibited when histidine was present during initial CAP treatment, the delayed kinetics observed under the conditions of B and C seems to have been triggered by $^1O_2$ derived directly from CAP.

Statistical analysis: A: Apoptosis induction was highly significant at 1 h and later for the curve described by open squares and at 4 h and later for the curve described by open crosses. The inhibitory effect of histidine was highly significant (p<0.001). B, C: Apoptosis induction described by the curves with open circles became highly significant after 5 h (p<0.001). The inhibitory effect of histidine was highly significant (p<0.001).

Supplementary Figure 8

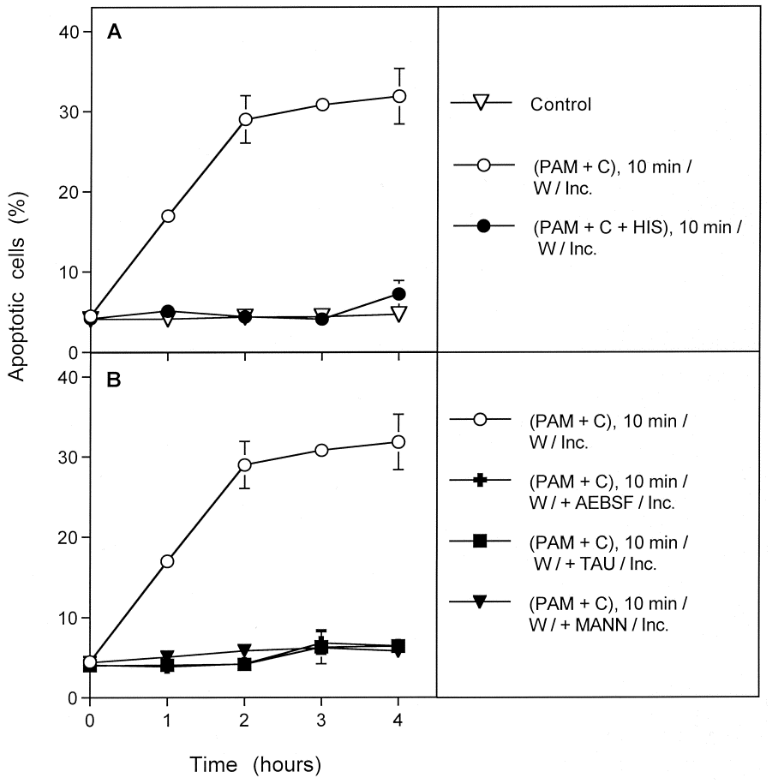

Supplementary Figure 8. *The effect of PAM generated by CAP in the SPARK regime.* PAM was generated by treating medium with CAP (SPARK regime) for 45 seconds. PAM was added to MKN-45 cells (80 percent PAM per assay) and the assays were incubated at 37°C for 10 min, before they were subjected to 3 cycles of washing. Apoptosis induction was determined kinetically. When histidine had been presented during the contact of PAM with the cells (A), apoptosis was completely blocked, indicating that the effect mediated by PAM was triggered by $^1O_2$. B. The presence of either AEBSF, taurine or mannitol after the washing step caused complete inhibition of apoptosis induction, demonstrating that it was mediated by HOCl signaling.

Statistical analysis: PAM-mediated apoptosis induction was highly significant after 1 h ($p<0.001$). The effects of all inhibitors were highly significant ($p<0.001$).

Supplementary Figure 9

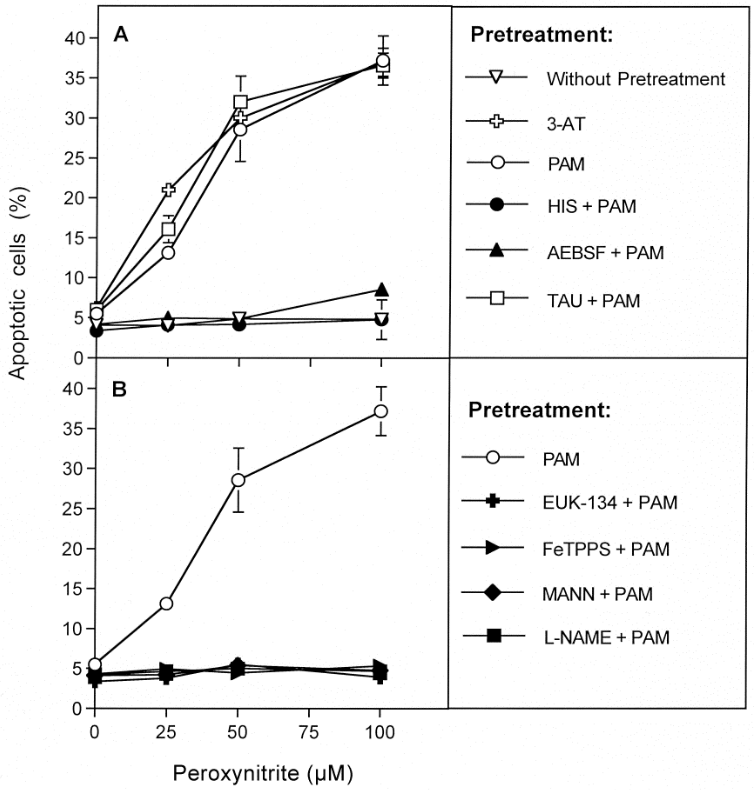

Supplementary Figure 9. *PAM generated by CAP in the SPARK regime causes inactivation of membrane-associated catalase of tumor cells.* PAM was generated through treatment of medium with CAP (SPARK regime) for 50 sec. When MKN-45 cells were treated with 50 % PAM for 15 min, followed by three cycles of washing and a $ONOO^-$ challenge at the indicated concentrations, the cells went into apoptosis to the same degree as cells that had been pretreated with the catalase inhibitor 3-AT. The presence of histidine, AEBSF, EUK-134, FeTPPS, mannitol or L-NAME during the time of PAM/tumor cell interaction completely prevented sensitization for the PON challenge, indicating that the inactivation of catalase by PAM was due to $^1O_2$, NOX-derived superoxide anions, H_2O_2, $ONOO^-$, hydroxyl radicals and NOS-derived NO. HOCl did not play any role for this step, as seen by the lack of inhibition by taurine. These findings show that catalase inactivation by PAM generated by CAP in the SPARK regime causes the same biochemical effects as PAM generated by CAP in the CORONA regime (please see Figure 4 **b** of the main manuscript).
Statistical analysis: A. Peroxynitrite applied at 25 µM and higher caused highly significant apoptosis induction in assays containing cells pretreated with 3-AT, PAM or PAM plus taurine (p<0.001). There was no significant apoptosis induction in the assays containing untreated control cells or cells pretreated with PAM in the presence of histidine or AEBSF.
B: Apoptosis induction in cells pretreated with PAM was highly significant at 25 µM peroxynitrite or higher (p<0.001). The inhibitory effect by all inhibitors was highly significant (p<0.001).

Supplementary Figure 10

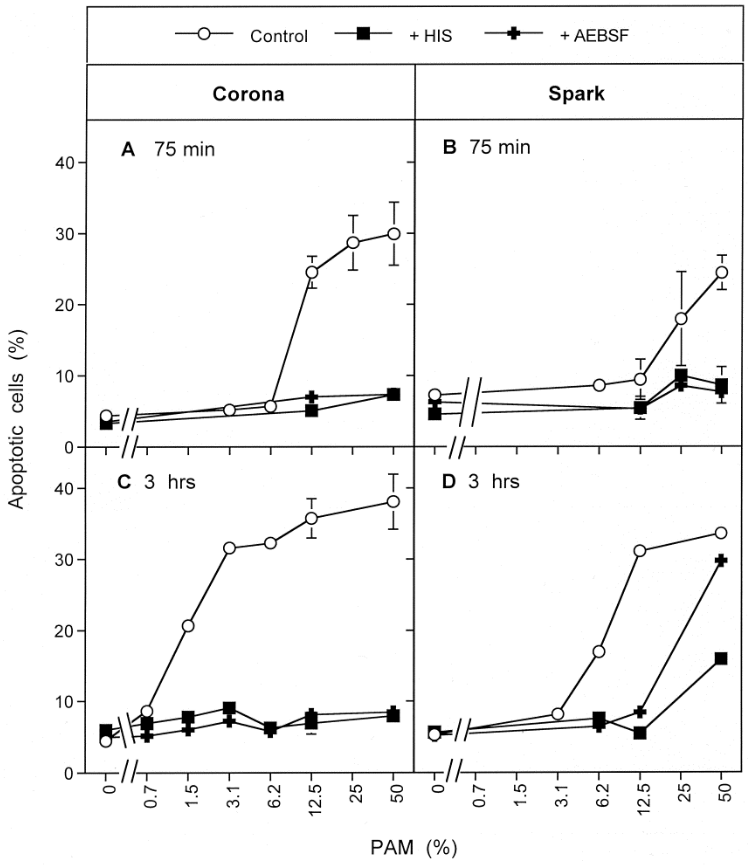

Supplementary Figure 10. *The effects of PAM generated by the CORONA or the SPARK regime.* PAM was generated through the treatment of medium with CAP for 1 min, either in the CORONA or the SPARK regime. After 10 min, the indicated concentrations of PAM were added to MKN-45 cells in the absence or presence of histidine or AEBSF. The percentages of apoptosis induction were determined at the indicated times.

The results confirm the leftward shift of the concentration-dependency of apoptosis induction. PAM-mediated apoptosis induction is dependent on $^1O_2$ and superoxide anions for PAM generated by each one of the two regimes, with the exception of the highest concentration of PAM (SPARK) at 3 h.

Statistical analysis: The dependencies of apoptosis induction on the concentration of PAM were different with high significance when the effects of Corona and Spark regime were compared at both time points, or when each regime was analyzed at different time points (p<0.001).

C. Supplementary Discussion

1. The detection of primary $^1O_2$ *derived directly from CAP*

The data presented in the main manuscript show that the effects of CAP and PAM under standard conditions (which ensure selective action towards tumor cells) are sufficiently explained by the triggering effect of low concentrations of primary $^1O_2$ generated through the interaction of the long-lived species H_2O_2 and NO_2^- contained in CAP-treated medium and the subsequent sustained generation of secondary $^1O_2$ by the tumor cells. This leads to sufficient inactivation of protective catalase and allows reactivation of apoptosis-inducing HOCl signaling.

However, it is conceivable that under conditions of CAP treatment of tumor cells in suspension (like MKN-45), an even lower concentration of primary $^1O_2$ directly derived from CAP might act on tumor cells in addition to the prevailing $^1O_2$ generated through the interaction of NO_2^- and H_2O_2.

Supplementary Figure 11 shows that prevention of secondary $^1O_2$ generation through inhibition of NOX1 by AEBSF selects for catalase inactivation by primary $^1O_2$. Provided the concentration of primary $^1O_2$ is sufficiently high, this inactivation might be detectable through an exogenous challenge with $ONOO^-$, as shown in Figures 5**a** and 5**b** in the main manuscript.

If in addition to inhibition of NOX1 by AEBSF the generation of primary $^1O_2$ through the interaction of PAM compounds is prevented by either EUK-134 or FeTPPS, inactivation of catalase would be indicative for the action of $^1O_2$ derived from CAP (Supplementary Figure 12). Its nature would be verified by inhibition of catalase inactivation by histidine (Supplementary Figure 13).

Supplementary Figure 11

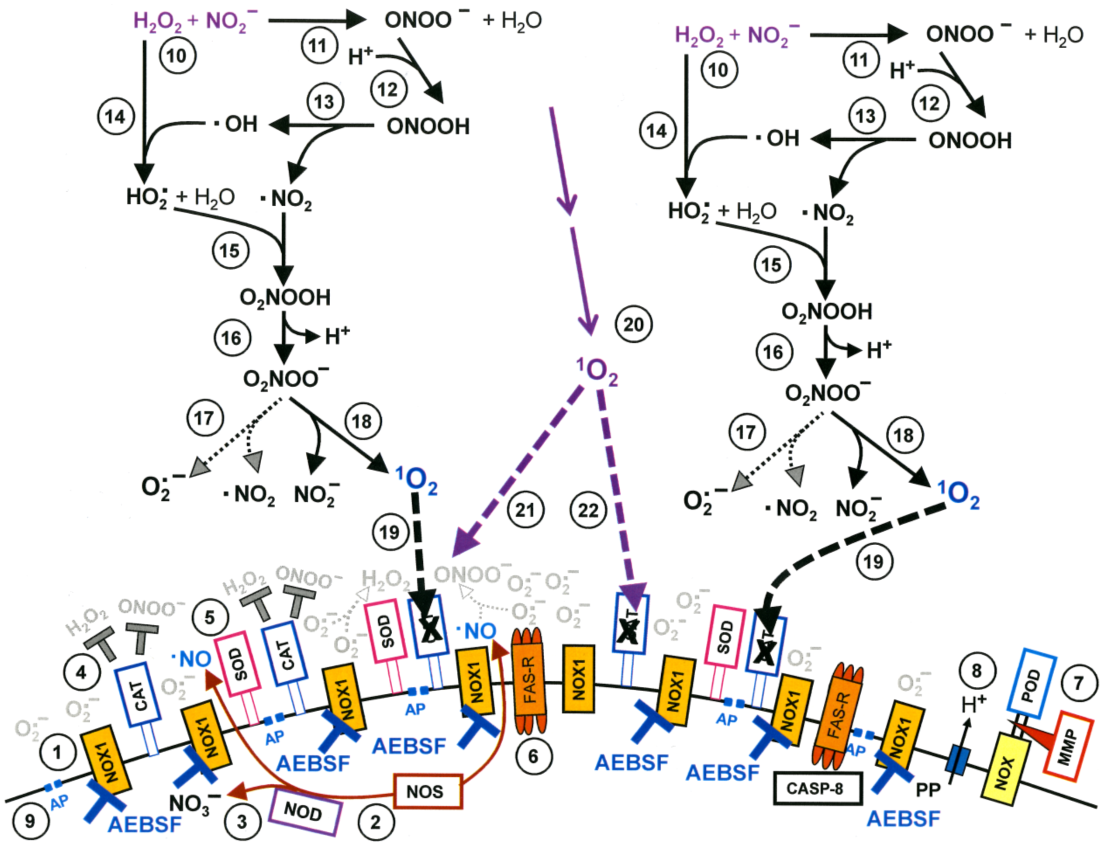

Supplementary Figure 11. *Primary* $^1O_2$*and catalase inactivation.*
NADPH oxidase 1 (NOX1) is expressed in the membrane of malignant cells and generates extracellular superoxide anions (#1). NO synthase (NOS) (#2) generates NO which can be either oxidised by NO dioxygenase (NOD) (#3) or pass through the cell membrane. Membrane-associated catalase (#4) protects tumor cells towards intercellular ROS/RNS-mediated signaling. Comodulatory SOD (#5) is required to prevent superoxide anion-mediated inhibition of catalase. Further important elements in the membrane are the FAS receptor (#6), Dual oxidase (DUOX) (#7), from which a peroxidase domain is split through matrix metalloprotease, proton pumps (#8) and aquaporins (#9). When the generation of secondary $^1O_2$ is inhibited by AEBSF, catalase can be selectively inactivated by primary $^1O_2$ that might be generated from long-lived molecular species contained in CAP-treated medium (reactions # 10 -#19) or that might directly be supplied by CAP (#20 - #22).
[AEBSF inhibits the generation of secondary $^1O_2$ *as it prevents the generation of* H_2O_2 *and of* $ONOO^-$*, which are required for the initial reaction during the generation of secondary* $^1O_2$*.]*

Supplementary Figure 12

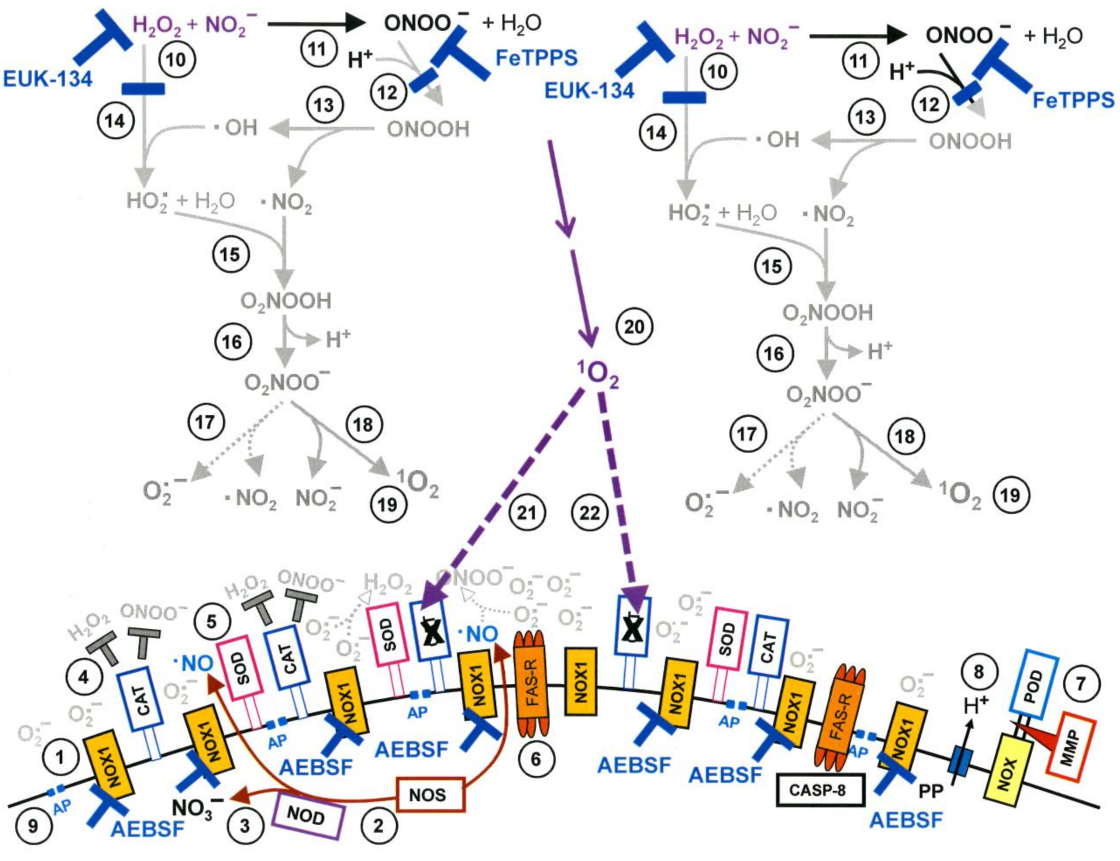

Supplementary Figure 12. Demonstration of catalase inactivation by primary $^1O_2$derived directly from CAP.

In the presence of AEBSF and EUK-134 or FeTPPS the generation secondary $^1O_2$ by the tumor cells and of primary $^1O_2$ from long-lived components of CAP-treated medium are prevented. Only $^1O_2$ derived directly from CAP has a chance to inactivate catalase under these conditions.

Supplementary Figure 13

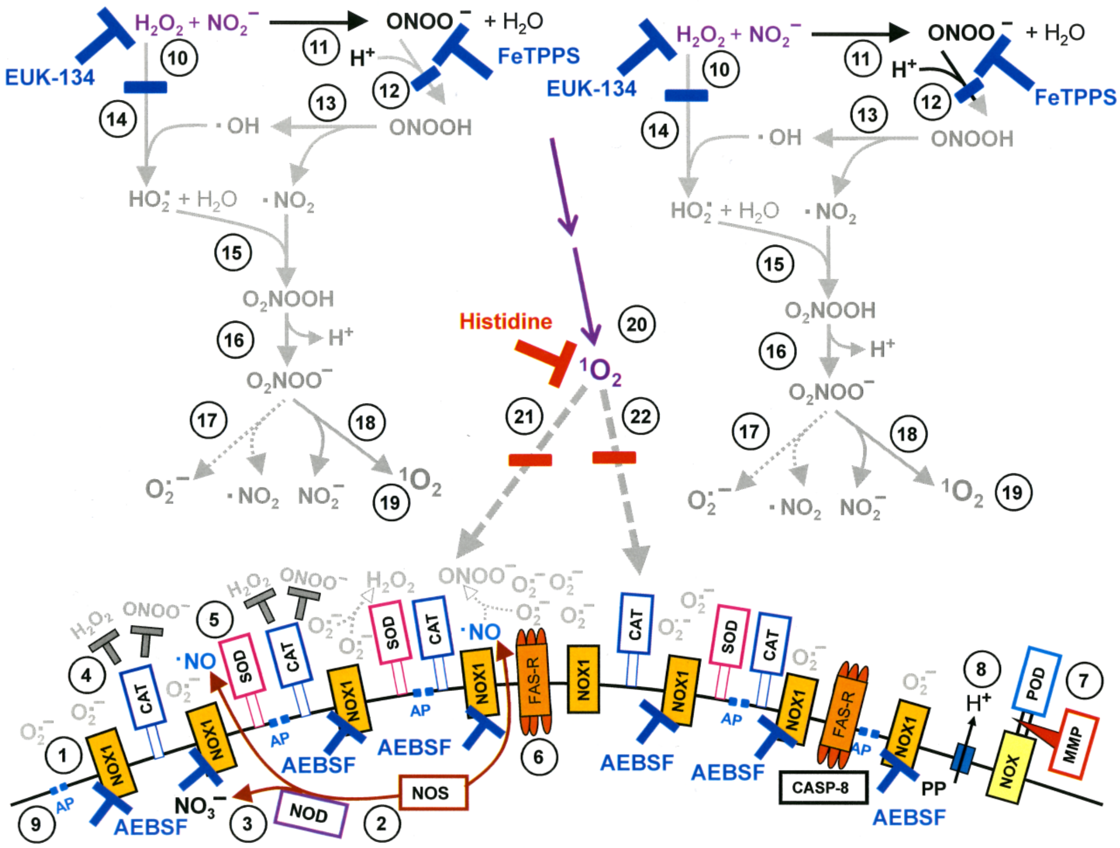

Supplementary Figure 13. *Verification of the nature of primary* $^1O_2$*derived from CAP. The inactivating effect of primary* $^1O_2$*derived from CAP in the presence of AEBSF plus EUK-134 or FeTPPS is prevented by histidine.*

2. CAP, PAM and immunogenic cell death

Apoptosis induction in tumor cells after CAP and PAM treatment in vitro, as well as with model compounds H_2O_2/ NO_2^- in vitro is characterized by a kinetics that is determined by the dominant steps of primary and secondary $^1O_2$ generation, catalase inactivation, aquaporin action to deplete intracellular GSH and intercellular apoptosis-inducing ROS signaling. The resultant kinetics regularly shows with a characteristic initial lag phase and reaches a plateau after a continuous increase in apoptotic cells.

Reaching a plateau rather than continuous increase in the percentage of apoptotic cells is due to a) decrease in ROS-dependent apoptosis induction as the density of cells is decreased through apoptosis induction; b) decomposition and even phagocytosis of apoptotic cells by their intact neighbouring cells, c) depletion of essential compounds in medium like glutamin and polyunsaturated fatty acids. The characteristics of the kinetics of apoptosis induction triggered by CAP and PAM in vivo is unknown so far. However, in view of our present knowledge on immunological control of tumors, it is not expected that ROS signaling is solely responsible for eventual complete tumor regression. Rather it is likely to trigger and reactivate the innate and adaptive immune system.

It seems that CAP- and PAM-dependent selective apoptosis induction in tumor cells has the characteristics of immunogenic cell death, i. e. it stimulates dendritic cells and thus provokes a subsequent activation of cytotoxic T cells (Lin et al., 2015, 2017 a, b, 2018; Miller et al., 2017; Bekeschus et al., 2018 a, b; Mizuno et al., 2017). In addition, CAP provokes activation of cytotoxic macrophages (Kaushik et al., 2016) The findings that cell death induced by CAP and PAM may trigger an additional strong and selective immunological antitumor effect that is far-ranging within the tumor as well as in the body, is in perfect agreement with the general concept that immunological cell death is a necessary element to finalize tumor treatment by various antitumor agents (Apetoh et al., 2008; Green et al., 2009; Krysko et al., 2012; Kroemer et al., 2013; Garg et al., 2014; Candeias and Gaipl, 2016).
The role of HOCl seems not be restricted to apoptosis induction through the HOCl signaling pathway. Due to its high reactivity, HOCl can also modify tumor cell antigens very efficiently and enhance T cell responses directed towards tumor cells (Chiang et al., 2006, 2008, 2015; Prokopowicz et al., 2010; Biedron et al., 2015). It is

conceivable that HOCl-dependent antigenic modification may act in concert with canonical DAMPS that are involved in classical immunogenic cell death that triggers a T cell response towards tumor cells. There is a general consent that T cell responses after initial tumor treatment are of central importance for the overall outcome of most if not all antitumor therapies presently under investigation (Apetoh et al., 2008; Green et al., 2009; Krysko et al., 2012; Kroemer et al., 2013; Garg et al., 2014; Candeias and Gaipl, 2016).

D. Supplementary References

Apetoh, L., Tesnier, A., Ghiringhelli, F., Kroemer, G. & Zitvogel, L. Interactions between dying tumor cells and the innate immune system determine the efficiency of conventional antitumor therapy. *Cancer Res*.**68**, 4026-4030 (2008).

Bauer, G. & Motz, M. The antitumor effect of single-domain antibodies directed towards membrane-associated catalase and superoxide dismutase. *Anticancer Res*. **36**, 5945-5956 (2016).

Bauer, G. Central signaling elements of intercellular reactive oxygen/nitrogen species-dependent induction of apoptosis in malignant cells. *Anticancer Res.* **37**, 499-514 (2017).

Bauer, G. Cold Atmospheric Plasma and Plasma-Activated Medium: Antitumor Cell Effects with Inherent Synergistic Potential. Plasma Medicine, in press, 2019

Bauer, G. Increasing the endogenous NO level causes catalase inactivation and reactivation of intercellular apoptosis signaling specifically in tumor cells. *Redox Biol,* **6**, 353-371 (2015).

Bauer, G. Signal amplification by tumor cells: clue to the understanding of the antitumor effects of cold atmospheric plasma and plasma-activated medium. *IEEE Transactions on Radiation and Plasma Medical Sciences* **2**, 87-98 (2018).

Bauer, G. SiRNA-based analysis of the abrogation of the protective function of membrane-associated catalase of tumor cells. *Anticancer Res*. **37**: 567-582 (2017).

Bauer, G. Targeting extracellular ROS signaling of tumor cells. *Anticancer Res*. **34**, 1467-1482 (2014).

Bekeschus, S., Clemen, R., Metelmann, H.-R. Potentiating anti-tumor immunity with physical plasma. *Clinical Plasma Medicine* **12**,17-22 (2018).

Bekeschus, S., Iseni, S., Reuter, S., Masur, K. & Weltmann, K.-D. Nitrogen shielding of an

argon plasma jet and its effects on human immune cells. *IEEE Transactions on plasma science* 43, 776-781 (2015). DOI: 10.1109/TPS.205.2393379

Bekeschus, S., Masur, K., Kolata, J., Wende, K., Schmidt, A., Bundscherer, L., Barton, A., Kramer, A., Bröker, B. & Weltmann, K.-D. Human mononuclear cell survival and proliferation is modulated by cold atmospheric plasma jet. *Plasma Process. Polym*. **10**, 706-713 (2013).

Bekeschus, S., Mueller, A., Miller, V., Gaipl, U. & Weltmann, K.-D. Physical plasma elicits immunogenic cancer cell death and mitochondrial singlet oxygen. *IEE transactions on Radiation and Plasma Medical Sciences* **2**,138-147 (2018).

Biedron, R., Konopinski, K., Marcinkiewicz, J. & Jozefowski ,S. Oxidation by neutrophil-derived HOCl increases immunogenicity of proteins by converting them into ligands of several endocytic receptors involved in antigen uptake by dendritic cells and macrophages. *PLOS ONE* **10**, e01123293 (2015).

Bundscherer, L., Bekeschus, S., Tresp, H., Hasse, S., Reuter, S., Weltmann, K.-D., Lindequist, U. & Masur, K. Viability of human blood leucocytes compared with their respective cell lines after plasma treatment. *Plasma Medicine* **3**, 71-80 (2013).

Selleri, C., Sato, T., Raiola, A. M., Rotoli, B., Young, N.S. & Maciejewski, J.P. Induction of nitric oxide synthase is involved in the mechanism of FAS-mediated apoptosis in hematopoietic cells. *Br J Hematol,* **99**, 481-489 (1997).

Canal, C., Fontelo, R, Hamouda, I., Guillem-Marti ,J., Cvelbar, U., Ginebra & M.-P. Plasma-induced selectivity in bone cancer cell death. *Free Radic Biol & Med*. **110**, 72-80 (2017).

Candeias, S.M. & Gaipl, U.S. The immune system in cancer prevention, development and therapy. *Anticancer Agents in Medicinal Chemistry* **16**,101-107 (2016).

Chiang, C.L.L., Coukos, G. & Kandalaft, L.E. Whole tumor antigen vaccines: where are we?. *Vaccines*, **3**: 344-372 (2015).

Chiang, C.L.L., Ledermann, J.A., Aitkens, E., Benjamin, E., Katz, D.R. & Chain, B.M. Oxidation of ovarian epithelial cancer cells by hypochlorous acid enhances immunogenicity and stimulates T cells that recognize autologous primary tumor. *Clin. Cancer Res*. **14**, 4898-4907 (2008).

Chiang, C.L.L., Ledermann, J.A., Rad, A.N., Katz, D.R. & Chain, B.M. Hypochlorous acid enhances immunogenicity and uptake of allogeneic ovarian tumor cells by dendritic cells to cross-prime tumor-specific T cells. *Cancer Immunol. Immunother*. **55**, 1384-1395 (2006).

Duan, J., Lu, X. & He, G. The selective effect of plasma-activated medium in an in vitro co-culture of liver cancer and normal cells. *J. Appl. Phys*. **121**, 013302 (2017).

Fridman, G., Friedman, G., Gutsol, A., Shekhter, A.B., Vasilets, V.N. & Fridman, A. Applied plasma medicine. *Plasma Process. Polym*. **5**, 503-533 (2008).

Garg, A.D. & Agostinos, P. ER stress, autophagy and immunogenic cell death in photodynamic therapy-induced anti-cancer immune responses. *Photochem. Photobiol. Sci.* **13**, 474-487 (2014).

Gianela, M., Reuter, S., Aguila, A.L., Ritchie, G.A.D. & van Helden, J.P.H. Detection of HO_2 in an atmospheric pressure plasma jet using optical feedback cavity-enhanced absorption spectroscopy. *New J. Physics* **18**, 113027 (2016).

Graves, D.B. Mechanisms of Plasma Medicine: Coupling Plasma Physics, Biochemistry, and Biology. *IEEE Transactions on Radiation and Plasma Medical Sciences* **1**, 281-292 (2017).

Graves, D.B. Oxy-nitroso shielding burst model of cold atmospheric plasma therapeutics. Clin. Plasma Med. **2**,38-49 (2014)

Graves, D.B. Reactive species from cold atmospheric plasma: implications for cancer therapy. Plasma Process. Polym. **11**, 1120-1127 (2014).

Graves, D.B. The emerging role of reactive oxygen and nitrogen species in redox biology and some implications for plasma applications to medicine and biology. *J. Phys. D: Appl. Phys*. **45**, 263001 (2012).

Green, D.R., Ferguson, T., Zitvogel, L. & Kroemer, G. Immunogenic and tolerogenic cell death. *Nature Rev. Immunol*. **9**, 353–363 (2009)

Guerrero-Preston, R., Ogawa, T., Uemura, M., Shumulinsky, G., Valle, B.L., Pirini, F., Ravi, R,, Sidransky, D., Keidar, M. & Trink, B. Cold atmospheric plasma treatment selectively targets head and neck squamous cell carcinoma cells. Int. J. Mol. Med. **34**, 941-946 (2014).

Hirst, A.M., Simms, M.S., Mann, V.M., Maitland, N.J., O`Connell, D. & Frame, F.M. Low temperature plasma treatment induces DNA damage leading to necrotic cell death in primary prostate epithelial cells. Br. J- Cancer **112**, 1536-1545 (2015) DOI: 10.1038/bjc.2025.113.

Jablonowski, H., Bussiahn, R., Hammer, M.U., Weltman, K.-D., von Woedtke, T. & Reuter, S. Impact of plasma jet vacuum ultraviolet radiation on reactive species generation in bio-relevant liquids. Physics of plasmas **22**, 122008 (2015).

Jirásek, V. & Lukes, P. Formation of reactive chlorine species in saline solution treated by non-equilibrium atmospheric pressure He/O_2 plasma jet. *Plasma Sources Sci. Technol* **28**, 03015 (2019)

Kalghati, S., Kelly, C., Cerchar, E. & Azizkhan-Clifford, J. Selectivity of non-thermal atmospheric-pressure microsecond-pulsed dielectric barrier discharge plasma induced apoptosis in tumor cells over healthy cells. *Plasma Medicine* **1**: 249-263 (2011).

Kaushik, N., Kumar, N., Kim, C.H., Kaushik, N. & Choi, E.H. Dielectric barrier discharge plasma efficiently delivers an apoptotic response in human monocytic lymphoma. Plasma Process Polymer. **11**, 1175-1187 (2014) Doi: 10.1002/ppap.201400102.

Kaushik, N.K., Kaushik, N., Min, B., Choi, K.H., Hong, Y.J. & Miller. V., Fridman A, Choi EH. Cytotoxic macrophage-released tumour necrosis factor-alpha (TNF-alpha) as a killing mechanism for cancer cell death after cold plasma activation. *J Phys D-Appl Phys*. **49**, 084001 (2018).

Kim, S.J. & Chung, T.H. Cold atmospheric plasma jet-generated RONS and their selective effects on normal and carcinoma cells. Sci. Rep. **6**, 20332 (2016) DOI: 10.1038/srep.20332.

Kroemer, G., Galluzzi, L., Kepp, O. & Zitvogel, L. Immunogenic cell death in Cancer therapy. *Ann. Rev. Immunol*. **31**:51-72 (2013)

Krysko, D.V., Garg, A.D., Kaczmarek, A., Krysko, O., Agostinis, P. & Vandenabeele, P. Immunogenic cell death and DAMPs in cancer therapy. *Nature Rev. Cancer* **12**, 860-875 (2012).

Laroussi, M., Lu, X. & Keidar, M. Perspective: The physics, diagnostics, and applications of atmospheric pressure low temperature plasma sources used in plasma medicine. *J. Appl. Physics.***122**, 020901 (2017).

Laroussi, M. From killing bacteria to destroying cancer cells: 20 years of plasma medicine. *Plasma Process. Polymers* **11**,1138-1141 (2014).

Laroussi, M. Low-temperature plasma for medicine. *IEEE Transactions on Plasma Science* **37**,714-715 (2009).

Laroussi, M. Low-temperature plasma jet for biomedical applications: a review. *IEEE transactions on plasma science* **43**, 703-712 (2015).

Liedke, K.R., Bekeschus, S., Kaeding, A., Hackbarth, C., Kuehn, J.P., Heidecke, C.D., von Bernstorff, W., von Woedtke, T. & Partecke, L.I. Non-thermal plasma-treated solution demonstrates antitumor activity against pancreatic cancer cells in vitro and in vivo. Sci. Rep. **7**, 8319 (2017) DOI 10.1038/sa1598-017-08560-3.

Lin, A, Truong, B., Fridman, G., Fridman, A. & Miller, V. Immune cells enhance selectivity of nanosecond-pulsed DBD plasma against tumor cells. *Plasma Medicine* **7**,85-96 (2017).

Lin, A., Truong, B., Pappas, A., Kirifides, L., Oubarri, A., Chen, S., Lin, S., Dobrynin, D., Fridman, G., Fridman, A., Sang, N. & Miller, V. Uniform nanosecond pulsed dielectric barrier discharge plasma enhances anti-tumor effects by induction of immunogenic cell death in tumors and stimulation of macrophages. *Plasma Process. Polymers* **12**,1392-1399 (2015).

Lin, A., Truong, B., Patel, S., Kaushik, N., Choi, E.H., Fridman, G., Fridman, A. & Miller, V. Nanosecond-pulsed DBD plasma-generated reactive oxygen species trigger immunogenic cell death in A549 lung carcinoma cells through intracellular oxidative stress. *Int. J. Mol. Sciences* **18**, 966 (2017).

Lin, A.G., Xiang, B., Merlino, D.J., Baybutt, T.R., Sahu, J., Fridman, A., Snook, A.E. & Miller, V. Non-thermal plasma induces immunogenic cell death in vivo in murine CT26 colorectal tumors. *Oncoimmunology* **7**, e148978 (2018).

Lu, X., Naidis, G.V., Laroussi, M., Reuter, S., Graves, D.B. & Ostrikov, K. Reactive species in non-equilibrium atmospheric-pressure plasmas: Generation, transport, and biological effects. *Physics Reports-Review Section of Physics Letters* **630**,1-84 (2016).

Machala, Z., Tarabova, B., Hensel, K., Spetlikova, E., Sikurova, L. & Lukes, P. Formation of ROS and RNS in Water electro-Sprayed through Transient Spark Discharge in Air and their Bactericidal Effects. *Plasma Process. Polym*. **10**, 649–659 (2013).

Machala, Z , Janda, M., Hensel, K., Jedlovsky, I., .Lestinska, L., Foltin, V., Martisovits, V. & Morvova, M. Emission spectroscopy of atmospheric pressure plasmas for bio-medical and environmental applications. *J. Mol. Spectr*.**243**,194-201 (2007).

Miller, V., Lin, A. & Fridman, A. Why target immune cells for plasma treatment of cancer. *Plasma Chem. Plasm. Process* **36**,259-268 (2016)

Mizuno, K., Yonetamari, Y., Shirakawa, Y., Akiyama, T. & Ono, R. Anti-tumor immune response induced by nanosecond pulsed streamer discharge in mice. *J. Phys. D-Appl Phys*. **50**,12LT01 (2017).

Prokopowicz, Z.M., Arce, F., Biedron, R., Chiang, C.L.L., Ciszek, M., Katz, D.R.,

Nowakowska, M., Zapotoczny, S., Marcinkiewicz, J. & Chain, B.M. Hypochlorous acid: a natural adjuvant that facilitates antigen processing, cross-priming, and the induction of adaptive immunity. *J. Immunol*. **184**, 824-835 (2010).

Reinehr, R., Becker, S., Eberle, A., Grether-Beck, S. & Häussinger, D. Involvement of NADPH oxidase isoforms and src family kinases in CD95-dependent hepatocyte apoptosis. *J Biol. Chem*. 280, 27179-27194 (2005).

Zhuang, S., Demir, J. T. & Kochevar, I. E., Protein kinase C inhibits singlet oxygen-induced apoptosis by decreasing caspase-8 activation. *Oncogene* **20**, 6764-6776 (2001).

Schmidt-Bleker, A., Bansemer, R., Reuter, S. & Weltmann, K.-D. How to produce a NOx- instead of Ox-based chemistry with a cold atmospheric plasma jet. *Plasma Process. Polymer*. **13**,1120-1127 (2016).

Siu, A., Volotskova, O., Cheng, X., Khaisa, S.S., Bian, K., Murad, F. & Keidar, M, Sherman JH. Differential effects of cold atmospheric plasma in the treatment of malignant glioma. *PLOS ONE* **10**, e0126313 (2015). Doi: 10.1371/journal.pone.0126313.

Sousa, J.S., Niemi, K., Cox, L.J., Algwari, Q.T., Gans, T. & O`Connell, D. Cold atmospheric pressure plasma jets as sources of singlet delta oxygen for biomedical applications. *J. Appl. Physics* **109**,123302 (2011).

Stoffels, E., Sakiyama, Y. & Graves, D.B. Cold atmospheric plasma: charged species and their interactions with cells and tissues. *IEEE Transactions on Plasma Science* **36**,1441-1457 (2008).

Tanaka, H., Ishikawa, K., Nakamura, K., Kajiyama, H., Komo, H., Kikkawa, T. & Hori, M. Plasma-activated medium selectively kills glioblastoma brain tumor cells by down-regulating a survival signaling molecule, AKT kinase. *Plasma Med*. **1**, 265-277 (2011). DOI: 10.1615/PlasmaMed.2012006275.

Utsumi, F., Kajiyama, H., Nakamura, K., Tanaka, H., Hori, M. & Kikkawa, F. Selective cytotoxicity of indirect nonequilibrium atmospheric pressure plasma against ovarian clear-cell carcinoma. *SpringerPlus* **3**, 398-316 (2014) DOI: 10.1186/2193-1801-3-398.

Wang, M., Holmes, B., Cheng, X., Zhu, W. & Keidar, M, Zhang LG. Cold atmospheric plasma for selectively ablating metastatic breast cancer cells. *PLOS One* **8**, e73741 (2013).

Wende, K., Straßenburg, S., Haertel, B., Harms, M., Holtz, S., Barton, A., Masur, K., von Woedtke, T. & Lindequist, U. Atmospheric pressure plasma jet treatment evokes transient oxidative stress in HaCat keratinocytes and influences cell physiology. *Cell Biol. Int*. **38**, 412-425 (2014).

Wende, K., Williams, P., Dalluge, J., Van Gaens, W., Akoubakr, H., Bischof ,J., von Woedtke, T., Goyal, S.M., Weltmann, K.-D., Bogaerts, A., Masur, K. & Bruggeman, P.J. Identification of biologically active liquid chemistry induced by nonthermal atmospheric pressure plasma jet. *Biointerphases*. **10**, 029518 (2015).

Suzuki, Y., Ono,Y. & Hirabayashi Y. Rapid and specific reactive oxygen species generation via NADPH oxidase activation during FAS-mediated apoptosis”, *FEBS letters* **425**, 209-212 (1998).

Zucker, S.N., Zirnheld, J., Bagati, A., DiSanto, T.M., Des Soye, B., Wawrzyniak, J.A., Eternadi, K., Nikiforov, M. & Berezney, R. Preferential induction of apoptotic cell death in

melanoma cells as compared with normal keratinocytes using a non-thermal plasma torch. *Cancer Biol. Therapy*. **13**, 1299-1306 (2012) DOI: 10.4161/cbt.21787.
